# Steering from electrochemical denitrification to ammonia synthesis

**DOI:** 10.1038/s41467-023-35785-w

**Published:** 2023-01-07

**Authors:** Huan Li, Jun Long, Huijuan Jing, Jianping Xiao

**Affiliations:** 1 State Key Laboratory of Catalysis, Dalian Institute of Chemical Physics, Dalian National Laboratory for Clean Energy, Chinese Academy of Sciences, Dalian, 116023 P. R. China; 2 University of Chinese Academy of Sciences, Beijing, 100049 P. R. China

**Keywords:** Electrocatalysis, Catalytic mechanisms, Theoretical chemistry

## Abstract

The removal of nitric oxide is an important environmental issue, as well as a necessary prerequisite for achieving high efficiency of CO_2_ electroreduction. To this end, the electrocatalytic denitrification is a sustainable route. Herein, we employ reaction phase diagram to analyze the evolution of reaction mechanisms over varying catalysts and study the potential/pH effects over Pd and Cu. We find the low N_2_ selectivity compared to N_2_O production, consistent with a set of experiments, is limited fundamentally by two factors. The N_2_OH* binding is relatively weak over transition metals, resulting in the low rate of as-produced N_2_O* protonation. The strong correlation of OH* and O* binding energies limits the route of N_2_O* dissociation. Although the experimental conditions of varying potential, pH and NO pressures can tune the selectivity slightly, which are insufficient to promote N_2_ selectivity beyond N_2_O and NH_3_. A possible solution is to design catalysts with exceptions to break the scaling characters of energies. Alternatively, we propose a reverse route with the target of decentralized ammonia synthesis.

## Abstract (web summary)

Electrocatalytic denitrification is a sustainable route for nitric oxide removal. However, transition metals consistently show low N_2_ selectivity while high N_2_O selectivity. Here the authors study the reaction phase diagram and kinetics over varying catalysts to understand the selectivity for NO electroreduction.

## Introduction

**Fig. 1:**
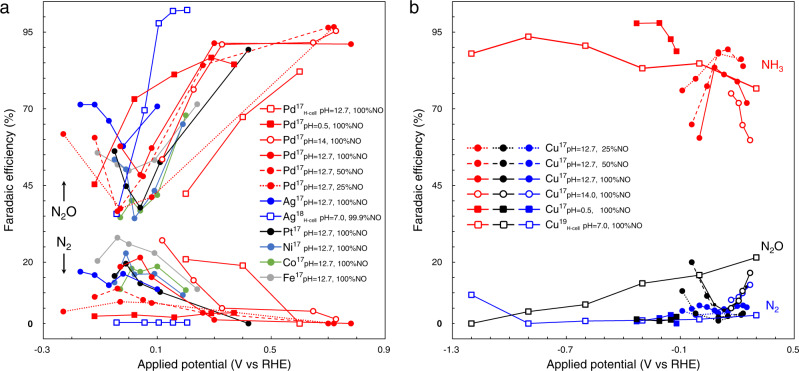
Overview of different catalysts in eNORR compiled from the recent reports^17–19^. **a** The Faradaic efficiency (FE) of N_2_O and N_2_ at low overpotentials (0.8 to −0.3 V vs. RHE) on Pd, Ag, Pt, Ni, Co, and Fe. **b** A higher FE of NH_3_ than dual-N products (N_2_O/N_2_) on Cu in a wider potential window (from 0.3 to −1.2 V vs. RHE). Most data points were measured in a flow-cell, while the open square symbols are detected values in a batch-type cell (H-cell). The data points for N_2_, N_2_O, and NH_3_ from the same experimental conditions were connected, respectively.

Modern human activities, such as the excessive use of fertilizers, fossil-fuels combustion, and the discharge of industrial wastewater, have disrupted the ecological environment. As one of the major pollutants, nitrogen oxides (NO_*x*_) cause the damages of aquatic ecosystem, drinking water, photochemical smog, ozone depletion, and acid rain, posing threats to human health and the Earth’s ecosystems^1,2^. Furthermore, carbon dioxide electroreduction (eCO_2_RR) is able to relieve global warming and is considered as an attractive route for sustainable production of fuels and chemicals^3^. However, it is usually unpractical to perform eCO_2_RR with a pure CO_2_ feed. It was found a trace of nitric oxide (NO) (~0.8%) in feed greatly lower the efficiency by ~30%^4^. Therefore, the removal of nitrogen oxides has been recognized to be a necessary prerequisite for eCO_2_RR. Although the NO_*x*_ can be thermochemically converted to N_2_ by conventional denitrification technology, i.e. selective catalytic reduction (SCR)^5^. However, NO electroreduction reaction (eNORR)^6–16^ should be a more compatible way with eCO_2_RR. Unfortunately, a high N_2_ selectivity was not achieved experimentally over all studied catalysts, as illustrated in Fig. 1, compiled from the recent literatures^17–19^.

At ~0.7 V vs. RHE, the observed N_2_O selectivity can be up to 95%, but with negligible N_2_ production on Pd. As the electrode potential drops to ~0 V vs. RHE, the N_2_O selectivity is still higher compared to N_2_ on Pd, Ag, Pt, Ni, Co, and Fe. In case the eNORR (on Pd and Ag) is performed in H-cell, the measured difference between N_2_O and N_2_ selectivity can become smaller than that measured in flow-cell, while it does not overturn the dominance of N_2_O selectivity. Note that the pH effects in the range from 0.5 to 14 were also examined on Pd. Still, the N_2_O selectivity is higher with respect to the N_2_ production. As the NO partial pressure (concentration) changes from 100 to 25%, the N_2_ selectivity is still not desired compared to N_2_O production. If the electrode with higher overpotentials (<−0.3 V vs. RHE), it was demonstrated both N_2_ and N_2_O will lose dominance, which will be overturned by NH_3_ and H_2_ productions. At low overpotential, the production of N_2_O is more favorable, while NH_3_ or HER (hydrogen evolution reaction) are more selective at high overpotentials. In other words, the electrochemical denitrification with high N_2_ selectivity was not achieved yet for both low and high overpotentials. Copper (Cu) is exceptional as shown in Fig. 1b and both N_2_O and N_2_ are low in selectivity, which is even worse for the target of electrochemical denitrification.

In a word, all studied catalysts show higher selectivity to N_2_O than N_2_ at low overpotentials (from 0.8 to −0.3 V vs. RHE). There must be some key factors in eNORR, which are extremely fundamental for all studied transition metals (TMs) and determine the selectivity between N_2_ and N_2_O. Are there any candidates from TM catalysts for effective electrochemical denitrification? If inexistent, why aren’t TMs a great class of materials exhibiting high N_2_ selectivity? What are the chemical origins of low N_2_-selectivity over all TMs? What nature of active sites (surface reactivity) can be the efficient catalysts for electrochemical denitrification? Understanding the fundamental limitations in selectivity over TMs can guide us in catalyst design of eNORR. Although the potential and pH effects slightly tune the selectivity, N_2_ does never be the dominant product in diverse experimental conditions. We urgently need to understand the potential and pH effects on N_2_ selectivity in eNORR too. The exceptional Cu primarily produce NH_3_ rather than N_2_O and N_2_ in the whole potential window for both H-cell and flow-cell. Why is Cu a special catalyst in eNORR? Are there any alternative and feasible choices of eNORR, e.g., to upgrade NO_*x*_ to value-added chemicals (ammonia), rather than the conventional route of NO_*x*_ removal?

These questions above are quite essential to discover new catalysts or developing novel route for eNORR. To these ends, we have performed density functional theory (DFT) calculations to establish the reaction phase diagram, which was used to study the evolution of reaction mechanism over TMs. Finally, we have also developed a microkinetic model to analyze the kinetics of exceptional eNORR on copper to answer the questions above.

## Results

### A general model for activity trend

To understand the higher N_2_O selectivity with respect to N_2_ production, we need to study the mechanism of N-N bond formation and its evolution from most reactive catalysts (Fe) to medium ones (Co, Ni, Cu, Pt, and Pd), finally to the least reactive one (Ag). In general, there are two ways of N-N coupling for N_2_ and N_2_O productions. The first one is that an adsorbed nitrogenous intermediates, such as N*, NO* and NOH*, coupled with a NO molecule, namely Eley-Rideal-like (ER) mechanism^8,20^. The second one is the Langmuir-Hinshelwood (LH) mechanism^20–22^ that is the combination of two adsorbed N-containing intermediates. The N-N coupling is a (thermo)chemical step without the direct involvement of proton and electron, which means that the energetics can be minimally affected by electrode potential. Therefore, we calculated the intrinsic barriers of nine possible ways of N-N coupling on multiple metals (Supplementary Figs. 1–6) to exclude some unlikely reaction pathways. For TMs (Fe, Ni, and Pd) with strong nitrogen binding, the ER mechanism (Supplementary Fig. 6a–c) are much more favorable than LH mechanism (Supplementary Fig. 6d–i). For TMs with weak reactivity, e.g., Cu and Ag, three ways (LH mechanism) were also excluded due to higher barriers (Supplementary Fig. 6g–i), namely, two N* (or NOH*) coupling and their cross-couplings. An exception is the comparable barriers of ER and LH mechanisms on Ag. Previously, we have found that the formation of N-N bond proceeds through two NO* coupling on Ag^23^. Therefore, we only study the ER mechanisms and the two NO* coupling (LH) mechanism in the following analysis. In addition, considering some primary protonation steps, totally 34 elementary reactions (Supplementary Table 1) were employed to enumerate all possible pathways for N_2_ production.

To completely enumerate paths, all elementary steps were described with chemical vectors:

1 $${R}_{j}={\left({\nu }_{1j},{\nu }_{2j},\ldots,{\nu }_{{mj}}\right)}^{T}$$

where *v* represents the stoichiometric number of the involved substances (reactants, intermediates, and products) in the relevant steps, and subscript *m* represents the index of substances in the reaction, subscript *j* represents the index of elementary steps. The total reaction network can be described by the following matrix *S*.

2 $$S=\left[\begin{array}{ccc}{\nu }_{11} & \cdots & {\nu }_{1n}\\ \vdots & \ddots & \vdots \\ {\nu }_{m1} & \cdots & {\nu }_{{mn}}\end{array}\right]$$

where *n* refers to elementary steps in the reaction network. As a specific total reaction towards N_2_, N_2_O, NH_3_, and NH_2_OH productions can also be represented with a column vector,

3 $${R}_{T}={\left({\nu }_{1T},{\nu }_{2T},\ldots,{\nu }_{{mT}}\right)}^{T}$$

For a specific elementary step combination *c*_*i*_ = [*α*_1_, *α*_2_, …, *α*_k_], *α* ∈ [1, …, *n*], if it satisfies:

4 $${{{{{\rm{Rank}}}}}}\left(\left[\begin{array}{ccc}{\nu }_{{1\alpha }_{1}} & \cdots & {\nu }_{{m\alpha }_{1}}\\ \vdots & \ddots & \vdots \\ {\nu }_{{1\alpha }_{k}} & \cdots & {\nu }_{{m\alpha }_{k}}\end{array}\right]\right) \, \ge \, \ k$$

5 $${{{{{\rm{Rank}}}}}}\left(\left[\begin{array}{ccc}\begin{array}{c}{\nu }_{{1\alpha }_{1}}\\ \vdots \\ {\nu }_{{m\alpha }_{1}}\end{array} & \begin{array}{cc}\begin{array}{c}\cdots \\ \ddots \\ \ldots \end{array} & \begin{array}{c}{\nu }_{{1\alpha }_{k}}\\ \vdots \\ {\nu }_{{m\alpha }_{k}}\end{array}\end{array} & \left|\begin{array}{c}{\nu }_{{1T}_{j}}\\ \vdots \\ {\nu }_{{{mT}}_{j}}\end{array}\right.\end{array}\right]\right) \, < \, k+1$$

The coefficient matrix will have only one solution *p*_*i*_, *k* ∈ [2, …, *n*], which is the vector representative of pathway *i*. More details for this algorithm can be referred to in our previous work^24^. All enumerated reaction pathways for N_2_ production were summarized in Supplementary Table 2 and schematically shown in Supplementary Fig. 7.

**Fig. 2:**
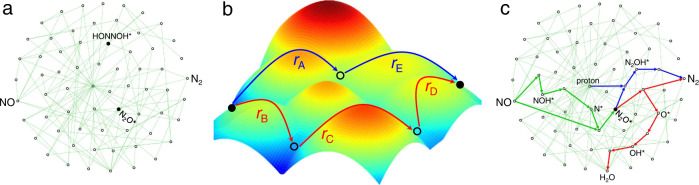
A scheme of determining the optimum pathways. **a** The full reaction network for N_2_ production (constructed from Supplementary Table 1), where HONNOH* and N_2_O* are the key intermediates of non-N_2_O* and dis-N_2_O*/p-N_2_O* paths (Supplementary Fig. 7), respectively. **b** The scheme of global energy optimization. **c** Two optimal paths towards N_2_ via the further conversion of as-produced N_2_O*, where the common route from NO to N_2_O* is shown in thick green lines. The blue and red arrows connect the p-N_2_O* and dis-N_2_O* paths, respectively.

To classify, the pathways of N_2_ production without N_2_O* involved are named non-N_2_O*. In case N_2_ was produced via dissociation or protonation of as-produced N_2_O*, abbreviated as dis-N_2_O* and p-N_2_O*, respectively. Among all enumerated pathways, we first performed the internal comparison for a given path to determine the most difficult step, *r*_max_. Then, the minimum *r*_max_ (ΔG_RPD_-limiting), identified from external comparison among all paths, can be used to describe the (quasi) activity, as shown in the following equation.

6 $$\Delta {{{{{{\rm{G}}}}}}}_{{{{{{\rm{RPD}}}}}}}-{{{{{\rm{limiting}}}}}}={{{\min }}}_{i}[{{{\max }}}_{j}(\Delta {{{{{{\rm{G}}}}}}}_{(i,j)})]$$

where *j* and *i* represents the elementary steps of a given path and all possible paths, respectively. As shown in Fig. 2, *r*_C_ is the key step, instead of *r*_B_, for the red path. The preference for blue and red paths is justified by the comparison between *r*_A_ and *r*_C_ steps. Hence, the ΔG_RPD_-limiting indicates a global optimization energy. As the *r*_max_ evolves from one metal to another, the adsorption energies of all intermediates were calculated and correlated with one or two descriptors. Then, we can employ descriptors to establish a reaction phase diagram (RPD) to distinguish the reaction mechanism/phase^24,25^. The comparison and identification of the ΔG_RPD_-limiting (and selectivity-determining) steps were illustrated in Supplementary Figs. 8, 9. In addition, we have also considered the reaction pathways (Supplementary Table 3) for ammonia (Supplementary Fig. 10) and hydroxylamine productions to establish a general model for various products in eNORR.

**Fig. 3:**
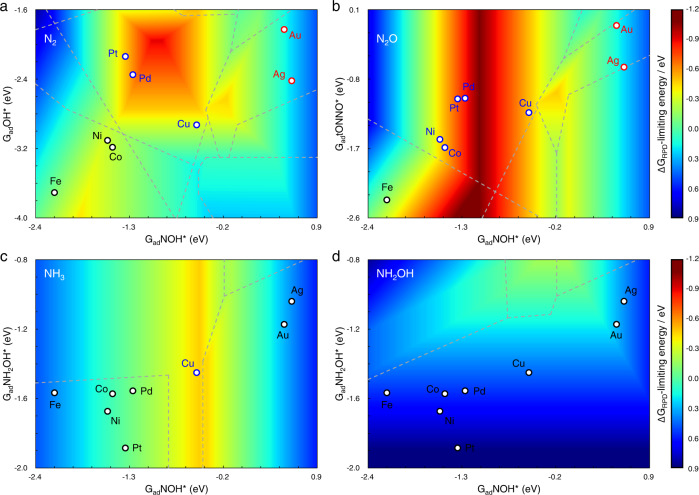
The two-dimensional reaction phase diagrams (2D-RPDs). **a**–**d** 2D-RPD for eNORR towards (**a**) N_2_, (**b**) N_2_O, (**c**) NH_3_, and (**d**) NH_2_OH at 0 V vs. RHE, where the windows with different mechanisms are divided by gray dotted lines (see details in Supplementary Table 4). ΔG_RPD_-limiting energies are shown with reference to the color bar on the right.

The two-dimensional reaction phase diagrams (2D-RPDs) for eNORR to different products are shown in Fig. 3. The 2D-RPD is the overlapping of the (quasi) activity maps of all possible reaction paths. Taking ammonia production as an example, the construction of 2D-RPD was illustrated in Supplementary Fig. 11. Figure 3a shows the (quasi) activity trend for N_2_ production, where all metals are not close to the optimum, indicating the low intrinsic activity of pure metals. Differently, Pd and Cu are nearly located at the optimum for N_2_O (Fig. 3b) and NH_3_ (Fig. 3c) production, respectively. For the three products, the similarity is that the favorable pathways, separated into multiple regions by gray dotted lines, are diverse on different metals (Supplementary Table 4). However, NH_2_OH production was found with the same mechanism over all studied metals (Supplementary Table 4), while the relatively strong chemical adsorption of NH_2_OH* limits its desorption (Fig. 3d), leading to low intrinsic activity towards hydroxylamine. It is quite consistent with the reported experiments^17^ that hydroxylamine is always a minor product.

**Fig. 4:**
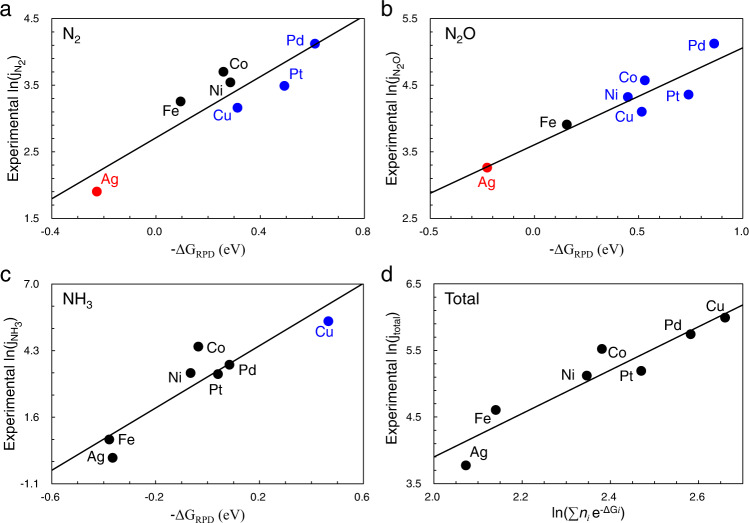
The comparison between theoretical descriptor (−ΔG_RPD_) and experimental activity^17^ (ln(*j*_*i*_)) at 0 V vs. RHE. **a**–**c** The comparison between computational ΔG_RPD_-limiting energy and partial current density of (**a**) N_2_, (**b**) N_2_O, (**c**) NH_3_. **d** The total current density compared against the theoretical activity descriptor. The colored points correspond to the diverse mechanisms of different metals (Fig. 3).

Overall, the ΔG_RPD_-limiting energies obtained by Fig. 3 can be used to estimate the activity trends of different products. Note that the number of electrons transferred towards different products (*n*_*i*_) affects the total current density (*j*_total_), so that an integrated descriptor ${{{{{\rm{ln}}}}}}\left(\sum {n}_{i}{e}^{{-\varDelta G}_{i}}\right)$ was applied for the estimation of total activity. Figure 4 shows the comparisons between theoretical and experimental activity, where the colored points correspond to the different mechanisms in Fig. 3. The theoretical activities give basic agreement with experimental trends, verifying the mechanisms obtained by 2D-RPD analysis. As the ΔG_RPD_-limiting energies are close, some kinetic factors can influence the absolute activity. For instance, there are some deviations from Co, Ni, and Cu for the activity trends of N_2_ and N_2_O productions (Fig. 4a, b). It can be attributed to the higher activity (larger current density) for ammonia production on Cu, resulting in relatively fewer NO contributions to N_2_ and N_2_O productions, compared to Co and Ni. In the following, we will analyze the evolution of reaction mechanisms for N_2_ and N_2_O production over all TMs to understand the fundamental limitations in N_2_ selectivity. For the comparison between N_2_ and N_2_O productions in one-dimensional reaction phase diagram, we used abnormal scaling relations for more accurate energetic descriptions. More details are shown in Supplementary Fig. 12. Besides, the effects of potential and pH on N_2_ selectivity were studied on Pd, as it is the most active catalyst for electrochemical denitrification (Fig. 3a). The low N_2_ and N_2_O activity on copper is discussed at the end in comparison with the primary ammonia production.

### Fundamental limitation in N_2_ selectivity for all TMs

**Fig. 5:**
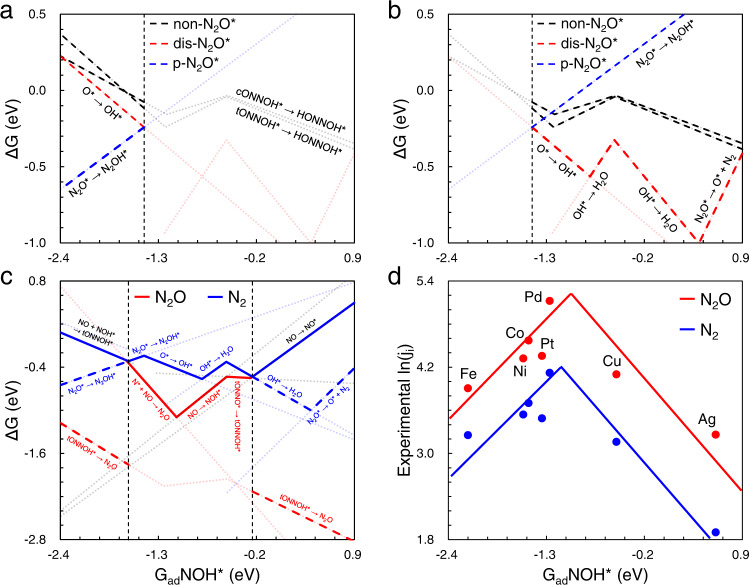
The one-dimensional reaction phase diagrams (1D-RPDs) and activity trend for N_2_ and N_2_O productions at 0 V vs. RHE. **a**, **b** The path preference of (**a**) p-N_2_O* (blue) on strongly reactive TMs (G_ad_NOH* <−1.46 eV) and (**b**) dis-N_2_O* (red) over others with G_ad_NOH* from −1.46 to 0.9 eV, compared to non-N_2_O* path (black), where the selective key steps are marked explicitly. **c** The selectivity analysis for N_2_ and N_2_O productions are colored in blue and red, respectively, where the ΔG_RPD_-limiting steps and key steps for product selection are shown in solid and dashed lines, respectively. **d** The measured activity trends (fitted blue and red lines) for N_2_ and N_2_O productions^17^.

According to Fig. 5a, b, N_2_ is produced via N_2_O* over all studied metals. Because the formation of HONNOH* (black dashed lines), a necessary step for non-N_2_O* paths, is more difficult than N_2_O* protonation (blue dashed line) in left window and the key steps of N_2_O* dissociative mechanism (red dashed lines) in right window. More details for the evolution of favorable N-N coupling mechanism are shown in Supplementary Figs. 13, 14. Meanwhile, the mechanisms of N_2_O production are explicitly shown in Supplementary Fig. 15. For the metals with strong adsorption (G_ad_NOH* <−1.46 eV), the coupling of NOH* and NO is more favorable than the other ways of N-N bond formation (Supplementary Fig. 13a–c). For these metals, such as Fe, Co and Ni, the activity is energetically limited by the reactions of NOH* coupled with NO and N_2_O* protonation (Supplementary Fig. 14a). The ΔG_RPD_-limiting steps of dis-N_2_O* path on other metals with weaker reactivity are the two-step protonation of O* and NO adsorption (Supplementary Fig. 14b). For the moderately reactive metals like Pd and Cu, the N-N bond is most likely formed via the combination between N* and NO (Supplementary Fig. 13d–f). As discussed in Supplementary Fig. 13g–i, the ER mechanism of NO* coupled with NO is preferable on metals with the weakest adsorption (Au and Ag).

Figure 5c shows the key steps for product selectivity in three reaction phases. In general, over the metals in the left window, N_2_ and N_2_O production share the same ΔG_RPD_-limiting step: NOH* coupled with NO to tONNOH*. In other words, the selectivity between N_2_ and N_2_O production over Fe is determined by the secondary energetic determining steps, as shown in Supplementary Table 5. The follow-up dehydration of tONNOH* on Fe directly releases N_2_O (tONNOH* → N_2_O, ΔG = −1.34 eV). Compared to the N_2_O release, N_2_O* protonation (N_2_O* → N_2_OH*, ΔG = −0.55 eV) is more difficult due to the (relatively) weak adsorption of N_2_OH*. The adsorption of N_2_OH* becomes weaker and weaker from left to right in 1D-RPD, giving rise to the exclusion of p-N_2_O paths on TMs with relatively weak reactivity. In the middle window, the ΔG_RPD_-limiting energies for N_2_ (blue solid lines) are higher than that of N_2_O (red solid lines). It indicates the lower selectivity of N_2_ than N_2_O production. Although the metals in the right window have equal ΔG_RPD_-limiting energies for the two products, the selectivity-determining steps, i.e., N_2_O* dissociation and OH* protonation, are much more difficult than N_2_O release. For instance, N_2_O* dissociation (N_2_O* → O* + N_2_, ΔG = −0.78 eV) on Ag is more difficult than N_2_O release (tONNOH* → N_2_O, ΔG = −2.64 eV). Supplementary Table 5 shows more details of energetic comparison on Ag. Hence, the reaction phase diagram explains well the lower N_2_ selectivity compared to N_2_O production over all studied TMs.

In addition, the dis-N_2_O* path is more preferable compared to p-N_2_O* on Pd for N_2_ production, as shown in Fig. 5c. However, the N_2_O production is the most favorable path with respect to both p-N_2_O* and dis-N_2_O* paths. In other words, we need to understand the energetic difference for the three limiting steps, namely, N* + NO → N_2_O* (−0.86 eV), O* → OH* (−0.34 eV) and N_2_O* → N_2_OH* (−0.15 eV). It can be rationalized well by the projected density of states, as shown in Supplementary Fig. 16. First, as the NO molecule has unpaired electron at Fermi level, it can be strongly interacting with the N*−2p states, resulting in a very stable N-N bond with the most stabilization of electronic states (Supplementary Fig. 16a). In contrast, the stabilization of N_2_O* by protonation is very limited since the N_2_O* has exhibited very stable electronic states (Supplementary Fig. 16b). In addition, as O* has some unsaturated 2p states around the Fermi level (Supplementary Fig. 16c), but lower intensity compared to NO, whose stabilization by protonation is between the two cases above.

The experimental activity trend (Fig. 5d) for N_2_ and N_2_O production over seven studied TMs indeed confirms our theoretical analysis above. The almost identical trends for the two products on all TMs greatly support our explanations that N_2_ is produced by N_2_O* further conversion. Besides, the lower N_2_ activity than N_2_O in the whole reactivity window shows an excellent agreement with theoretical analysis of selectivity. Several previous works have also found similar phenomena that the N_2_O production is dominant compared to N_2_ production^8,11^. In short, we identified two chemical origins for the low N_2_ selectivity. The first is that all TMs consistently bind N_2_OH* (relatively) too weakly. This inhibits the activity of N_2_O* protonation relative to its desorption. The second reason is that the scaling relation between O* and OH* on TMs limits their conversion or N_2_O* dissociation. This poisons the active sites for strongly reactive TMs. Meanwhile, it makes N-O bond breaking of N_2_O* difficult on metals with weak oxygen adsorption, resulting in a lower activity of N_2_ than N_2_O. The two fundamental factors synergistically result in the low N_2_ selectivity on TMs.

### Potential and pH effects on N_2_ selectivity

We now turn to another two influence factors, namely, electrode potential and electrolyte pH. The potential effects on the electrochemical process can be estimated by computational hydrogen electrode (CHE) model^26^. For thermochemical steps, the potential effect can be estimated through field effect on intermediates and transition states^27^, because an electric field exists at electrochemical interface. The intermediates can interact with the electric field due to their dipole moment and/or polarizability. The interaction will change their chemical potential and thus the corresponding reaction energies and barriers. Although it is difficult to directly measure the exact magnitude of the field, a reasonable approximation of linear correlation between electric field and absolute potential was reported previously^28–30^. Moreover, recent studies showed that the pH dependences can be explained well by field effect^28,31,32^. Thereby, the modeling of pH effects with an electric field (Eq. 13), as well as potential effect, are conducted in this section.

**Fig. 6:**
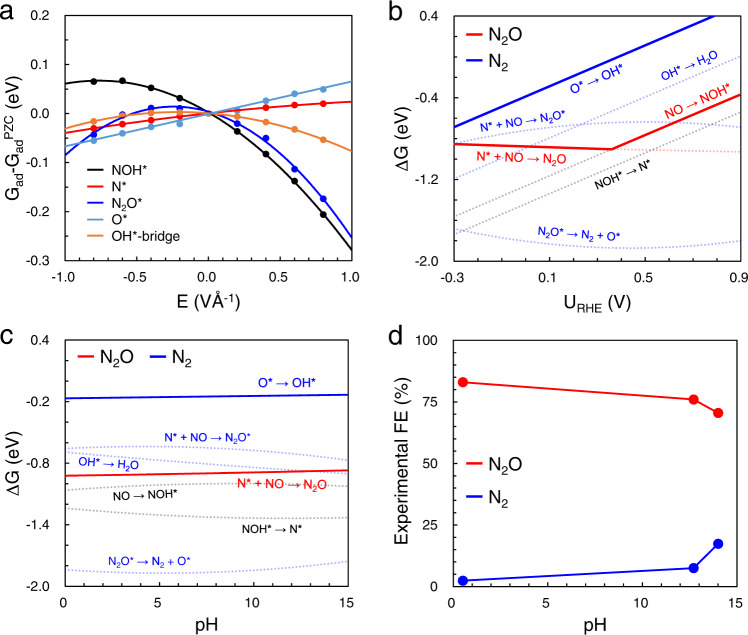
Potential and pH effects on product selectivity. **a** Field effects on intermediates involved in N_2_ and N_2_O production on Pd. The fitted values of *μ* (e Å) and *α* (e Å^2^ V^−1^) are shown in Supplementary Table 6. **b**, **c** The (**b**) potential-dependent and (**c**) pH-dependent (0.2 V vs. RHE) reaction free energies of steps in the optimum paths towards N_2_ and N_2_O on Pd. The blue and red lines refer to steps for N_2_ and N_2_O production, respectively, where the limiting steps are marked in bold. Note that the common steps (NO → NOH* → N*) for the two products are shown in black. The OH* is adsorbed at the bridge site, while the OH* at the top and hollow sites are shown in Supplementary Fig. 17. **d** The experimental FE of N_2_ and N_2_O on Pd, at 0.2 V vs. RHE^17^.

As mentioned above, the general order of major cathode products is dual-N products (N_2_O/N_2_), single-N products (NH_3_/NH_2_OH), and HER product (H_2_), from positive potentials to negative ones. Pd has been theoretically and experimentally identified as the most active metal for dual-N products (Figs. 3a, b, 5d). Herein, we only focus on Pd and the low overpotentials (>−0.3 V vs RHE) to study potential and pH effects on N_2_ selectivity. According to intrinsic dipole moment (*μ*) and polarizability (*α*) (Supplementary Table 6), the response to the field of various intermediates relevant to N_2_ and N_2_O productions was firstly calculated, as shown in Fig. 6a. G_ad_^PZC^ refers to the adsorption-free energy at the potential of zero charge (PZC), corresponding to the energy calculated without applied field. Then, the reaction energies of N-N formation and N-O scission are corrected to different potentials, as shown in Fig. 6b. The insensitivity of N* to field results in the N*-NO coupling in N_2_O production intact at potential <0.4 V vs. RHE. As the electrochemical steps are much more sensitive to potential than thermochemical ones, the O* → OH* (limiting step for N_2_ production) is beneficial and faster by potential effects. However, the ΔG_RPD_-limiting energies of N_2_ production is still much larger than N_2_O production. The difficult protonation of O* causes the poison of active sites. In a word, the potential effects can slightly promote N_2_ production, consistent with experiments, but not enough to exceed N_2_O.

At constant potential (RHE scale), an electrode at pH 12 have an absolute potential about 0.7 V lower than the one at pH 0. The difference in absolute potential leads to that the electrode at pH 12 exposed to an electric field nearly 1 V Å^−1^ stronger than the case at pH 0. In such electric field, the deviation of adsorbate binding energies could be an important contribution to the pH effect. Thus, pH dependence is proposed to originate from dependence on electric field, as clearly described in “Modeling pH effect with an electric field” paragraph. Figure 6c shows the pH effect on N_2_ and N_2_O production at 0.2 V vs. RHE, where solid lines refer to the ΔG_RPD_-limiting steps. The field insensitivity of N* results in the activity towards N_2_O intact at varying pH (red solid line). Besides, the dipole moments of OH* (bridge site) and O* species are very small, giving rise to the almost constant ΔG_RPD_-limiting energies (blue solid line: O* → OH*) for N_2_ production, which are much larger than that for N_2_O production in the whole pH window. It suggests the lower N_2_ selectivity than N_2_O at all pH, consistent with the experiments shown in Fig. 6d. In addition, the OH*-Pd local structure has negative dipole moment and small polarizability response to electric field at hollow site, since it exhibits (almost) vertical adsorption structure. While the dipole moment is positive at top site due to the almost horizontal O-H bond (Supplementary Fig. 17). This means, the negative field can stabilize the OH* on top site, while it destabilizes the ones at hollow site, as shown in Supplementary Fig. 17a. Supplementary Fig. 17b shows that the O* → OH* is more favorable at hollow site in acidic condition. Instead, the O* → OH* at top site can enhance the activity in alkaline condition. Moreover, the OH* coverage is relatively lower at acidic condition. It should be dominant by the more favorable OH* at bridge and hollow sites. At alkaline condition, the OH* coverage should be higher and with more OH* at top site. Therefore, the N_2_ production can be further enhanced at alkaline condition from OH* at top sites (Supplementary Fig. 17b).

Certainly, the energetic difference from the OH* adsorption sites is small. Another important influence factor should be that the N_2_O* adsorption becomes more stable at alkaline condition (Fig. 6a). In other words, the local pressure/concentration of N_2_O can be higher at alkaline condition. As the lifetime of N_2_O at electrochemical interface becomes longer, it will enhance the second conversion of as-produced N_2_O. As shown in Fig. 1, the Faradaic efficiency of N_2_ and N_2_O production can fluctuate by ~15% at varying NO partial pressures. We believe the local pressure or concentration of N_2_O can also change the Faradaic efficiency with the comparable magnitude. In addition, surface engineering of electrode can be another possibility to enhance the activity or selectivity. The H*-covered Pd and new Pd-based materials should be possible to improve N_2_ selectivity. However, it is very hard to form a high H* coverage because the NO* adsorption is always stronger than H* (Supplementary Fig. 18). Although it is very unfavorable to form PdH above 0 V vs. RHE, it might be possible at very negative potentials. Hence, we calculated the activity and selectivity of eNORR to N_2_ production on H*-covered Pd and PdH. Details for the models are shown in Supplementary Figs. 19, 20. As shown in Supplementary Fig. 21a, it was found the activity of N_2_ production was lower compared to Pd. Besides, the N_2_ selectivity was lower than N_2_O over all studied materials (Supplementary Fig. 21b). It indicates that H*-modified Pd cannot realize a higher N_2_ activity and selectivity. Moreover, we have calculated the activity of N_2_ and N_2_O production over six Pd-based alloys (Supplementary Fig. 22). However, it fails to find a candidate with higher N_2_ selectivity against N_2_O production (Supplementary Fig. 23).

### An exceptional selectivity over copper

Cu is exceptional to all other TMs and favors ammonia production with very low selectivity, not only for N_2_, but also N_2_O production. As shown in Supplementary Fig. 24, ammonia production indeed has comparable energies in limiting steps with N_2_O production on copper. For strongly reactive catalysts, the N_2_O production is much favorable with respect to ammonia production. The previous work has also found that NO protonation has a larger barrier than its adsorption over weakly reactive catalysts, for instance, 0.4 eV on Ag^23^. This indicates most TMs should be N_2_O-selective and Cu is exceptional in selectivity. Indeed, the experimental selectivity (Supplementary Fig. 25) is consistent with our thermodynamic analysis.

**Fig. 7:**
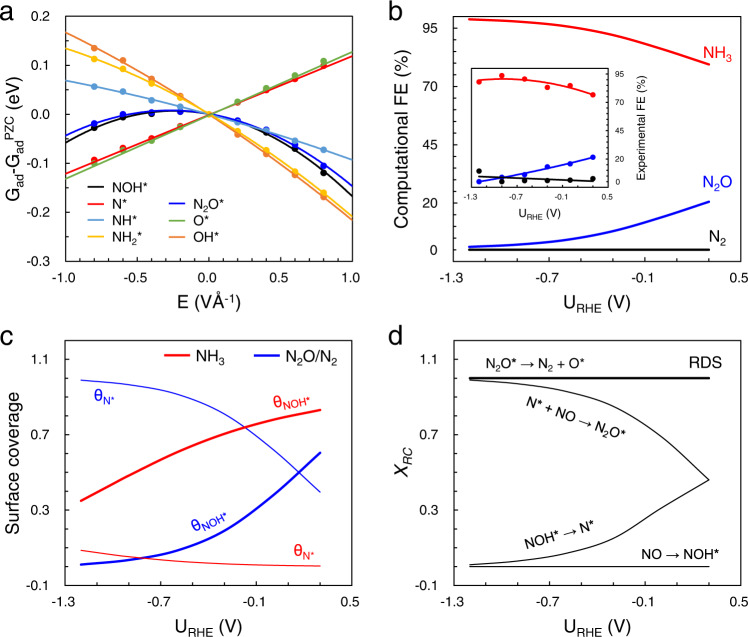
The results of microkinetic modeling on Cu. **a** Field effect on the adsorption energies of relevant intermediates in eNORR on Cu. **b** The computational and experimental (insert)^19^ FE of different products for eNORR on Cu. **c** The surface coverage of key intermediates in the formation of NH_3_ (red lines) and dual-N products (blue lines). **d** Degree of rate control (DRC) of different elementary steps (*X*_*RC*_) for N_2_ production, where the rate-determining step (RDS) is shown in a thick line.

In addition, we built a microkinetic model to study the potential and electric effects. The barriers for N_2_O and NH_3_ production were explicitly calculated in our previous work^19^. For proton-electron coupled transfer reactions, a monolayer of water containing a hydronium (H_3_O^+^) was explicitly added on the Cu(111) surface. The electrochemical barriers were extrapolated to a given electrode potential with the charge-extrapolation scheme based on a capacitor model^33,34^. More computational details of potential-dependent barriers are given in Eqs. 14 and 15. Towards N_2_, the dissociative barrier of N_2_O* to N_2_ + O* (Supplementary Fig. 4) and the two-step protonation barriers of O* to H_2_O (Supplementary Fig. 26) were also calculated in this work. The pH effect can be diverse and complex in kinetics. A major contribution from electric field (Fig. 7a) was considered in our model.

To compare with the previous experiments^19^, the microkinetic modelings were performed at pH 7.0, employing the corrected energetics listed in Supplementary Table 7. Following Arrhenius equation, the rate constants were computed at different potentials. According to the steady-state approximation^35^, the surface coverage (θ) of adsorbates for NH_3_ and N_2_/N_2_O production was solved at varying potentials, respectively. More details can be found in “Microkinetic modeling” paragraph. Correspondingly, the intrinsic activity for NH_3_ or N_2_/N_2_O can be theoretically estimated. In the wide potential region from 0.3 to −1.2 V, the Faradaic efficiency of NH_3_ is much higher than N_2_ and N_2_O, which shows a great agreement with experimental trends (Fig. 7b, insert).

The production of NH_3_ and N_2_/N_2_O have similar apparent activation barriers at all studied potentials, as shown in Supplementary Fig. 27a. It suggests that NH_3_ production has no obvious superiority at kinetic barriers. The high selectivity of NH_3_ should be attributed to preferable (total) thermodynamics, as reflected from the surface coverage of key intermediates. The degree of rate control (DRC) for elementary steps (*X*_*RC*_) was analyzed, which reflects the influence of a given step on the overall reaction^36,37^. The rate-determining step (RDS) is defined as the step with the largest *X*_*RC*_. As the RDS for NH_3_ production (Supplementary Fig. 27b), the electrochemical dehydration of NOH* to N*, is also a necessary step for N_2_/N_2_O production. The conversion of N* is the key step for the selectivity towards different products, the rate of which influences the surface coverage of NOH* (θ_NOH*_) at steady-state. For ammonia production, the continuous protonation of N* is faster, giving rise to low θ_N*_ and thus high θ_NOH*_ (Fig. 7c). However, the sluggish coupling of N* and NO makes the active site mainly covered by N* for N_2_/N_2_O production, leading to low θ_NOH*_. A lower θ_NOH*_ indicates a slower rate of NO conversion. This indicates that the exceptionally high ammonia selectivity on Cu can be attributed to facile N* protonation. Another influence factor, partial pressure of NO, was tested from 0.001 to 1 at 0.3 V vs. RHE. The results (Supplementary Fig. 27c) did not show the reversal of selectivity, although a high NO pressure facilitates the N-N coupling reaction. In addition, the selectivity between N_2_ and N_2_O can be understood by DRC analysis. As shown in Fig. 7d, the RDS for N_2_ is always the dissociation of N_2_O*, which is more difficult than the barrierless desorption of N_2_O*. The slower dissociation of N_2_O* results in a lower N_2_ selectivity over copper.

Therefore, we would propose to make efforts in the following four directions. (1) To design the catalysts with distinctly strong N_2_OH* adsorption (breaking scaling relation) or discover an alternative class of materials, instead of TMs, this is a possible solution for enhancing the N_2_ selectivity with respect to N_2_O production. (2) Although the choice/engineering of electrodes is demonstrated to be very difficult to enhance N_2_ selectivity, the design of the reactor can be considered as another factor. For instance, a high N_2_ selectivity with respect to N_2_O production was achieved^11–13^, where the electrode has a much larger surface area and the NO flow rate is low instead. Towards high N_2_ selectivity, it should be a feasible route to construct a cascade reactor via the combination of NO-to-N_2_O and N_2_O-to-N_2_, as the direct N_2_O electroreduction to N_2_ is highly efficient^17^. (3) To enhance the efficiency of thermochemical steps, it can be achieved by reducing the accessibility of proton to suppress electrochemical steps, for instance, by using non-aqueous proton donor^38–41^. (4) At last, we can also steer from electrochemical denitrification to ammonia synthesis as the latter is a fundamentally more feasible route based on Cu-based catalysts. The eNORR route can also serve as an electrochemical platform for amination.

## Discussion

As discussed above, the low N_2_ selectivity in eNORR originates from two aspects. On the one hand, the surface reactivity of all TMs is very disadvantageous to N_2_ production from the energetic point of view. Firstly, TM surfaces consistently bind N_2_OH* too weakly, inhibiting the activity of N_2_O* protonation relative to its desorption. Secondly, the scaling relation between O* and OH* makes the active sites on strong reactive TM surface poisoned by O*/OH* or causes N_2_O* dissociation very difficult on metals with weak adsorption. The two natures synergistically make all TMs more N_2_O-selective. Note that the key factor for high NH_3_ selectivity on Cu is the facile N* protonation. On the other hand, the optimization of experimental conditions, such as potential, pH and NO pressure, can slightly promote N_2_ selectivity, but not enough to exceed N_2_O or NH_3_, as observed experimentally. The comparisons between experimental and theoretical results indicate the activity trend for various products can be well described by the reaction phase diagram. The insights for low N_2_ selectivity in eNORR can be a guideline for the future design of catalysts. More importantly, the exceptional activity of ammonia production provided a feasible route to build up a reverse artificial nitrogen cycle, which can play a critical role for decentralized ammonia synthesis with sustainable electricity.

## Methods

### Computational details

DFT calculations were performed using Vienna Ab initio Simulation Package (VASP)^42–44^. All calculations were conducted at the level of generalized gradient approximation (GGA)^45^ with the revised Perdew-Burke-Ernzerhof^46^ functional. The projected augmented wave (PAW)^47,48^ method was used. Spin-polarized calculations were made to Fe, Co, and Ni and the rest metals are spin restricted. A kinetic energy cutoff of 400 eV was used in this work. Structural optimizations were performed with the residual force and electronic energy differences smaller than −0.05 eV/Å and 10^−5^ eV, respectively. The van der Waals corrections^49,50^ were considered by DFT-D3 method. To calculate the adsorption energies for different adsorbates, the energies of gas-phase N_2_, H_2_, and NO were used as references. For free energy correction^51^, in terms of zero-point energy and entropic (S) contribution, electronic energies were corrected at the experimental temperature (*T* = 298 K), where the vibrational entropy for adsorbed intermediates while translational, rotational, and vibrational motions for gases were considered, respectively. To locate transition states, the climbing image nudged elastic band (CI-NEB) scheme^52^ and dimer method^53,54^ were employed.

For atomic models, a close-packed bcc(110) surface was used to model the flat surface of Fe metal, while fcc(111) surfaces were used for Co, Ni, Pt, Pd, Cu, Au, and Ag. Four-layer 3 × 8 supercell was constructed for bcc(110) surface of Fe, and 4 × 6 slabs were established for other metals. The adsorbates were permitted to relax with the two top-layer metal atoms, while the two bottom layers were fixed. Besides, a vacuum region of ~15 Å was introduced along the z-direction to avoid interactions between images. All slabs were optimized by a Monkhorst-Pack k-point of 2 × 2 × 1. The scheme of Methfessel-Paxton^55^ (*N* = 1) was used for the smearing width of 0.2 eV. Moreover, the solvation (Supplementary Tables 8 and 9) and field effect on adsorption energy of intermediates were considered in DFT calculations. Electric fields were applied using a saw-tooth potential corresponding to fields between −0.8 and 0.8 VÅ^−1^. All adsorbates were relaxed at each applied field, which was used to predict their response to field.

### Modeling response of adsorbates to electric field

The response of adsorbed intermediates to the field can be measured by the fitted second-order polynomial:

7 $${G}_{{{{{{\rm{ad}}}}}}}={G}_{{{{{{\rm{ad}}}}}}}^{{{{{{\rm{PZC}}}}}}}+\mu {{{{{\bf{E}}}}}}-\frac{\alpha }{2}{{{{{{\bf{E}}}}}}}^{2}$$

where **E** is the applied field and *G*_ad_^PZC^ refers to the adsorption-free energy at potential of zero charge (PZC), corresponding to the energy calculated with no applied field. Here, *μ* and *α* represent the intrinsic dipole moment and polarizability of adsorbates, respectively. By fitting calculated (*G*_ad_–*G*_ad_^PZC^) with field **E**, *μ* and *α* of each intermediates can be determined. Furthermore, based on a parallel-plate capacitor model, a linear correlation between electric field and absolute potential was approximated as following:

8 $${{{{{\bf{E}}}}}}=\frac{\sigma }{\varepsilon {\varepsilon }_{0}}=\frac{{C}_{{{{{{\rm{H}}}}}}}\left({U}_{{{{{{\rm{SHE}}}}}}}-{U}_{{{{{{\rm{PZC}}}}}}}\right)}{\varepsilon {\varepsilon }_{0}}$$

where *σ* is the surface charge density, *ε* and *ε*_0_ are the dielectric constant of vacuum and water near interface, which were set to be 8.85 × 10^−12 ^F m^−1^ and 2 (unitless), respectively. *C*_H_ refers to Helmholtz capacitance (μF cm^−2^), which can vary with the surface and potential but ranged between 20 and 60 μF cm^−2^ ^28,56,57^. We used a constant *C*_H_ of 25 μF cm^−2^ across all surfaces and potential^28^. *U*_SHE_ is the electrode potential referenced to standard hydrogen electrode (SHE). *U*_PZC_ refers to the potential at PZC versus SHE. The experimental values of 0 and 0.09 V were used for *U*_PZC_ on Pd and Cu^58^.

### Modeling pH effect with an electric field

As described by Eq. 8, the electric field depends on the absolute potential of an electrode. It can be measured by using a standard hydrogen electrode (*U*_SHE_), which relates to RHE (reversible hydrogen electrode) by Eq. 9.

9 $${U}_{{{{{{\rm{SHE}}}}}}}={U}_{{{{{{\rm{RHE}}}}}}}-{k}_{B}T{{{{{\rm{ln}}}}}}\left(10\right)\times \left({{{{{\rm{pH}}}}}}\right)={U}_{{{{{{\rm{RHE}}}}}}}-0.059{{{{{\rm{pH}}}}}}$$

Thus, electric field dependencies manifest themselves as dependencies on pH when viewed on an RHE scale. In detail, according to Eqs. 7, 8, the effect of electrode potential on intermediates adsorption energy can be estimated by the following mathematical expression:

10 $${G}_{{{{{{\rm{ad}}}}}}}={G}_{{{{{{\rm{ad}}}}}}}^{{{{{{\rm{PZC}}}}}}}+\mu \frac{{C}_{{{{{{\rm{H}}}}}}}}{{\varepsilon \varepsilon }_{0}}\left({U}_{{{{{{\rm{SHE}}}}}}}-{U}_{{{{{{\rm{PZC}}}}}}}\right)-\frac{\alpha }{2}{\left(\frac{{C}_{{{{{{\rm{H}}}}}}}}{{\varepsilon \varepsilon }_{0}}\right)}^{2}{\left({U}_{{{{{{\rm{SHE}}}}}}}-{U}_{{{{{{\rm{PZC}}}}}}}\right)}^{2}$$

At a given RHE potential and varying pH, Eq. 10 has new expressions as Eqs. 11 and 12:

11 $${G}_{{ad}}^{{{{{{\rm{pH}}}}}}=0}={G}_{{{{{{\rm{ad}}}}}}}^{{{{{{\rm{PZC}}}}}}}+\mu \frac{{C}_{{{{{{\rm{H}}}}}}}}{{\varepsilon \varepsilon }_{0}}\left({U}_{{{{{{\rm{RHE}}}}}}}-{U}_{{{{{{\rm{PZC}}}}}}}\right)-\frac{\alpha }{2}{\left(\frac{{C}_{{{{{{\rm{H}}}}}}}}{{\varepsilon \varepsilon }_{0}}\right)}^{2}{\left({U}_{{{{{{\rm{RHE}}}}}}}-{U}_{{{{{{\rm{PZC}}}}}}}\right)}^{2}$$

12 $${G}_{{ad}}^{{{{{{\rm{pH}}}}}}}=	\,{G}_{{{{{{\rm{ad}}}}}}}^{{{{{{\rm{PZC}}}}}}}+\mu \frac{{C}_{{{{{{\rm{H}}}}}}}}{{\varepsilon \varepsilon }_{0}}\left({U}_{{{{{{\rm{RHE}}}}}}}-{U}_{{{{{{\rm{PZC}}}}}}}\right)-\frac{\alpha }{2}{\left(\frac{{C}_{{{{{{\rm{H}}}}}}}}{{\varepsilon \varepsilon }_{0}}\right)}^{2}{\left({U}_{{{{{{\rm{RHE}}}}}}}-{U}_{{{{{{\rm{PZC}}}}}}}\right)}^{2} \\ 	+\mu \frac{{C}_{{{{{{\rm{H}}}}}}}}{{\varepsilon \varepsilon }_{0}}\left(-0.059{{{{{\rm{pH}}}}}}\right)-\frac{\alpha }{2}{\left(\frac{{C}_{{{{{{\rm{H}}}}}}}}{{\varepsilon \varepsilon }_{0}}\right)}^{2}{\left(-0.059{{{{{\rm{pH}}}}}}\right)}^{2}$$

Here, we see that the adsorption energy of an adsorbate can be split into three contributions: the intrinsic binding strength of the catalysts described by ${G}_{{{{{{\rm{ad}}}}}}}^{{{{{{\rm{PZC}}}}}}}$, the RHE potential dependence, and pH dependence described by the *μ* and *α* terms. In short, the brief formula for modeling pH effect with an electric field is shown as Eq. 13.

13 $${G}_{{ad}}^{{{{{{\rm{pH}}}}}}}={G}_{{ad}}^{{{{{{\rm{pH}}}}}}=0}+\mu \frac{{C}_{{{{{{\rm{H}}}}}}}}{{\varepsilon \varepsilon }_{0}}\left(-0.059{{{{{\rm{pH}}}}}}\right)-\frac{\alpha }{2}{\left(\frac{{C}_{{{{{{\rm{H}}}}}}}}{{\varepsilon \varepsilon }_{0}}\right)}^{2}{\left(-0.059{{{{{\rm{pH}}}}}}\right)}^{2}$$

### Microkinetic modeling

The potential-dependent barriers (G_a_) of proton-electron coupled transfer reactions were calculated by “charge-extrapolation” method^33,34^. As proton source, a monolayer water containing a hydronium (H_3_O^+^) was placed on the surface. According to a capacitor model, the barriers can be extrapolated to a given electrode potential based on the work function variations, as described in the following equations:

14 $${G}_{a}\left({\varPhi }_{{IS}}\right)=	\,{G}_{{TS}}\left({\varPhi }_{{IS}}\right)-{G}_{{IS}}\left({\varPhi }_{{IS}}\right)={G}_{{TS}}\left({\varPhi }_{{TS}}\right)-{G}_{{IS}}\left({\varPhi }_{{IS}}\right) \\ 	+\frac{\left({q}_{{TS}}-{q}_{{IS}}\right)\left({\varPhi }_{{TS}}-{\varPhi }_{{IS}}\right)}{2}$$

15 $${G}_{a}\left({\varPhi }_{{TS}}\right)=	\,{G}_{{TS}}\left({\varPhi }_{{TS}}\right)-{G}_{{IS}}\left({\varPhi }_{{TS}}\right)={G}_{{TS}}\left({\varPhi }_{{TS}}\right)-{G}_{{IS}}\left({\varPhi }_{{IS}}\right)\\ 	-\frac{\left({q}_{{TS}}-{q}_{{IS}}\right)\left({\varPhi }_{{TS}}-{\varPhi }_{{IS}}\right)}{2}$$

where G_IS_(*Φ*_IS_) and G_TS_(*Φ*_TS_) are corresponding to the free energies of initial and transition states, respectively. *Φ* and *q* refer work function and charge change of water layer, respectively. Accordingly, the slope of G_a_ changing with potential (*Φ*), i.e. charge transfer coefficient *β*, was computed by taking Eqs. 14, 15 and approximated to be constant. A specific *Φ* corresponds to an electrode potential (vs. SHE) by following equation:

16 $${U}_{{SHE}}=\frac{\varPhi -{\varPhi }_{{SHE}}}{e}$$

where *Φ*_SHE_ was detected experimentally to be ~4.4 eV. By the scheme above, the potential-dependent G_a_ of electrochemical steps towards NH_3_ and N_2_O were calculated, as reported in our previous work^19^. Towards N_2_, the dissociative barrier of N_2_O* to N_2_ + O* and the two-step protonation barriers of O* to H_2_O were calculated in this work.

The microkinetic modeling of eNORR on Cu was based on the method by JianFu Chen and HaiFeng Wang, using the CATKINAS package^59^. To study the intrinsic activity for NH_3_ and dual-N products (N_2_/N_2_O), microkinetic simulations were conducted at two sites. The surface coverage (*θ*) of adsorbates was solved with the steady-state approximation. The rates for elementary reactions were calculated using the follow-up equation:

17 $${{{{{\rm{rate}}}}}}={k}_{f}\prod {\theta }_{{reac}}-{k}_{b}\prod {\theta }_{{prod}}$$

where *θ*_*reac*_ and *θ*_*prod*_ refer to the coverages of reactants and products, respectively. The reaction constants *k*_*f*_ and *k*_*b*_, respectively, reflect the degree of difficulty of forward and backward reactions, which was calculated by Arrhenius equation:

18 $$k=A{e}^{-\frac{{G}_{a}}{{k}_{B}T}}$$

where *A* (s^−1^), *G*_*a*_, *k*_*B*_ and *T* are reaction prefactor, activation free energy, Boltzmann constant and reaction temperature, respectively.

In this work, microkinetic modeling was performed at 298 K and an RHE potential region from 0.3 to −1.2 V. The partial pressure of NO was firstly tested from 0.001 to 1 at 0.3 V (Supplementary Fig. 27c) and finally set to be 0.1 atm due to the very low solubility of NO in aqueous solution. Besides, the partial pressure of products (N_2_, N_2_O, and NH_3_) was set at 0.01 atm due to the relatively low yield rate. All energetics employed for microkinetic modeling are listed in Supplementary Table 7.

The computational Faradaic efficiency for each product was calculated by the following equation:

19 $${{{{{\rm{FE}}}}}}\left(\%\right)=\frac{{n}_{i}{{TOF}}_{i}}{\sum {n}_{i}{{TOF}}_{i}}\times 100\%$$

where *n*_*i*_ refers to the total electron transfer number and TOF_*i*_ represents the turnover frequency obtained by microkinetic modeling for product *i*.

## Supplementary information

Supplementary Information

Description of Additional Supplementary Files

Supplementary Dataset 1

Supplementary Dataset 2

## Data Availability

The optimized structures of Pd-based alloys and PdH surfaces are provided in Supplementary dataset 1. Besides, all data that support the findings of this study are presented in the main text and Supplementary information, where the source data of Figs. 1, 3, 4, 6, and 7 are listed in Supplementary dataset 2. The additional datasets are available from the corresponding author upon reasonable request.

## Code availability

The VASP used for DFT calculations is available from the website https://www.vasp.at/, and the CATKINAS package used for microkinetic modeling is available from the website https://www.catkinas.com/. For both simulation techniques, input and output files are available from the corresponding author upon reasonable request.
