# Supplementary material for: Steering from electrochemical denitrification to ammonia synthesis
Source: Nat Commun. 2023 Jan 7;14:112. doi: 10.1038/s41467-023-35785-w (PMC9825404; doi:10.1038/s41467-023-35785-w)
Supplement: Supplementary file 1 — Supplementary Information [file 41467_2023_35785_MOESM1_ESM.pdf]

Supplementary information for

Steering from electrochemical denitrification to ammonia synthesis

Huan Li[1,2], Jun Long[1], Huijuan Jing[1,2], Jianping Xiao*[1,2]

[1] *State Key Laboratory of Catalysis, Dalian Institute of Chemical Physics, Dalian National Laboratory for Clean Energy, Chinese Academy of Sciences, Dalian 116023, P. R. China.*
[2] *University of Chinese Academy of Sciences, Beijing 100049, P. R. China.*

* Corresponding author: xiao@dicp.ac.cn

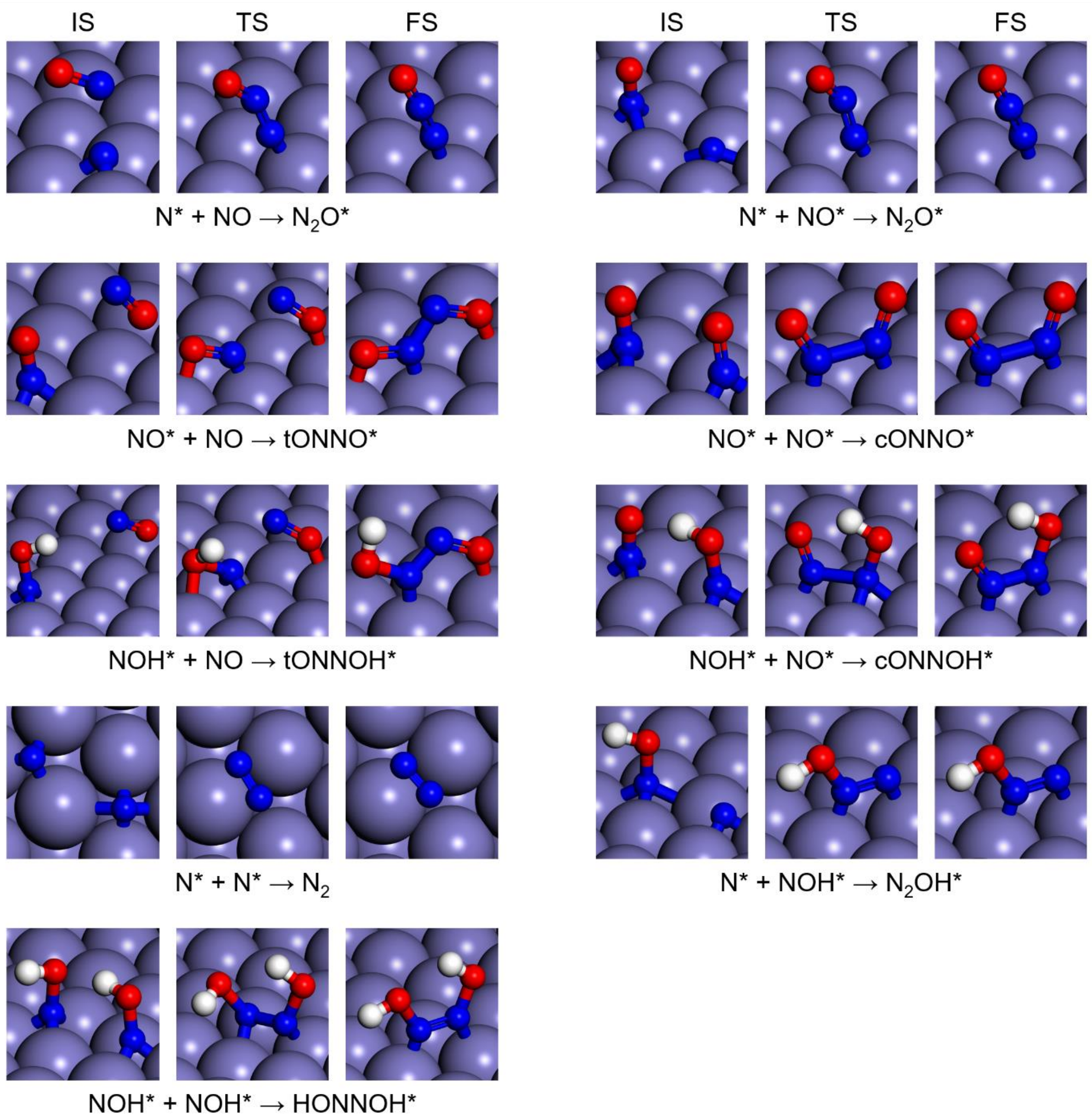

Supplementary Fig. 1 Calculated adsorption structures and transition states (TSs) of thermochemical N-N coupling steps on Fe(110) surface (c=cis, t=trans). Note that the cis-$(NO)_2$ dimer, namely cONNO*, is an unstable adsorbate on metals with strong reactivity, such as Fe, Co, Ni, Pt and Pd. Thus, the adsorption structures of cONNO* on those metal surfaces were optimized with constrained N atoms along x and y directions. This is consistent unless specified otherwise.

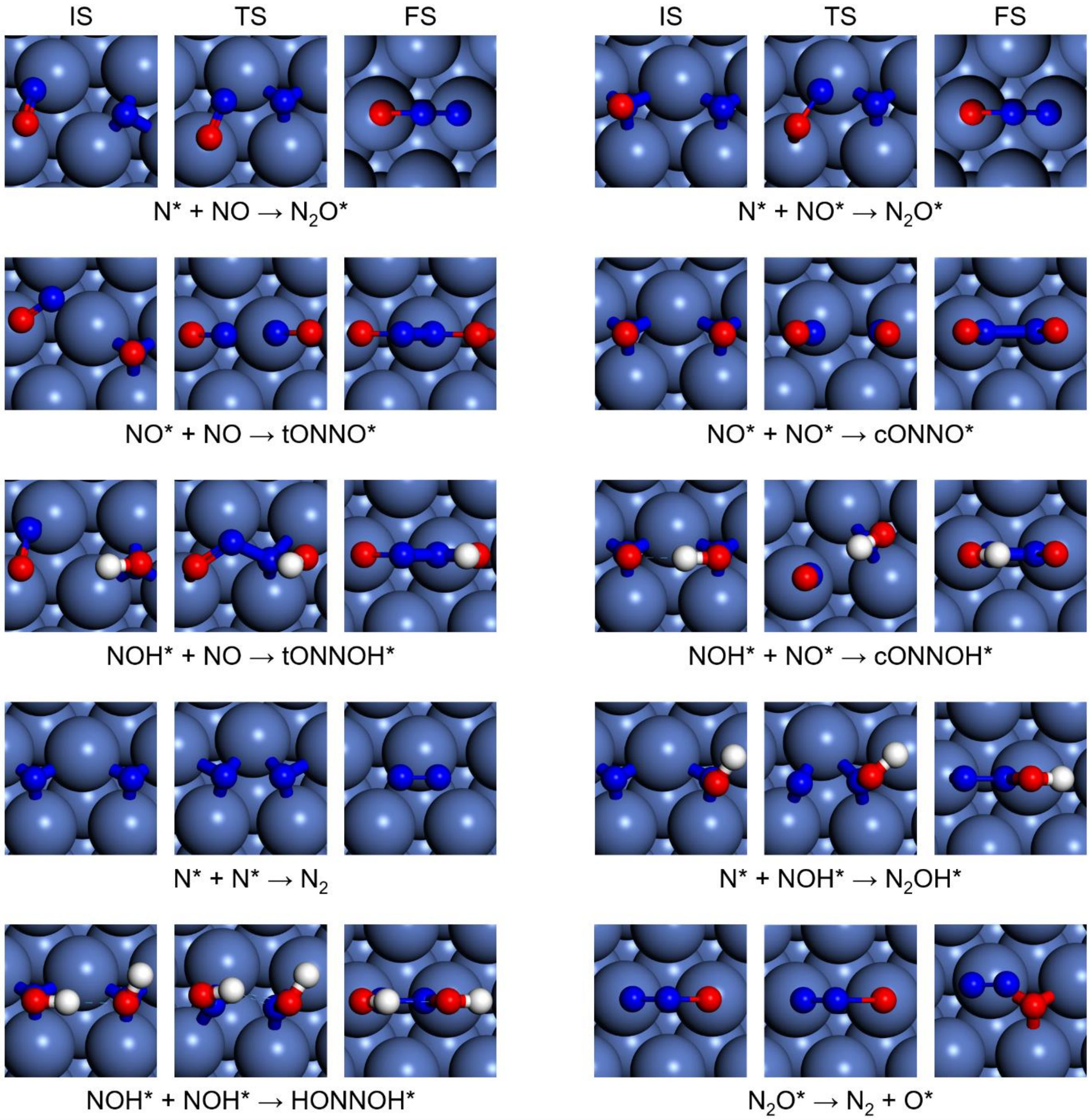

Supplementary Fig. 2 Calculated adsorption structures and the TSs of thermochemical N-N coupling and N-O breaking steps on Ni(111) surface (c=cis, t=trans).

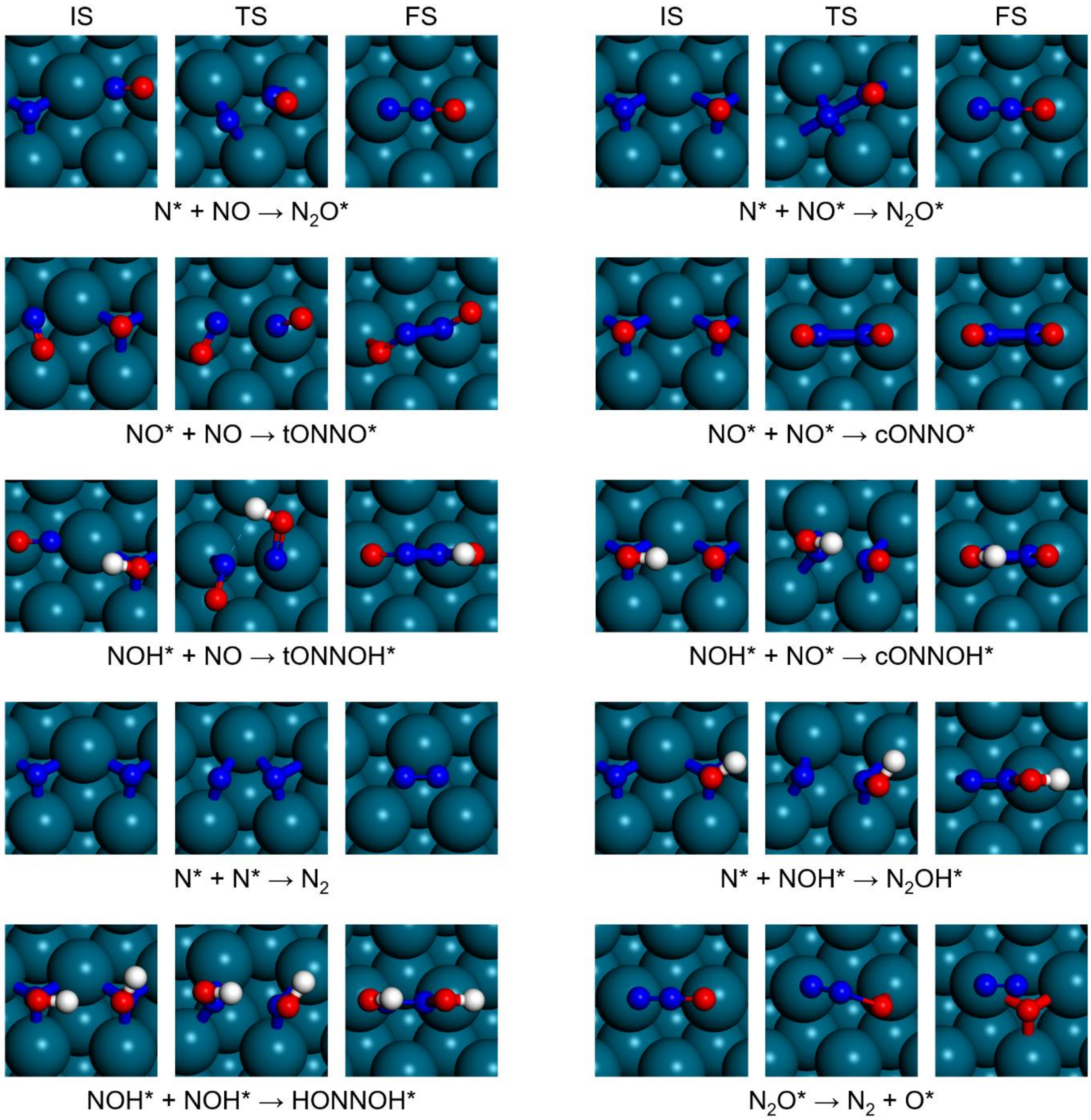

Supplementary Fig. 3 Calculated adsorption structures and the TSs of thermochemical N-N coupling and N-O breaking steps on Pd(111) surface (c=cis, t=trans).

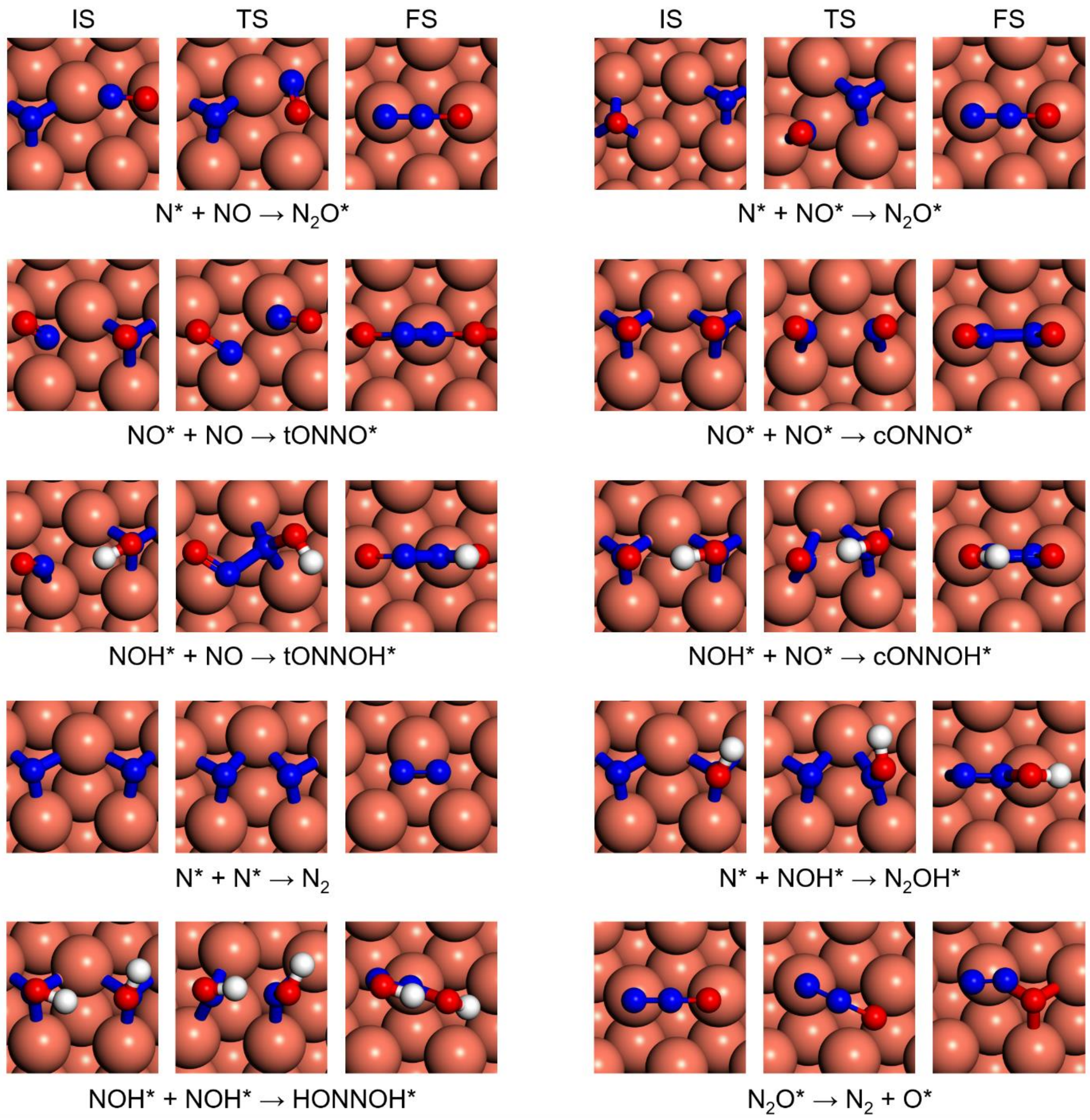

Supplementary Fig. 4 Calculated adsorption structures and the TSs of thermochemical N-N coupling and N-O breaking steps on Cu(111) surface (c=cis, t=trans).

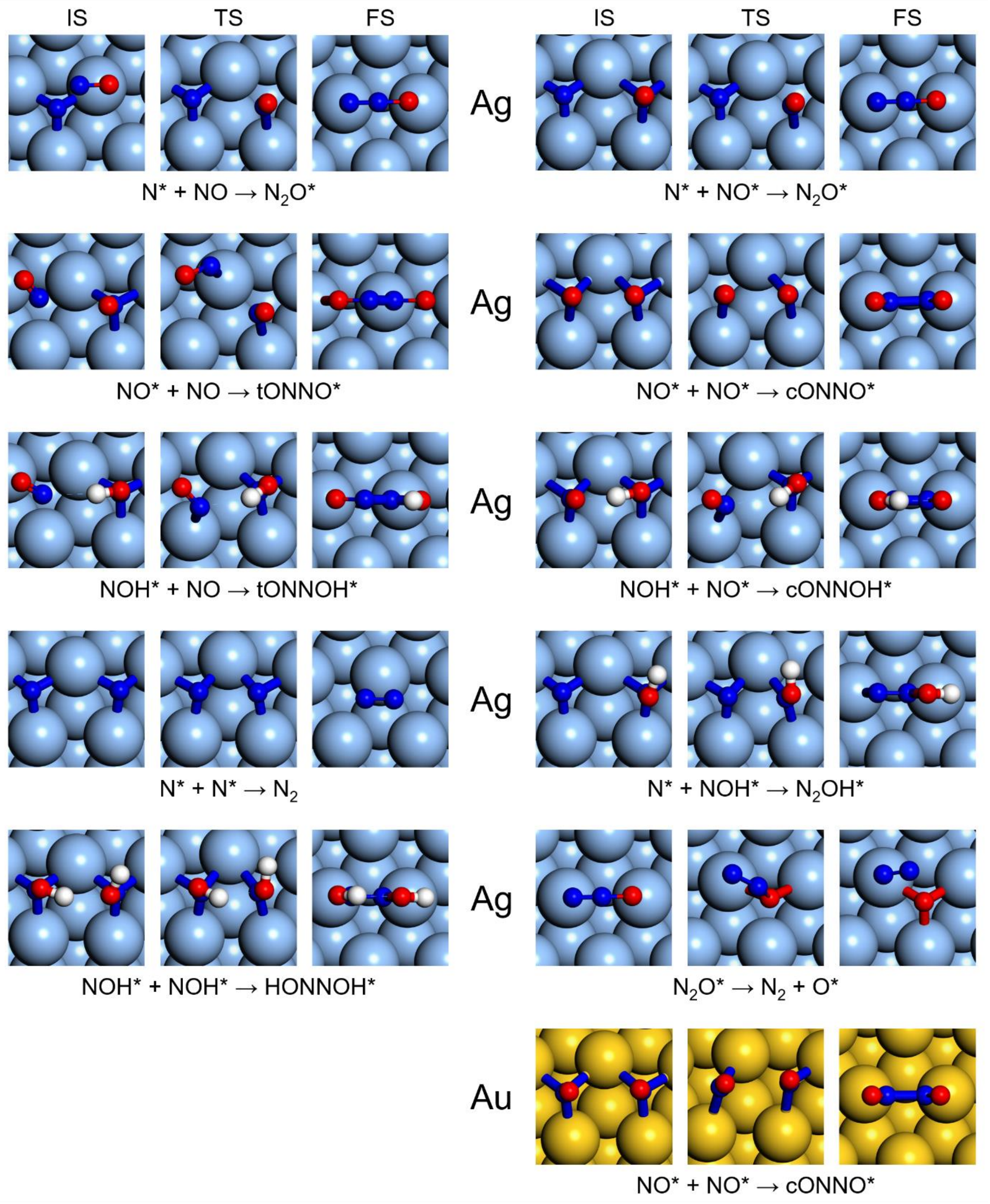

Supplementary Fig. 5 Calculated adsorption structures and the transition states (TSs) of thermochemical N-N coupling and N-O breaking steps on Ag(111) and Au(111) surfaces (c=cis, t=trans). As mentioned above, cONNO* is unstable on Fe, Ni and Pd, while it can stably adsorb on Cu, Au and Ag. Therefore, the barrier of two NO* coupling was explicitly calculated on Au(111) surface.

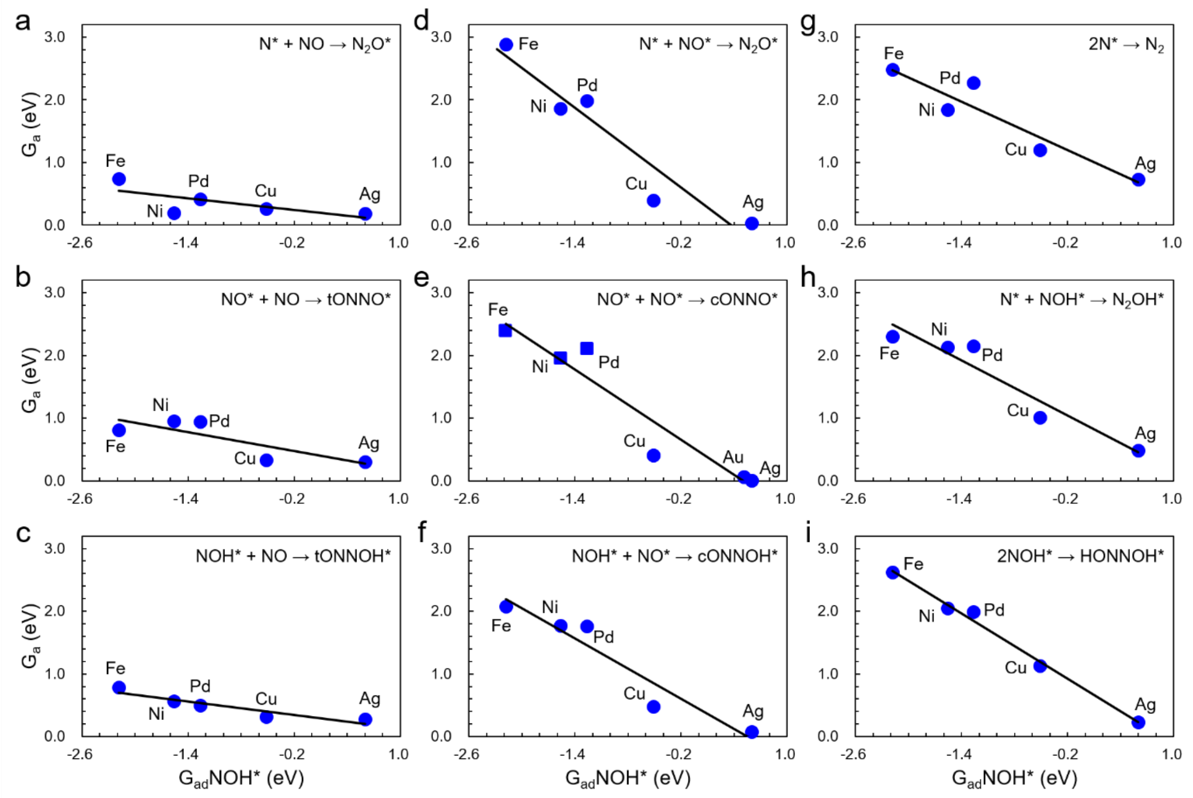

Supplementary Fig. 6 The summary and correlation between NOH* adsorption energies and kinetic barriers for various N-N coupling steps (a) N* + NO → N_2O*, (b) NO* + NO → tONNO*, (c) NOH* + NO → tONNOH*, (d) N* + NO* → N_2O* + *, (e) NO* + NO* → cONNO* + *, (f) NOH* + NO* → cONNOH* + *, (g) N* + N* → N_2 + 2*, (h) N* + NOH* → N_2OH* + *, (i) NOH* + NOH* → HONNOH*, where c=cis and t=trans. From left to right, the metals are Fe, Ni, Pd, Cu, Ag. Au is an additional case shown in Figure S6e. The data calculated by constrained optimization in Figure S6e were shown in square points.

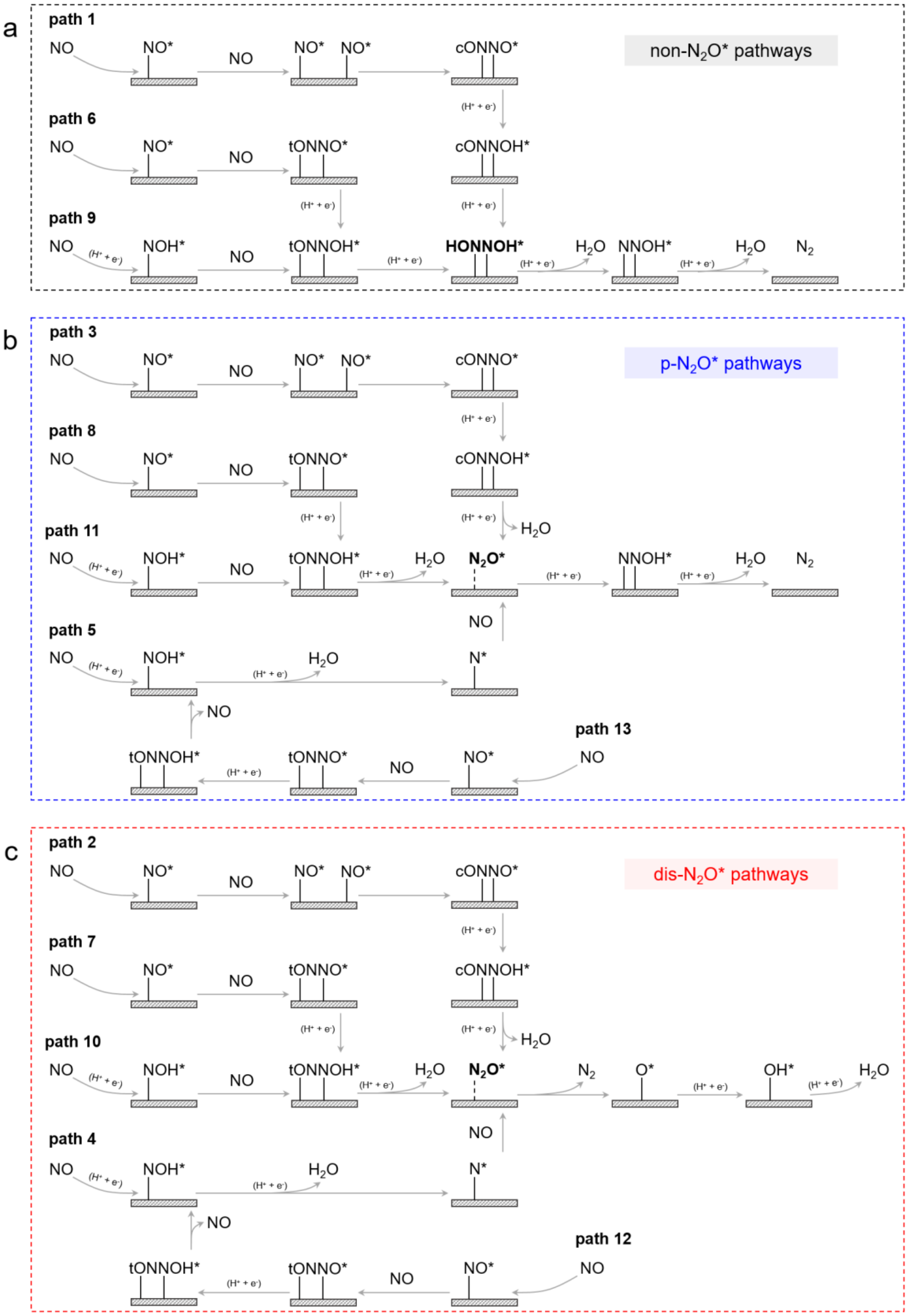

Supplementary Fig. 7 Scheme of (a) non-N_2O*, (b) p-N_2O* and (c) dis-N_2O* pathways towards N_2 production (c=cis, t=trans). The species NNOH* refers to N_2OH* and this is consistent herein, unless specified otherwise.

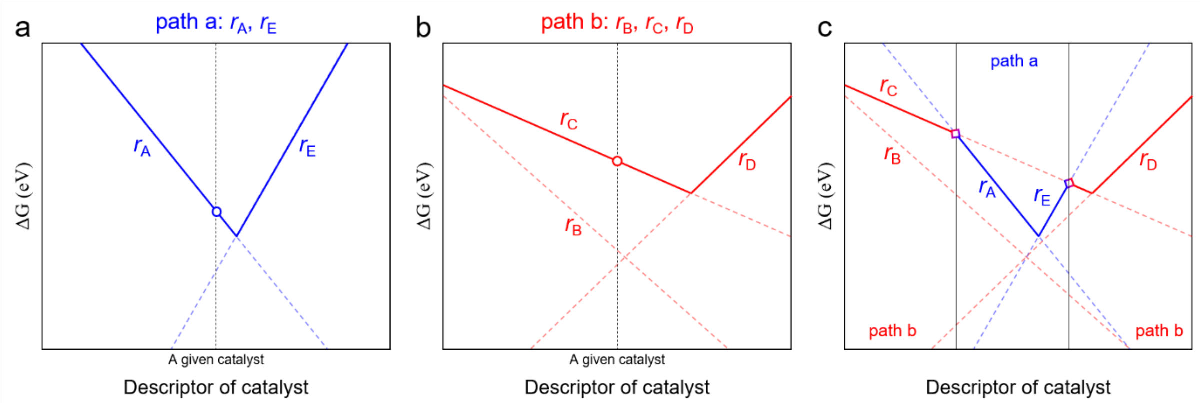

Supplementary Fig. 8 Scheme of the one-dimensional reaction phase diagram (1D-RPD) for (a) the 'path a' consisting of r_A and r_E, (b) the 'path b' consisting of r_B, r_C and r_D, and (c) the overall (optimal) activity trend with the two paths. Solid lines represent the most energetically difficult steps, namely ΔG-limiting step (r_{max}).

Note:

Take Fig. 2 in main text as an example. There are two possible paths over a given catalyst (the marked dashed vertical line in Supplementary Fig. 8) to a product. The 'path a' is consisting of r_A and r_E. The 'path b' is consisting of r_B, r_C and r_D. The ΔG-limiting step (r_{max}) for 'path a' (Supplementary Fig. 8a) is r_A, while r_C is the key step, instead of r_B, for the path b (Supplementary Fig. 8b). The preference for the two paths is justified by the comparison between r_A and r_C steps. Therefore, the 'path a', with minimum r_{max}, is the optimal path on the examplified catalyst.

As catalysts changes from one material to another, we can employ descriptors (for example, adsorption energy) to establish a reaction phase diagram (RPD) to study the evolution of reaction mechanism over a series of materials. As shown in Supplementary Fig. 8, the reaction free energies (ΔG) of elementary steps extend from points to lines. The crosspoints between the lines of r_{max} in different paths will separate RPD into several phases. For instance, as schemed in Supplementary Fig. 8c, the two crosspoints between r_A/r_E and r_C separate the RPD into three regions. On the catalysts in left and right windows, the 'path b' is the favorable path, while the optimal one in middle window is 'path a'.

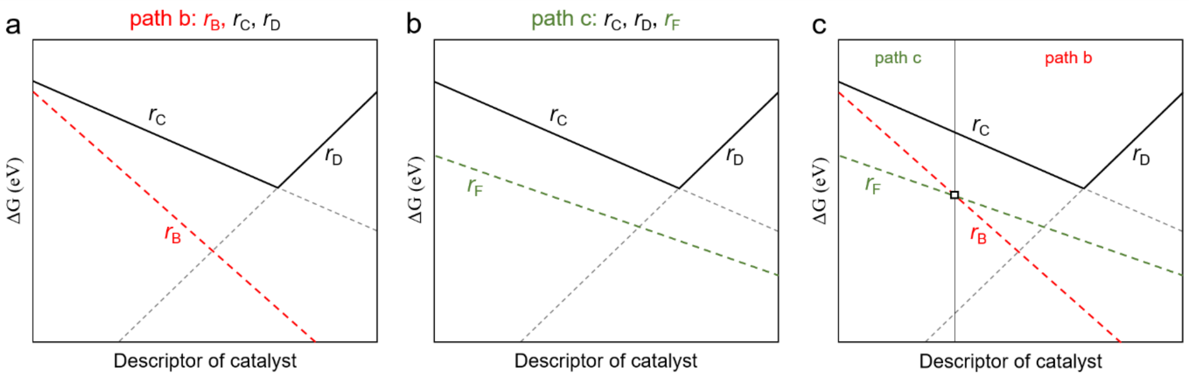

Supplementary Fig. 9 Scheme of the 1D-RPD for (a) the 'path b' consisting of r_B, r_C and r_D, (b) the 'path c' consisting of r_C, r_D and r_F, and (c) the overlapping of the two paths. Black solid lines represent the shared ΔG-limiting steps. Red and green dotted lines refer to path-selection-determining steps for 'path b' and 'path c', respectively.

Note:

Some pathways share the same ΔG-limiting steps, where the comparison upon the ΔG-limiting energy is not sufficient for selectivity analysis. The path-selection-determining steps beneath ΔG-limiting steps require to be identified and compared. As shown in Supplementary Fig. 9a and b, the r_B and r_F are identified as the selectivity-determining steps for 'path b' and 'path c', respectively. The crosspoint between r_B and r_F separates the 1D-RPD (Supplementary Fig. 9c) into two reaction phases. In the left window, the 'path c' is more preferable with the lower ΔG of r_F than that of r_B. In contrast, r_B has lower ΔG than r_F in the right phase, indicating the preference of 'path b'.

Note that the selectivity analysis among N_2 and N_2O productions was conducted through this strategy discussed in Supplementary Figs. 8 and 9, too. Taking two representative metals (left: Fe, right: Ag) as an example, the selectivity of N_2 against N_2O was analyzed by energetic comparison of key steps, as shown in Supplementary Table 5.

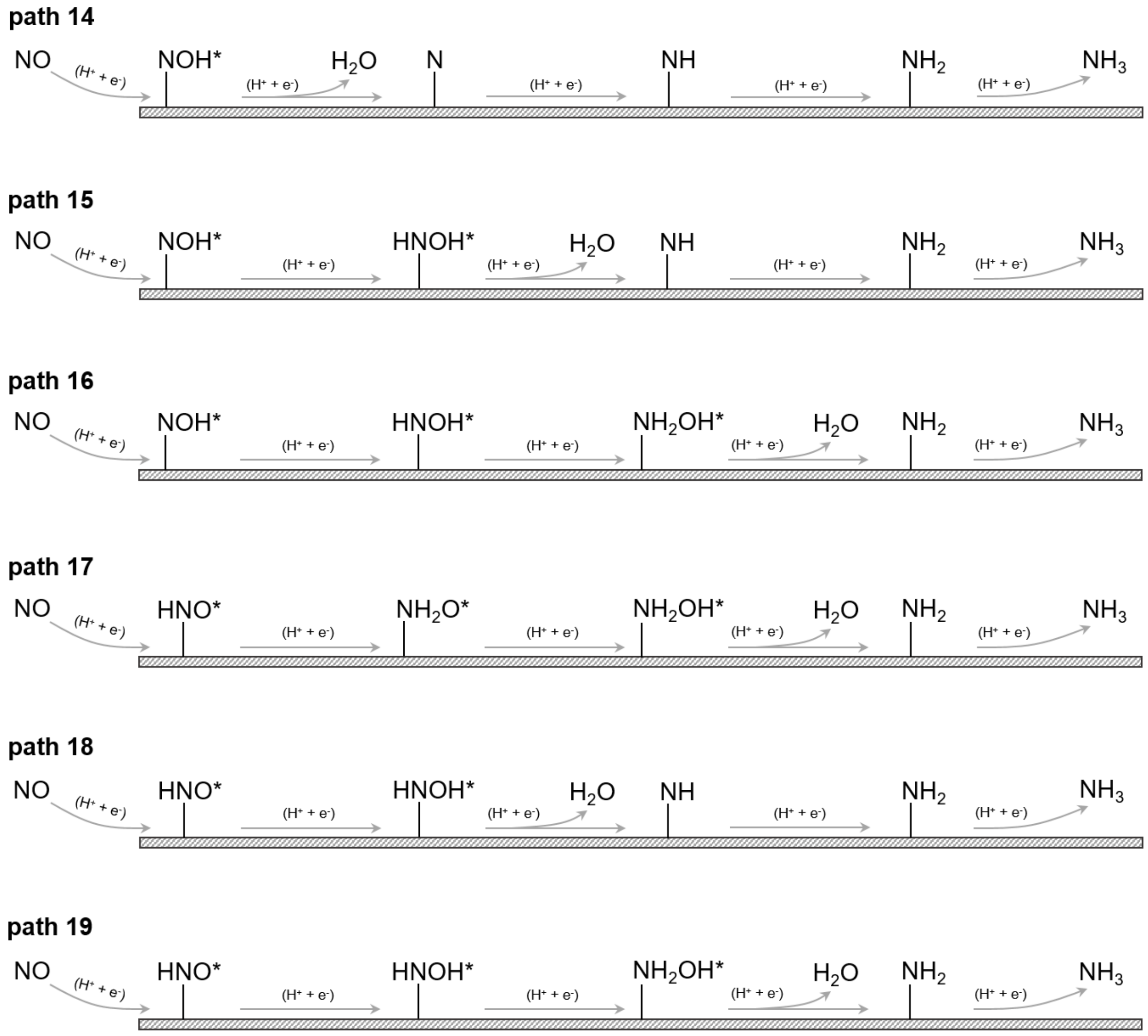

Supplementary Fig. 10 Schematic reaction mechanism for NH_3 production.

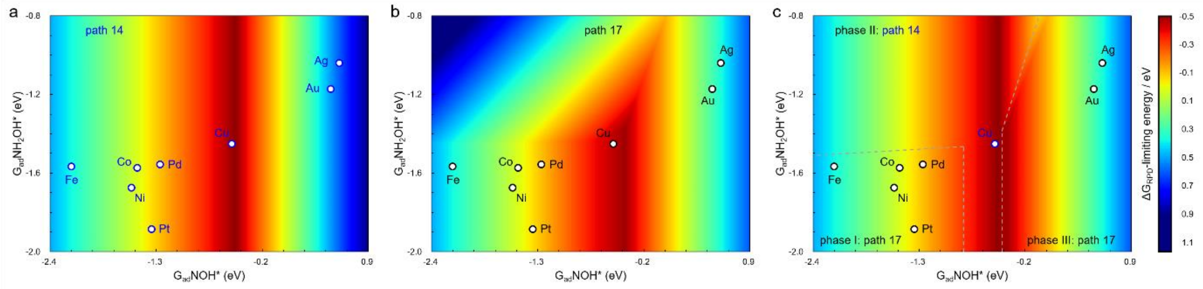

Supplementary Fig. 11 The two-dimensional reaction phase diagram (2D-RPD) for NH_3 production via (a) path 14, (b) path 17 and (c) the combination of the two paths. The grey dotted lines in (c) are the crosslines between the two paths, which separates the 2D-RPD into three phases (parts). All ΔG_{RPD}-limiting energies are referenced to the same color bar on the right.

Note:

As shown in Supplementary Fig. 8, for two individual paths with different shapes, their combination can be divided into three parts (phases). Therefore, the activity map of ammonia production was divided to three parts because the same reason. As shown in Supplementary Fig. 8c, three elementary steps can have two crosspoints (marked as square) in 1D-RPD, which can be extended as two crosslines in 2D-RPD. In Supplementary Fig. 11c, the dashed lines are from the overlapping of the two individual activity maps in Supplementary Fig. 11a and b.

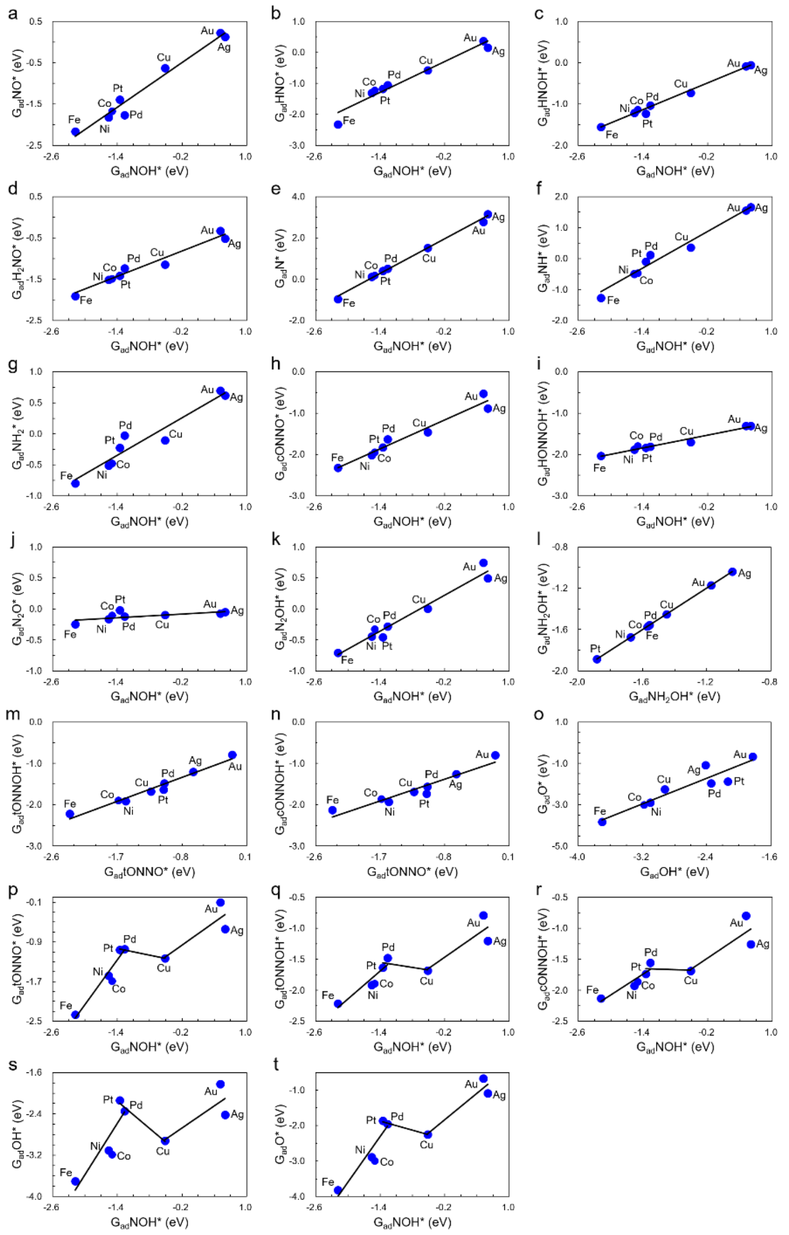

Supplementary Fig. 12 (a-k) The energetic scaling relations of most N-bonded adsorbates vs. $G_{ad}NOH^*$. (l) The second descriptor ($G_{ad}NH_2OH^*$) for ammonia and hydroxylamine production. (m-n) The energetic scaling relations of the intermediates adsorbed with N and O atoms. (o) The scaling relation between O* vs OH*. (p-t) The fitted scaling relations of O-bonded intermediates versus descriptor $G_{ad}NOH^*$, used for 1D-RPD analysis.

Note: As shown in Supplementary Fig. 28, the most of intermediates involved in eNORR are bonding with metals through N atom, where the most species were described by $G_{ad}NOH^*$ except for NH_2OH^*, which was chosen as an independent variable (Supplementary Fig. 12l) due to its importance for hydroxylamine production. The intermediates adsorbed with N and O atoms were described by $G_{ad}tONNO^*$ (Supplementary Fig. 12m and n). The $G_{ad}O^*$ was described by $G_{ad}OH^*$ (Supplementary Fig. 12o). Towards a more accurate description in one-dimensional reaction phase diagrams, their adsorption energies were fitted against $G_{ad}NOH^*$ in three stages (Supplementary Fig. 12 p-t).

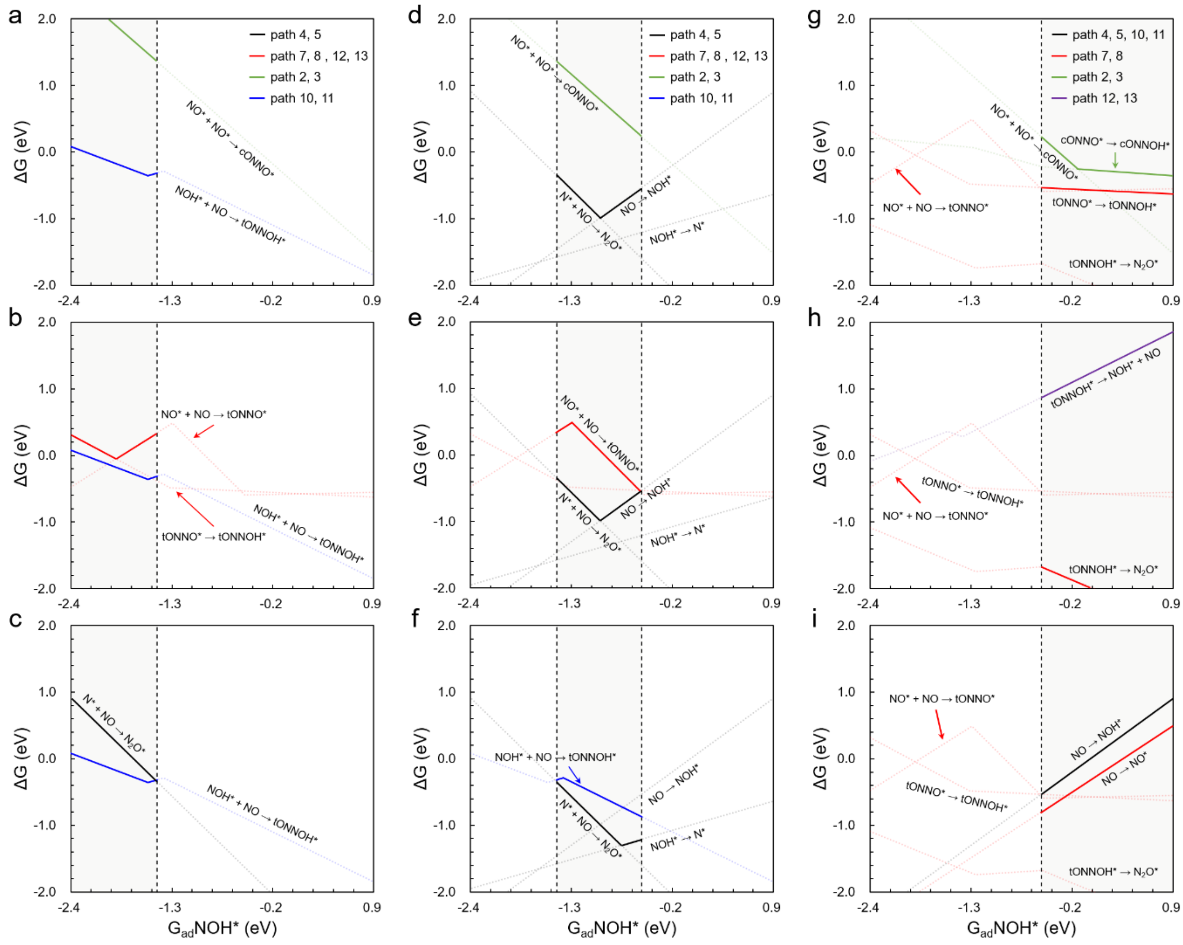

Supplementary Fig. 13 The identification of optimal paths towards N_2 in different reactivity regions, such as (a-c) $G_{ad}NOH^* < -1.46$ eV, (d-f) $G_{ad}NOH^*$ from -1.46 to -0.53 eV, and (g-i) $G_{ad}NOH^* > -0.53$ eV, by reaction phase diagram (RPD) analysis. The selective key steps for different paths were distinguished by different colors and were highlighted by solid lines in different reaction phases.

Note:

According to Fig. 4a and b in the main text, non-N_2O^* pathways (path 1, 6 and 9) were firstly excluded. In details, paths 1, 6 and 9, belonging to non-N_2O^* mechanism, yield N_2 via the key intermediate HONNOH* (Supplementary Fig. 7), indicating that the formation of HONNOH* is a necessary step for the three paths. However, as shown in Fig. 4a and b, HONNOH* formation (black dashed lines) is always more difficult than N_2O^* further conversion (blue and red dashed lines) over all studied metals. Hence, here, the further identification of optimal paths in different regions is conducted among p-N_2O^* and dis-N_2O^* paths, without discussion about paths 1, 6 and 9. From the comparison shown in Supplementary Fig. 13a, b, and c, it can be determined that the NOH*+NO coupling (blue) is the most favorable way of N-N formation for TMs with $G_{ad}NOH^* < -1.46$ eV, on which N_2 prefers to yield via N_2O^* protonation (Fig. 4a). Hence, the optimal path in left window is path11. Note that N_2 is produced by N_2O^* dissociation in reactivity region with $G_{ad}NOH^* > -1.46$ eV (Fig. 4b). Hence, from the comparison shown in Supplementary Fig. 13d, e, and f, it can be obtained that the most favorable path is path 4 with N* + NO coupling (black) in the middle window ($G_{ad}NOH^*$ from -1.46 to -0.53 eV). Finally, when $G_{ad}NOH^* > -0.53$ eV, path7 through NO* + NO coupling (red lines in Supplementary Fig. 13g, h, and i) is identified as the optimal one.

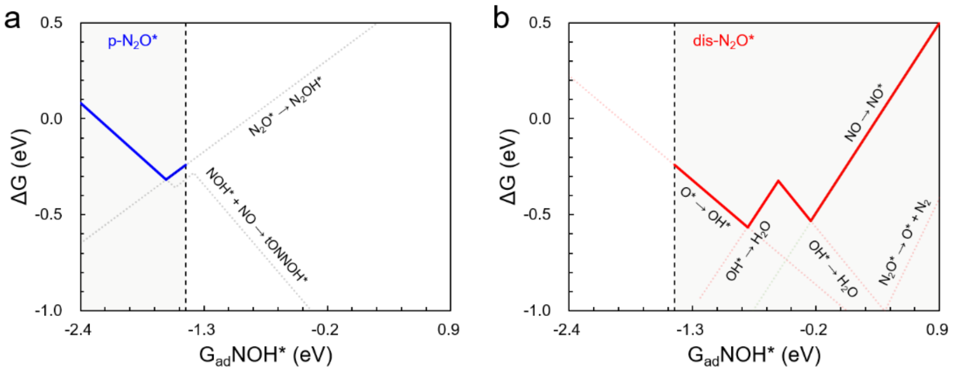

Supplementary Fig. 14 The ΔG_{RPD}-limiting steps (solid lines) of N_2 production via (a) p-N_2O* (G_{ad}NOH* < -1.46 eV) and (b) dis-N_2O* (G_{ad}NOH* > -1.46 eV) pathways.

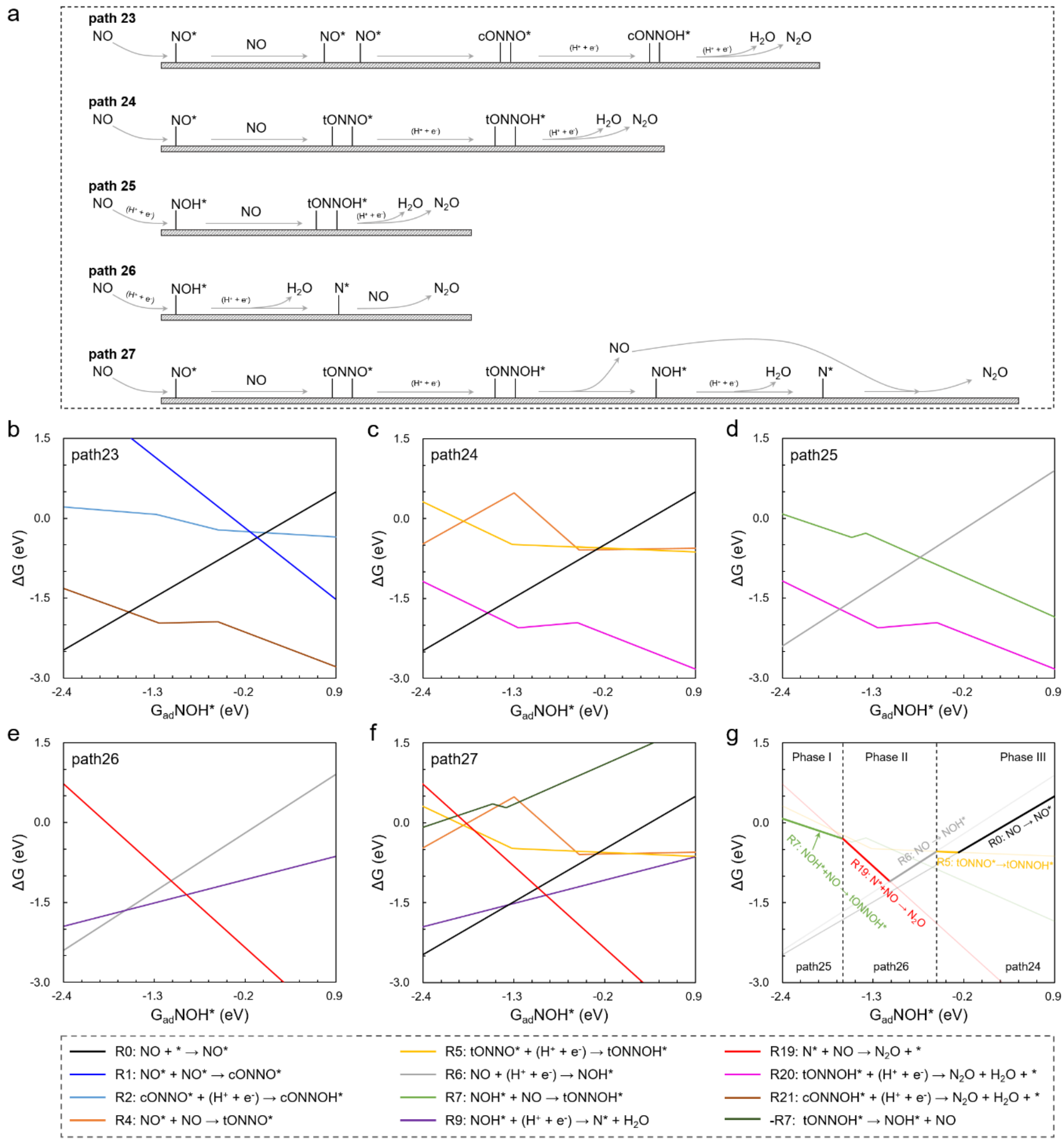

Supplementary Fig. 15 The identification of optimal paths towards N_2O in different reactivity regions, by reaction phase diagram (RPD) analysis. (a) Schematic pathways towards N_2O. (b-f) The one-dimensional (*quasi*) activity maps for path 23 to 27. (f) The three reaction phases for N_2O production, classified by N-N coupling, where the ΔG_{RPD}-limiting steps are marked in bold. All relevant steps (c=cis, t=trans) are referenced to the colored lines as marked at the bottom.

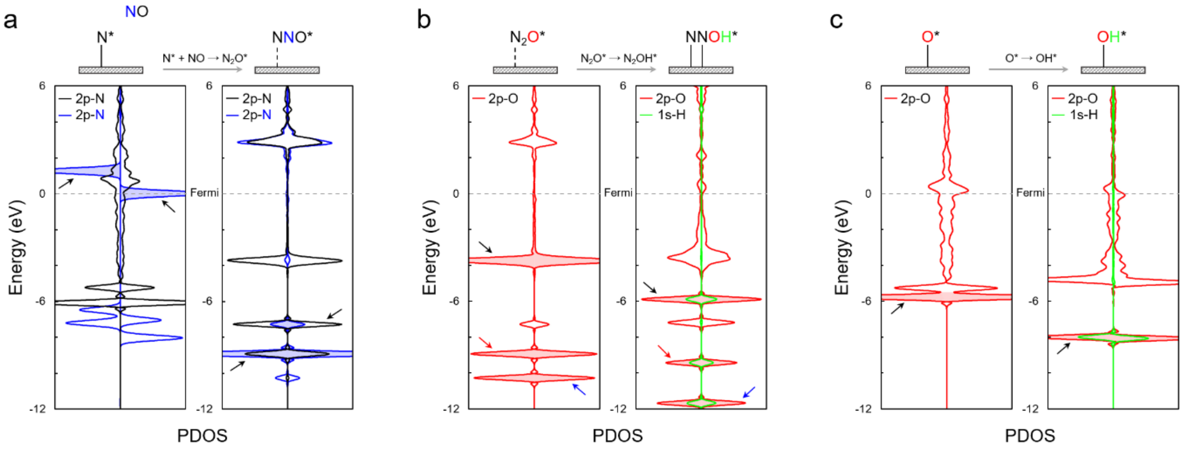

Supplementary Fig. 16 The comparison of projected density of states (PDOS) for the key three elementary steps (a) N* + NO → N_2O*, (b) N_2O* → N_2OH*, and (c) O* → OH*. The species NNO* in (a) refers to N_2O*. The 2p orbitals of adsorbed N*, N atom in NO molecule and O atoms are shown in black, blue and red lines, respectively. The 1s-H states for H atoms are shown in green.

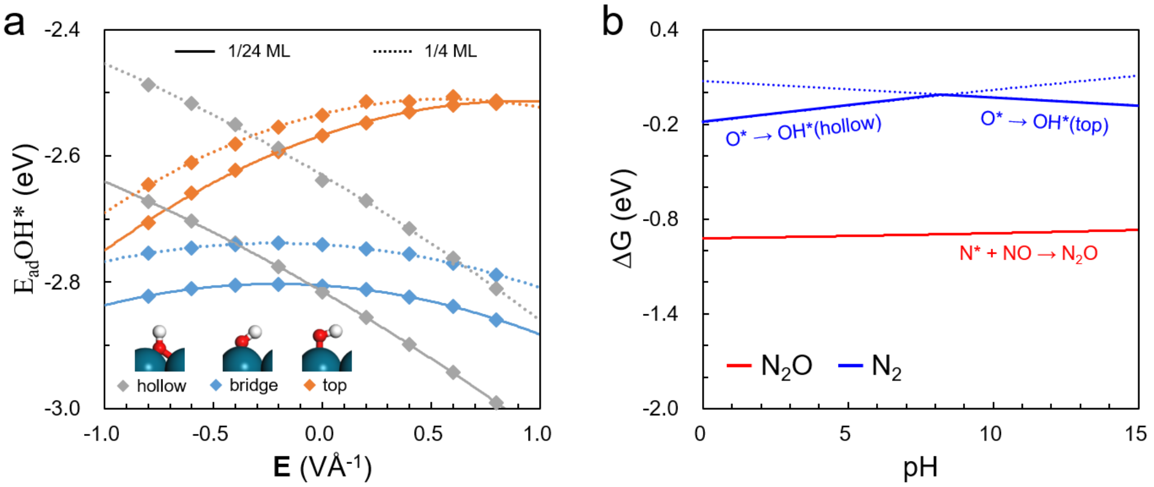

Supplementary Fig. 17 (a) The comparison of OH* adsorption at hollow, bridge, and top sites and (b) the difference of preferred reaction sites for O* → OH*. The dipole moments of OH* species are -0.20, -0.02 and 0.12 (e Å) at hollow, bridge and top sites, respectively (Supplementary Table 6).

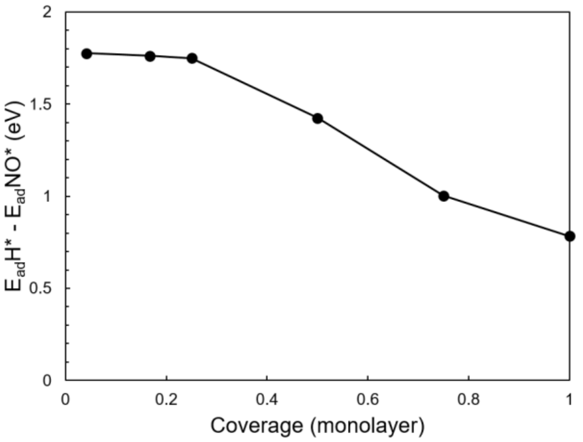

Supplementary Fig. 18 The difference in binding energies between H* and NO* on Pd.

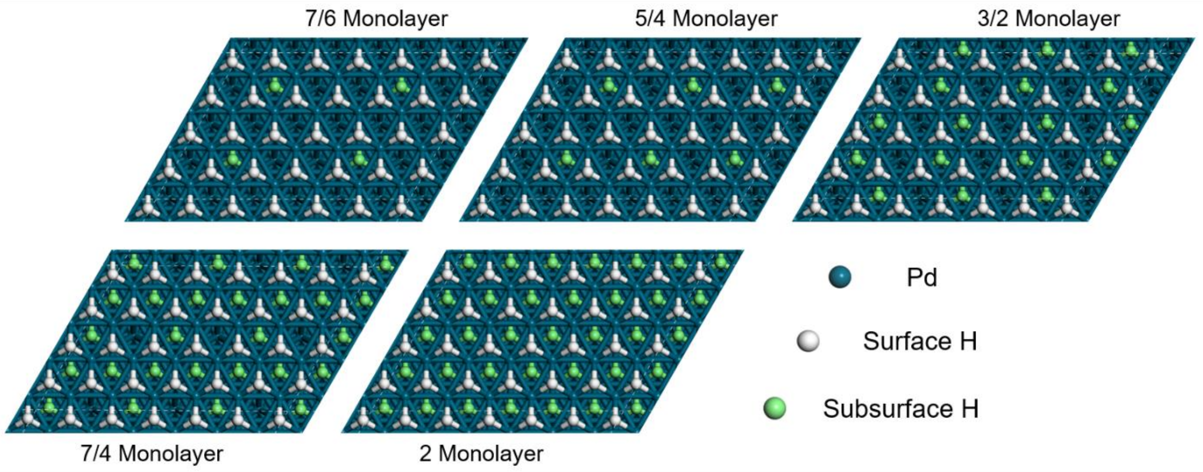

Supplementary Fig. 19 The models of H*-covered Pd, with one monolayer surface H* and varying subsurface H*.

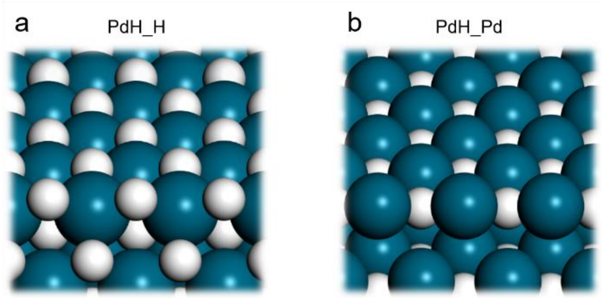

Supplementary Fig. 20 The (a) H-terminated and (b) Pd-terminated PdH(111) surfaces, which are denoted ad PdH_H and PdH_Pd, respectively.

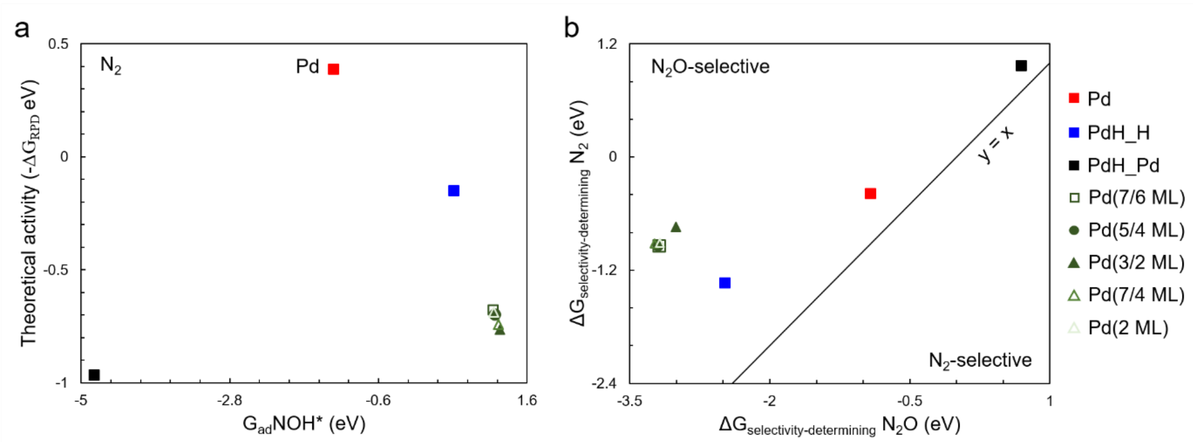

Supplementary Fig. 21 The (a) activity and (b) selectivity trends on H*-covered Pd, PdH_H and PdH_Pd surfaces.

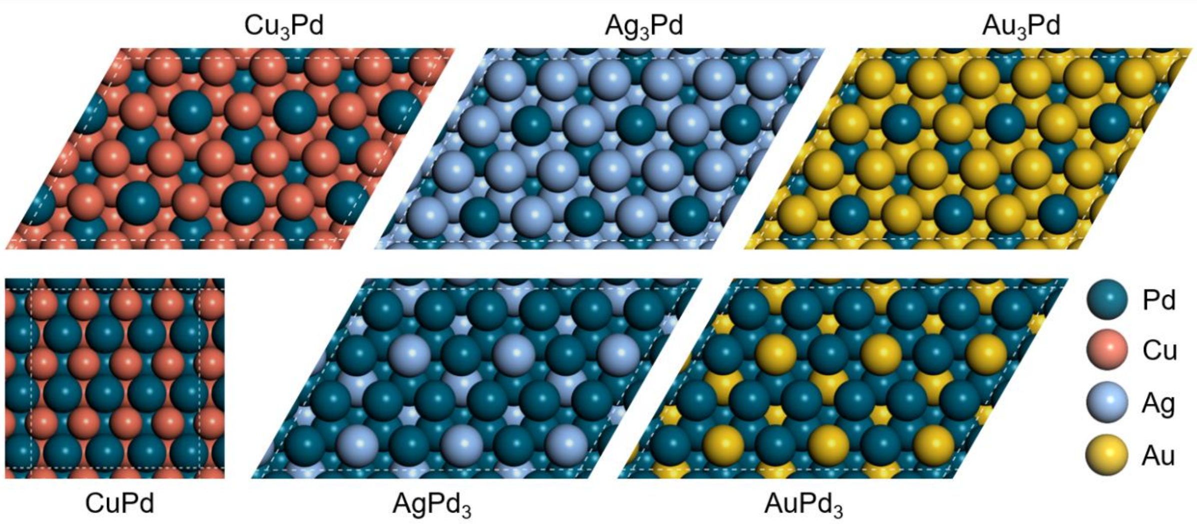

Supplementary Fig. 22 The atomic models for six Pd-based alloys, such as Cu_3Pd(111), CuPd(110), Ag_3Pd(111), $AgPd_3$(111), Au_3Pd(111) and $AuPd_3$(111) surfaces.

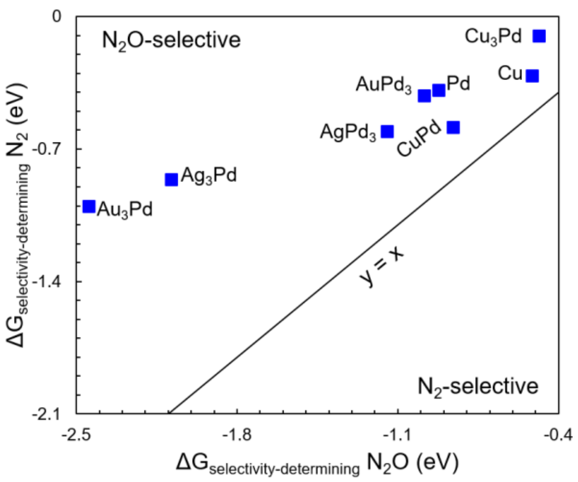

Supplementary Fig. 23 The selectivity trend for N_2 against N_2O over Cu, Pd and six Pd-based alloys.

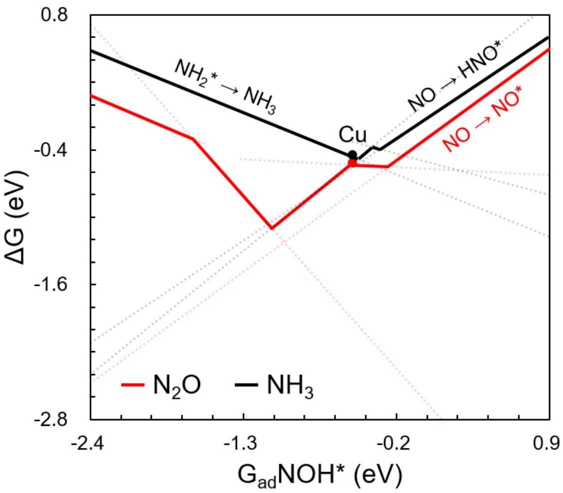

Supplementary Fig. 24 The thermodynamical analysis between N_2O and NH_3 selectivity at 0 V vs RHE. The ΔG_{RPD}-limiting steps for N_2O and NH_3 are shown in red and black solid lines, respectively.

Note:

Cu shows similar ΔG_{RPD}-limiting energies for N_2O and NH_3 production. For metals with stronger $G_{ad}NOH*$ adsorption energies than Cu (in the left of 1D-RPD), the NH_3 production has much higher ΔG-limiting energies than N_2O due to the strong adsorption of NH_2*. For Au and Ag (less reactive than Cu), the protonation of solvated NO (towards NH_3 production) is thermodynamically and kinetically difficult than its adsorption (towards dual-N products). This means the most TMs are N_2O-selective and Cu exceptionally shows comparable thermodynamic limits for N_2O and NH_3 productions.

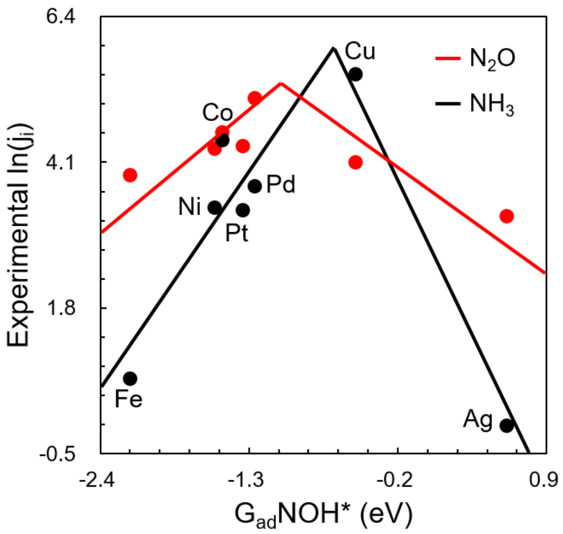

Supplementary Fig. 25 The experimental activity trends for NH_3 and N_2O productions, adopted from *J. Am. Chem. Soc.* 144, 1258–1266 (2022). From left to right, the metals are Fe, Ni, Co, Pt, Pd, Cu and Ag, respectively.

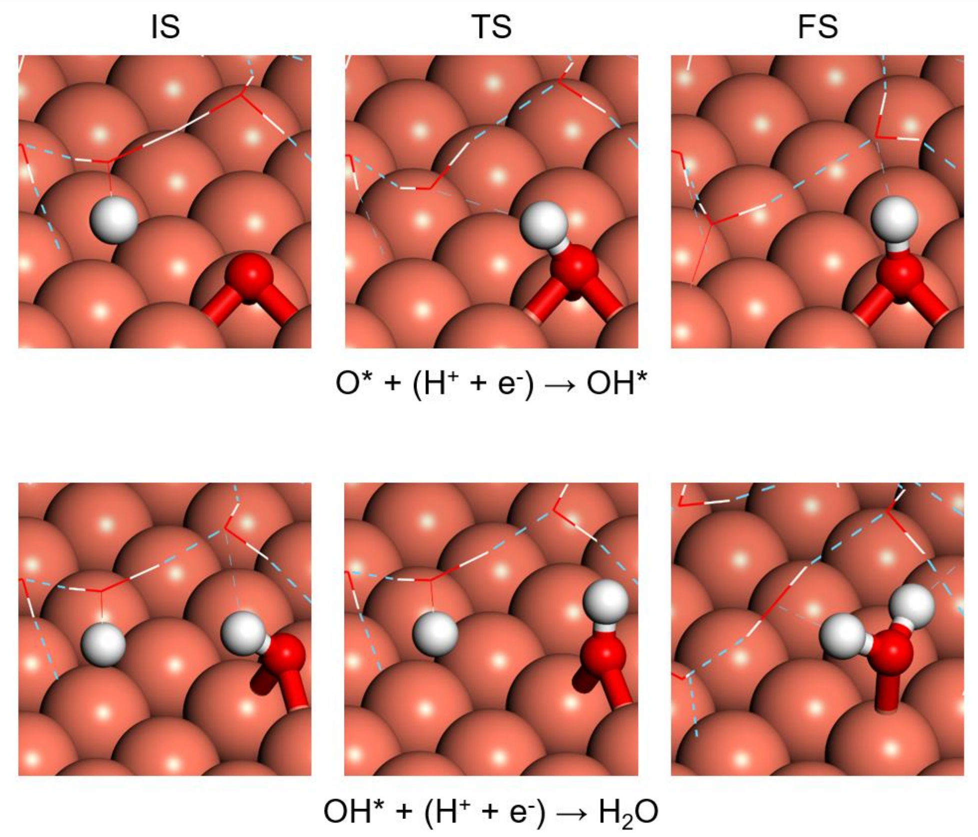

Supplementary Fig. 26 Atomic structures of initial state (IS), transition state (TS), and final state (FS) for two-step protonation of O* to H_2O on Cu.

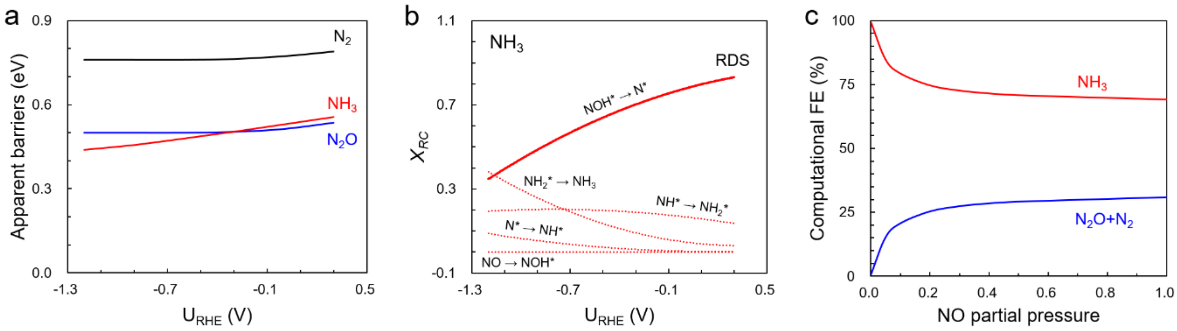

Supplementary Fig. 27 The results of microkinetic modelling on Cu. (a) The comparison of apparent activation barriers, for N_2, N_2O and NH_3 production at different potentials. (b) Degree of rate control (DRC) of different elementary steps (X_{RC}) for NH_3 formation, where rate-determining step (RDS) is shown in solid line. (c) The computational FE of NH_3 and dual-N products at different NO partial pressure, at 0.3 V vs RHE.

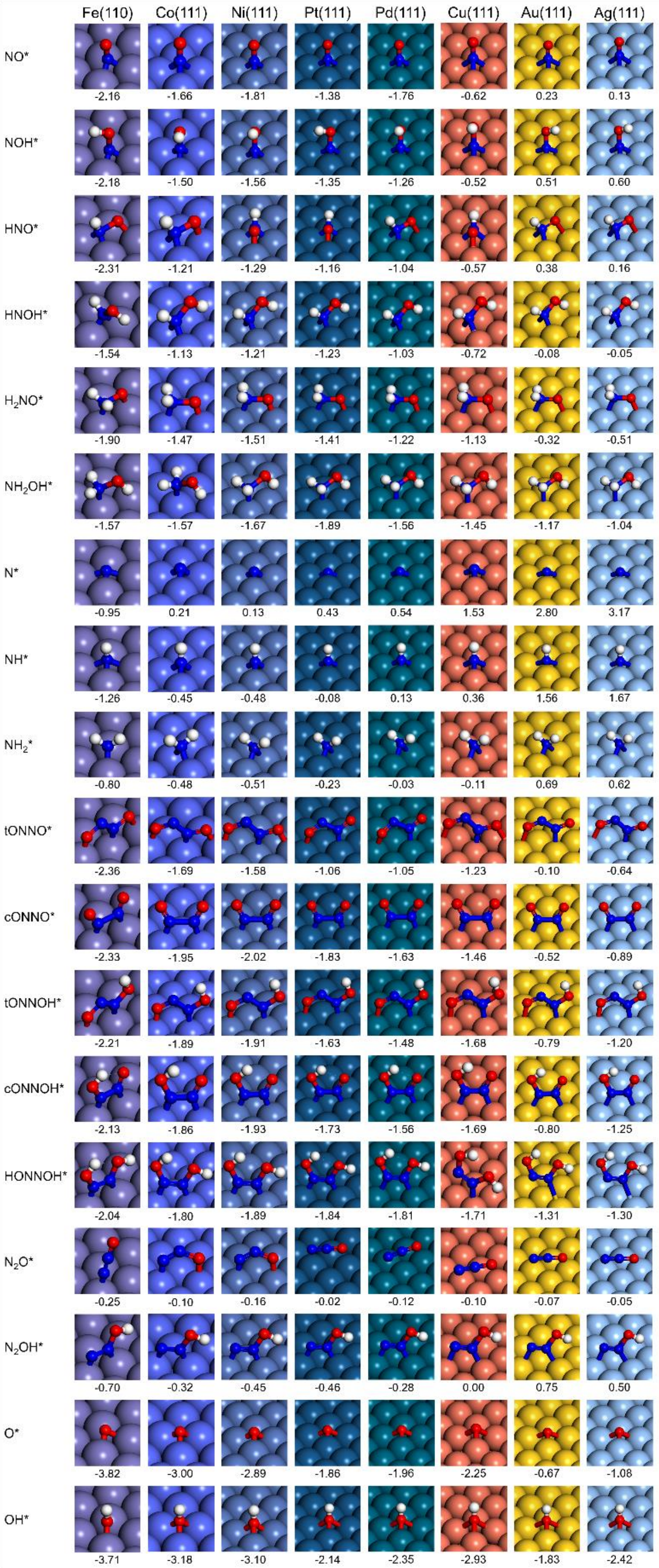

Supplementary Fig. 28 The optimized adsorption structures and corresponding adsorption free energies of all intermediates (c=cis, t=trans) in eNORR, over Fe(110), Co(111), Ni(111), Pt(111), Pd(111), Cu(111), Au(111) and Ag(111). The adsorption free energies were corrected by solvation effects (Supplementary Table 8).

Supplementary Table 1 All elementary steps considered in eNORR over different TMs. (c=cis, t=trans)

No.	Elementary reactions	No.	Elementary reactions
R0	NO + * → NO*	R17	cONNOH* + (H^+ + e^-) → HONNOH*
R1	NO* + NO* → cONNO* + *	R18	HONNOH* + (H^+ + e^-) → N_2OH* + H_2O
R2	cONNO* + (H^+ + e^-) → cONNOH*	R19	N* + NO → N_2O
R3	cONNOH* + (H^+ + e^-) → N_2O* + H_2O	R20	tONNOH* + (H^+ + e^-) → N_2O + H_2O + *
R4	NO* + NO → tONNO*	R21	cONNOH* + (H^+ + e^-) → N_2O + H_2O + *
R5	tONNO* + (H^+ + e^-) → tONNOH*	R22	NO + (H^+ + e^-) + * → HNO*
R6	NO + (H^+ + e^-) + * → NOH*	R23	NOH* + (H^+ + e^-) → HNOH*
R7	NOH* + NO → tONNOH*	R24	HNO* + (H^+ + e^-) → HNOH*
R8	tONNOH* + (H^+ + e^-) → N_2O* + H_2O	R25	HNO* + (H^+ + e^-) → NH_2O*
R9	NOH* + (H^+ + e^-) → N* + H_2O	R26	HNOH* + (H^+ + e^-) → NH* + H_2O
R10	N* + NO → N_2O*	R27	HNOH* + (H^+ + e^-) → NH_2OH*
R11	N_2O* + (H^+ + e^-) → N_2OH*	R28	NH_2O* + (H^+ + e^-) → NH_2OH*
R12	N_2OH* + (H^+ + e^-) → N_2 + H_2O + *	R29	NH_2OH* + (H^+ + e^-) → NH_2* + H_2O
R13	N_2O* → N_2 + O*	R30	N* + (H^+ + e^-) → NH*
R14	O* + (H^+ + e^-) → OH*	R31	NH* + (H^+ + e^-) → NH_2*
R15	OH* + (H^+ + e^-) → H_2O + *	R32	NH_2* + (H^+ + e^-) → NH_3 + *
R16	tONNOH* + (H^+ + e^-) → HONNOH*	R33	NH_2OH* → NH_2OH + *

Supplementary Table 2 The summary of considered pathways for N_2 production (c=cis, t=trans).

N-N coupling	non-N_2O^* pathways	dis-N_2O^* pathways	p-N_2O^* pathways
NO* + NO*	path 1:<br>NO → NO*<br>2NO* → cONNO*<br>cONNO* → cONNOH*<br>cONNOH* → HONNOH*<br>HONNOH* → N_2OH^*<br>N_2OH^* → N_2	path 2:<br>NO → NO*<br>2NO* → cONNO*<br>cONNO* → cONNOH*<br>cONNOH* → N_2O^*<br>N_2O^* → N_2+O*<br>O* → OH*<br>OH* → H_2O	path 3:<br>NO → NO*<br>2NO* → cONNO*<br>cONNO* → cONNOH*<br>cONNOH* → N_2O^*<br>N_2O^* → N_2OH^*<br>N_2OH^* → N_2
N* + NO	————————	path 4:<br>NO → NOH*<br>NOH* → N*<br>N*+NO → N_2O^*<br>N_2O^* → N_2+O*<br>O* → OH*<br>OH* → H_2O	path 5:<br>NO → NOH*<br>NOH* → N*<br>N*+NO → N_2O^*<br>N_2O^* → N_2OH^*<br>N_2OH^* → N_2
NO* + NO	path 6:<br>NO → NO*<br>NO*+NO → tONNO*<br>tONNO* → tONNOH*<br>tONNOH* → HONNOH*<br>HONNOH* → N_2OH^*<br>N_2OH^* → N_2	path 7:<br>NO → NO*<br>NO*+NO → tONNO*<br>tONNO* → tONNOH*<br>tONNOH* → N_2O^*<br>N_2O^* → N_2+O*<br>O* → OH*<br>OH* → H_2O	path 8:<br>NO → NO*<br>NO*+NO → tONNO*<br>tONNO* → tONNOH*<br>tONNOH* → N_2O^*<br>N_2O^* → N_2OH^*<br>N_2OH^* → N_2
NOH* + NO	path 9:<br>NO → NOH*<br>NOH*+NO → tONNOH*<br>tONNOH* → HONNOH*<br>HONNOH* → N_2OH^*<br>N_2OH^* → N_2	path 10:<br>NO → NOH*<br>NOH*+NO → tONNOH*<br>tONNOH* → N_2O^*<br>N_2O^* → N_2+O*<br>O* → OH*<br>OH* → H_2O	path 11:<br>NO → NOH*<br>NOH*+NO → tONNOH*<br>tONNOH* → N_2O^*<br>N_2O^* → N_2OH^*<br>N_2OH^* → N_2
Cyclic coupling	——————————	path 12:<br>NO → NO*<br>NO*+NO → tONNO*<br>tONNO* → tONNOH*<br>tONNOH* → NOH*+NO<br>NOH* → N*<br>N*+NO → N_2O^*<br>N_2O^* → N_2+O*<br>O* → OH*<br>OH* → H_2O	path 13:<br>NO → NO*<br>NO*+NO → tONNO*<br>tONNO* → tONNOH*<br>tONNOH* → NOH*+NO<br>NOH* → N*<br>N*+NO → N_2O^*<br>N_2O^* → N_2OH^*<br>N_2OH^* → N_2

Note that the tag of "Cyclic coupling" means that the formation of N_2O^* proceeds the following route: NO* + NO → tONNO* → tONNOH* → NOH* + NO → N* + NO → N_2O^*.

Supplementary Table 3 The summary of considered pathways for NH_3 and NH_2OH production.

NH_3 production		
path 14:	path 15:	path 16:
NO → NOH*	NO → NOH*	NO → NOH*
NOH* → N*	NOH* → HNOH*	NOH* → HNOH*
N* → NH*	HNOH* → NH*	HNOH* → NH_2OH*
NH* → NH_2*	NH* → NH_2*	NH_2OH* → NH_2*
NH_2* → NH_3	NH_2* → NH_3	NH_2* → NH_3
path 17:	path 18:	path 19:
NO → HNO*	NO → HNO*	NO → HNO*
HNO* → NH_2O*	HNO* → HNOH*	HNO* → HNOH*
NH_2O* → NH_2OH*	HNOH* → NH*	HNOH* → NH_2OH*
NH_2OH* → NH_2*	NH* → NH_2*	NH_2OH* → NH_2*
NH_2* → NH_3	NH_2* → NH_3	NH_2* → NH_3

NH_2OH production		
path 20:	path 21:	path 22:
NO → HNO*	NO → HNO*	NO → NOH*
HNO* → NH_2O*	HNO* → HNOH*	NOH* → HNOH*
NH_2O* → NH_2OH*	HNOH* → NH_2OH*	HNOH* → NH_2OH*
NH_2OH* → NH_2OH + *	NH_2OH* → NH_2OH + *	NH_2OH* → NH_2OH + *

Supplementary Table 4 The optimal paths towards different products on all studied TMs.

	N_2	N_2O	NH_3	NH_2OH
Fe	path 11	path 25	path 17	path 20
Co	path 11	path 26	path 17	path 20
Ni	path 11	path 26	path 17	path 20
Pt	path 4	path 26	path 17	path 20
Pd	path 4	path 26	path 17	path 20
Cu	path 4	path 26	path 14	path 20
Au	path 7	path 24	path 17	path 20
Ag	path 7	path 24	path 17	path 20

Note:

Towards N_2O, the pathways (path 23 to 27) were schematically shown in Supplementary Fig. 15a.

Supplementary Table 5 The selectivity analysis between N_2O and N_2 production by selectivity-determining energy (eV).

		NO → NOH^*	NO + NOH^* → $tONNOH^*$	$tONNOH^*$ → $N_2O + H_2O$	$tONNOH^*$ → $N_2O^* + H_2O$	N_2O^* → N_2OH^*	N_2OH^* → $N_2 + H_2O$
Fe	N_2O	-2.18	-0.03	-1.34			
	Note		ΔG-limiting	selectivity determining			
	N_2	-2.18	-0.03		-1.21	-0.55	-2.52
	Note		ΔG-limiting			selectivity determining	

		NO → NO^*	NO + NO^* → $tONNO^*$	$tONNO^*$ → $tONNOH^*$	$tONNOH^*$ → $N_2O + H_2O$	$tONNOH^*$ → $N_2O^* + H_2O$	N_2O^* → $N_2 + O^*$	O^* → OH^*	OH^* → H_2O
Ag	N_2O	0.23	-0.56	-0.63	-2.64				
	Note	ΔG-limiting			selectivity determining				
	N_2	0.23	-0.56	-0.63		-2.32	-0.78	-1.26	-1.14
	Note	ΔG-limiting					selectivity determining		

Note:

Over Fe, N_2 and N_2O production share the same process: 2NO → NO + NOH^* → $tONNOH^*$. The follow-up dehydration of $tONNOH^*$ directly releases N_2O (-1.34 eV) is the selective-determining step for N_2O production. Towards N_2, the further protonation of (in-situ) generated N_2O^* is thermodynamically difficult (-0.55 eV) due to the relatively weak adsorption of N_2OH^*, which is rationally identified as the selective-determining step. Compared to N_2O release, the higher ΔG of N_2O^* protonation reveals the lower selectivity of N_2 than N_2O.

Analogously, for Ag, N_2O release (-2.64 eV) and N_2O^* dissociation (-0.78 eV) are the selective-determining steps for N_2O and N_2 production, respectively. The more difficult N_2O^* dissociation indicates the lower selectivity of N_2 against N_2O.

Supplementary Table 6 The fitted values of μ (e Å) and α (e $Å^2$ V^{-1}).

Species	Pd		Cu	
	μ	α	μ	α
NOH*	-0.17	0.22	-0.05	0.23
N*	0.03	0.02	0.12	0.003
N_2O*	-0.08	0.35	-0.05	0.19
O*	0.07	0.002	0.13	0.004
OH*-hollow	-0.20	0.05	-0.19	0.05
OH*-bridge	-0.02	0.11	—	—
OH*-top	0.12	0.13	—	—
NH*	—	—	-0.08	0.02
NH_2*	—	—	-0.17	0.08
$TS_{N*+NO→N2O*}$	—	—	0.07	0.26

Supplementary Table 7 The potential dependent energetics (eV) of optimal paths to N_2, N_2O, NH_3 on Cu.

Barriers G_a						
Elementary reactions	RHE potentials (V)					
	0.3	0	-0.3	-0.6	-0.9	-1.2
$NO + (H^+ + e^-) + * \rightarrow NOH*$	0.03	0.02	0.01	0	0	0
$NOH* + (H^+ + e^-) \rightarrow N* + H_2O$	0.56	0.54	0.52	0.49	0.47	0.44
$N* + (H^+ + e^-) \rightarrow NH*$	0.42	0.42	0.42	0.41	0.41	0.41
$NH* + (H^+ + e^-) \rightarrow NH_2*$	0.52	0.50	0.48	0.46	0.45	0.43
$NH_2* + (H^+ + e^-) \rightarrow NH_3 + *$	0.48	0.47	0.46	0.46	0.45	0.45
$N* + NO \rightarrow N_2O*$	0.50	0.50	0.50	0.50	0.50	0.50
$N_2O* \rightarrow N_2 + O*$	0.26	0.26	0.26	0.26	0.26	0.26
$O* + (H^+ + e^-) \rightarrow OH*$	0.38	0.38	0.38	0.37	0.37	0.37
$OH* + (H^+ + e^-) \rightarrow H_2O$	0.44	0.44	0.44	0.43	0.43	0.43

Reaction free energies ΔG						
Elementary reactions	RHE potentials (V)					
	0.3	0	-0.3	-0.6	-0.9	-1.2
$NO + (H^+ + e^-) + * \rightarrow NOH*$	-0.23	-0.53	-0.83	-1.13	-1.43	-1.73
$NOH* + (H^+ + e^-) \rightarrow N* + H_2O$	-0.96	-1.26	-1.56	-1.86	-2.16	-2.46
$N* + (H^+ + e^-) \rightarrow NH*$	-0.73	-1.03	-1.33	-1.63	-1.93	-2.23
$NH* + (H^+ + e^-) \rightarrow NH_2*$	-0.12	-0.42	-0.72	-1.02	-1.32	-1.62
$NH_2* + (H^+ + e^-) \rightarrow NH_3 + *$	-0.06	-0.36	-0.66	-0.96	-1.26	-1.56
$N* + NO \rightarrow N_2O*$	-1.56	-1.56	-1.56	-1.56	-1.56	-1.56
$N_2O* \rightarrow N_2 + O*$	-2.23	-2.23	-2.23	-2.23	-2.23	-2.23
$O* + (H^+ + e^-) \rightarrow OH*$	-0.16	-0.46	-0.76	-1.06	-1.36	-1.66
$OH* + (H^+ + e^-) \rightarrow H_2O$	-0.14	-0.44	-0.77	-1.04	-1.34	-1.64

Supplementary Table 8 Solvation energies for different intermediates, via implicit solvent model (t: trans; c: cis).

Species	Solvation energies (eV)
NO*	-0.04
NOH*	-0.25
HNO*	-0.20
HNOH*	-0.20
H_2NO*	-0.36
NH_2OH*	-0.35
N*	-0.02
NH*	-0.11
NH_2*	-0.18
tONNO*	-0.18
cONNO*	-0.39
tONNOH*	-0.24
cONNOH*	-0.20
HONNOH*	-0.52
N_2O*	-0.08
N_2OH*	-0.21
O*	-0.04
OH*	-0.17

Supplementary Table 9 The comparison between implicit solvent and explicit solvent models (eV).

	Implicit solvation	Explicit solvation			
		Case 1	Case 2	Case 3	average
tONNO*	-0.18	-0.22	-0.27	-0.22	-0.24
cONNO*	-0.39	-0.48	-0.40	-0.31	-0.40
