# Supplementary material for: Steering from electrochemical denitrification to ammonia synthesis
Source: Nat Commun. 2023 Jan 7;14:112. doi: 10.1038/s41467-023-35785-w (PMC9825404; doi:10.1038/s41467-023-35785-w)
Supplement: Supplementary file 2 — Description of Additional Supplementary Files [file 41467_2023_35785_MOESM2_ESM.pdf]

Supplementary Dataset 1 The optimized structures of six Pd-based alloys and two PdH surfaces, which are Cu_3Pd, CuPd, Ag_3Pd, $AgPd_3$, Au_3Pd, $AuPd_3$, PdH_H (H terminal of PdH(111) surface) and PdH_Pd (Pd terminal of PdH(111) surface), respectively.

Supplementary Dataset 2 The source data of Fig. 1, 3, 4, 6 and 7.
