# Supplementary material for: Steering from electrochemical denitrification to ammonia synthesis
Source: Nat Commun. 2023 Jan 7;14:112. doi: 10.1038/s41467-023-35785-w (PMC9825404; doi:10.1038/s41467-023-35785-w)
Supplement: Supplementary file 3 — Supplementary Dataset 1 [file 41467_2023_35785_MOESM3_ESM.pdf]

```
Cu3Pd111opt
   1.00000000000000
     7.5334339999999997    -7.5334339999999997     0.0000000000000000
     0.0000000000000000    11.3001509999999996   -11.3001509999999996
    12.4269710000000000    12.4269710000000000    12.4269710000000000
   Cu   Pd
    72    24
Selective dynamics
Direct
  0.2988130000000027  0.1404169999999993  0.0929190000000020   F   F   F
  0.1321459999999988  0.2515289999999979  0.1939550000000025   F   F   F
  0.4628096394875612  0.0283494180588111  0.2916991319150147   T   T   T
  0.2929777913535174  0.1384344188896412  0.3894964997597515   T   T   T
  0.0488130000000027  0.3070840000000032  0.0929190000000020   F   F   F
  0.3821459999999988  0.0848620000000011  0.1939550000000025   F   F   F
  0.2163613684613023  0.1973880700599304  0.2917435789351590   T   T   T
  0.0511978333626411  0.3105458628277723  0.3893254205547870   T   T   T
  0.0488130000000027  0.1404169999999993  0.0929190000000020   F   F   F
  0.3821459999999988  0.2515289999999979  0.1939550000000025   F   F   F
  0.2164559237805968  0.0282729599298956  0.2916938304698645   T   T   T
  0.0514101344531286  0.1384856926756347  0.3895545875377118   T   T   T
  0.7988130000000027  0.1404169999999993  0.0929190000000020   F   F   F
  0.6321459999999988  0.2515289999999979  0.1939550000000025   F   F   F
  0.9628096770827517  0.0283494252348801  0.2916991284309427   T   T   T
  0.7929777829499163  0.1384343914079552  0.3894964916644628   T   T   T
  0.5488130000000027  0.3070840000000032  0.0929190000000020   F   F   F
  0.8821459999999988  0.0848620000000011  0.1939550000000025   F   F   F
  0.7163613553857255  0.1973880460876743  0.2917435687526779   T   T   T
  0.5511978392074433  0.3105458651864921  0.3893253994938701   T   T   T
  0.5488130000000027  0.1404169999999993  0.0929190000000020   F   F   F
  0.8821459999999988  0.2515289999999979  0.1939550000000025   F   F   F
  0.7164559265001813  0.0282729578842559  0.2916938148477274   T   T   T
  0.5514101373386269  0.1384857056926833  0.3895545809975798   T   T   T
  0.2988130000000027  0.4737510000000000  0.0929190000000020   F   F   F
  0.1321459999999988  0.5848620000000011  0.1939550000000025   F   F   F
  0.4629045530064088  0.3616802957932577  0.2917055381311494   T   T   T
  0.2930679182545864  0.4717745283036957  0.3895134577907750   T   T   T
  0.0488130000000027  0.6404169999999993  0.0929190000000020   F   F   F
  0.3821459999999988  0.4181949999999972  0.1939550000000025   F   F   F
  0.2163944930188481  0.5307071595689973  0.2917442398516604   T   T   T
  0.0512703260313956  0.6440246831575477  0.3893501308206271   T   T   T
  0.0488130000000027  0.4737510000000000  0.0929190000000020   F   F   F
  0.3821459999999988  0.5848620000000011  0.1939550000000025   F   F   F
  0.2165203643486273  0.3615496474496931  0.2916983761725748   T   T   T
```

0.0513887960384290	0.4718102488672419	0.3895620748356091	T	T	T
0.7988130000000027	0.4737510000000000	0.0929190000000020	F	F	F
0.6321459999999988	0.5848620000000011	0.1939550000000025	F	F	F
0.9629045940866220	0.3616803006855833	0.2917055297488086	T	T	T
0.7930679323851932	0.4717745196066413	0.3895134150328506	T	T	T
0.5488130000000027	0.6404169999999993	0.0929190000000020	F	F	F
0.8821459999999988	0.4181949999999972	0.1939550000000025	F	F	F
0.7163944649714826	0.5307071549587825	0.2917442285991395	T	T	T
0.5512703375124366	0.6440247014595821	0.3893501443967136	T	T	T
0.5488130000000027	0.4737510000000000	0.0929190000000020	F	F	F
0.8821459999999988	0.5848620000000011	0.1939550000000025	F	F	F
0.7165203523919510	0.3615496339684974	0.2916983567327627	T	T	T
0.5513887968043807	0.4718102542902613	0.3895620887476205	T	T	T
0.2988130000000027	0.8070840000000032	0.0929190000000020	F	F	F
0.1321459999999988	0.9181949999999972	0.1939550000000025	F	F	F
0.4628394716930576	0.6951056401325567	0.2916922042039810	T	T	T
0.2929910832689177	0.8050928475156790	0.3895152278824590	T	T	T
0.0488130000000027	0.9737510000000000	0.0929190000000020	F	F	F
0.3821459999999988	0.7515289999999979	0.1939550000000025	F	F	F
0.2164321937096439	0.8640901038961706	0.2917564448628071	T	T	T
0.0513368412193392	0.9772504495875314	0.3893507447449544	T	T	T
0.0488130000000027	0.8070840000000032	0.0929190000000020	F	F	F
0.3821459999999988	0.9181949999999972	0.1939550000000025	F	F	F
0.2165532220305914	0.6950010340450268	0.2916904776633468	T	T	T
0.0512787400670606	0.8051511967435716	0.3895565778048691	T	T	T
0.7988130000000027	0.8070840000000032	0.0929190000000020	F	F	F
0.6321459999999988	0.9181949999999972	0.1939550000000025	F	F	F
0.9628394926211685	0.6951056432178995	0.2916921963329279	T	T	T
0.7929910954445362	0.8050928503705482	0.3895152231685473	T	T	T
0.5488130000000027	0.9737510000000000	0.0929190000000020	F	F	F
0.8821459999999988	0.7515289999999979	0.1939550000000025	F	F	F
0.7164321913287561	0.8640900925107061	0.2917564376311854	T	T	T
0.5513368396806787	0.9772504427731513	0.3893507328986814	T	T	T
0.5488130000000027	0.8070840000000032	0.0929190000000020	F	F	F
0.8821459999999988	0.9181949999999972	0.1939550000000025	F	F	F
0.7165532143824043	0.6950010474104890	0.2916904753251287	T	T	T
0.5512787189028669	0.8051512142162953	0.3895565770528088	T	T	T
0.2988130000000027	0.3070840000000032	0.0929190000000020	F	F	F
0.1321459999999988	0.0848620000000011	0.1939550000000025	F	F	F
0.4652498389903136	0.1957759024555949	0.2920566533611314	T	T	T
0.2985904084630502	0.3070181803088288	0.3940761518445665	T	T	T
0.7988130000000027	0.3070840000000032	0.0929190000000020	F	F	F
0.6321459999999988	0.0848620000000011	0.1939550000000025	F	F	F
0.9652498829122237	0.1957759155753557	0.2920566619954024	T	T	T

0.7985904067376254 0.3070181591093506 0.3940760795175043 T T T
0.2988130000000027 0.6404169999999993 0.0929190000000020 F F F
0.1321459999999988 0.4181949999999972 0.1939550000000025 F F F
0.4652619489601724 0.5292586846412934 0.2920705546728229 T T T
0.2984967894162224 0.6402908339555157 0.3940266332517731 T T T
0.7988130000000027 0.6404169999999993 0.0929190000000020 F F F
0.6321459999999988 0.4181949999999972 0.1939550000000025 F F F
0.9652619709224078 0.5292586841997602 0.2920705355198741 T T T
0.7984967844148152 0.6402908246103239 0.3940265795299702 T T T
0.2988130000000027 0.9737510000000000 0.0929190000000020 F F F
0.1321459999999988 0.7515289999999979 0.1939550000000025 F F F
0.4651476454405865 0.8624813700115981 0.2920281943630454 T T T
0.2986021067286765 0.9736595925849217 0.3940526065360220 T T T
0.7988130000000027 0.9737510000000000 0.0929190000000020 F F F
0.6321459999999988 0.7515289999999979 0.1939550000000025 F F F
0.9651476736242081 0.8624813801898680 0.2920282085101215 T T T
0.7986020926418692 0.9736595772646219 0.3940525620954221 T T T

CuPd110opt
1.00000000000000
0.0000000000000000 0.0000000000000000 12.1932480000000005
9.1449359999999995 -9.1449359999999995 0.0000000000000000
15.1790699999999994 15.1790699999999994 0.0000000000000000
Cu Pd
48 48
Selective dynamics
Direct
0.0619209999999981 0.0823329999999984 0.0931690000000032 F F F
0.0619209999999981 0.2490000000000023 0.1935799999999972 F F F
0.0619169249918131 0.0823412215099238 0.2938836063541964 T T T
0.0618915349698272 0.2490361935521917 0.3944981796238160 T T T
0.3119209999999981 0.0823329999999984 0.0931690000000032 F F F
0.3119209999999981 0.2490000000000023 0.1935799999999972 F F F
0.3119169428760836 0.0823412214068320 0.2938836127668538 T T T
0.3118915441836501 0.2490361959265348 0.3944981911221054 T T T
0.5619209999999981 0.0823329999999984 0.0931690000000032 F F F
0.5619209999999981 0.2490000000000023 0.1935799999999972 F F F
0.5619169473390143 0.0823412262675371 0.2938836048572236 T T T
0.5618915400671584 0.2490361936312777 0.3944981731080779 T T T
0.8119209999999981 0.0823329999999984 0.0931690000000032 F F F
0.8119209999999981 0.2490000000000023 0.1935799999999972 F F F
0.8119169377012586 0.0823412350731463 0.2938836015982096 T T T
0.8118915344087273 0.2490361864279865 0.3944981633620788 T T T
0.0619209999999981 0.4156669999999991 0.0931690000000032 F F F

0.0619209999999981	0.5823329999999984	0.1935799999999972	F	F	F
0.0619146922170892	0.4156189959187628	0.2938846607425870	T	T	T
0.0619177652852203	0.5823398045936049	0.3945007840120924	T	T	T
0.3119209999999981	0.4156669999999991	0.0931690000000032	F	F	F
0.3119209999999981	0.5823329999999984	0.1935799999999972	F	F	F
0.3119147174049607	0.4156190047899151	0.2938846687477393	T	T	T
0.3119177714859600	0.5823397911562725	0.3945007752814296	T	T	T
0.5619209999999981	0.4156669999999991	0.0931690000000032	F	F	F
0.5619209999999981	0.5823329999999984	0.1935799999999972	F	F	F
0.5619147066463558	0.4156189929399574	0.2938846575449792	T	T	T
0.5619177665745879	0.5823398125965810	0.3945007769950714	T	T	T
0.8119209999999981	0.4156669999999991	0.0931690000000032	F	F	F
0.8119209999999981	0.5823329999999984	0.1935799999999972	F	F	F
0.8119147004421986	0.4156189841369435	0.2938846556608073	T	T	T
0.8119177621982790	0.5823398119883638	0.3945007749231764	T	T	T
0.0619209999999981	0.7490000000000023	0.0931690000000032	F	F	F
0.0619209999999981	0.9156669999999991	0.1935799999999972	F	F	F
0.0619129417715031	0.7490300356979409	0.2938844665047842	T	T	T
0.0618954458673759	0.9156117955509603	0.3944965425872145	T	T	T
0.3119209999999981	0.7490000000000023	0.0931690000000032	F	F	F
0.3119209999999981	0.9156669999999991	0.1935799999999972	F	F	F
0.3119129540554472	0.7490300192981683	0.2938844679022474	T	T	T
0.3118954570008321	0.9156118066959503	0.3944965438928564	T	T	T
0.5619209999999981	0.7490000000000023	0.0931690000000032	F	F	F
0.5619209999999981	0.9156669999999991	0.1935799999999972	F	F	F
0.5619129781445618	0.7490300277942437	0.2938844664074678	T	T	T
0.5618954535711831	0.9156117925189403	0.3944965344914475	T	T	T
0.8119209999999981	0.7490000000000023	0.0931690000000032	F	F	F
0.8119209999999981	0.9156669999999991	0.1935799999999972	F	F	F
0.8119129501076469	0.7490300314922438	0.2938844683965989	T	T	T
0.8118954534369940	0.9156117896992333	0.3944965341541360	T	T	T
0.1869209999999981	0.2490000000000023	0.0931690000000032	F	F	F
0.1869209999999981	0.0823329999999984	0.1935799999999972	F	F	F
0.1869580827567035	0.2490202204124570	0.2937098463176132	T	T	T
0.1869051567272828	0.0823273428330265	0.3942775667680546	T	T	T
0.4369209999999981	0.2490000000000023	0.0931690000000032	F	F	F
0.4369209999999981	0.0823329999999984	0.1935799999999972	F	F	F
0.4369580503657820	0.2490202264554060	0.2937098513733079	T	T	T
0.4369051189365971	0.0823273328832418	0.3942775625780165	T	T	T
0.6869209999999981	0.2490000000000023	0.0931690000000032	F	F	F
0.6869209999999981	0.0823329999999984	0.1935799999999972	F	F	F
0.6869580860390544	0.2490202321250832	0.2937098498943225	T	T	T
0.6869051386172041	0.0823273663344834	0.3942775808506598	T	T	T
0.9369209999999981	0.2490000000000023	0.0931690000000032	F	F	F

0.9369209999999981	0.0823329999999984	0.1935799999999972	F	F	F
0.9369581018180354	0.2490202394524876	0.2937098399196437	T	T	T
0.9369051561379540	0.0823273602740815	0.3942775811755331	T	T	T
0.1869209999999981	0.5823329999999984	0.0931690000000032	F	F	F
0.1869209999999981	0.4156669999999991	0.1935799999999972	F	F	F
0.1869202966829754	0.5823446602001549	0.2937007572779289	T	T	T
0.1869594127903113	0.4156563991571953	0.3942666260815318	T	T	T
0.4369209999999981	0.5823329999999984	0.0931690000000032	F	F	F
0.4369209999999981	0.4156669999999991	0.1935799999999972	F	F	F
0.4369202072953979	0.5823446461195475	0.2937007583286735	T	T	T
0.4369593505677640	0.4156563981239965	0.3942666286892771	T	T	T
0.6869209999999981	0.5823329999999984	0.0931690000000032	F	F	F
0.6869209999999981	0.4156669999999991	0.1935799999999972	F	F	F
0.6869202643828985	0.5823446898334074	0.2937007580074943	T	T	T
0.6869593892101998	0.4156563493179890	0.3942666360178160	T	T	T
0.9369209999999981	0.5823329999999984	0.0931690000000032	F	F	F
0.9369209999999981	0.4156669999999991	0.1935799999999972	F	F	F
0.9369202851963673	0.5823446650148719	0.2937007554526117	T	T	T
0.9369593987562957	0.4156563568542902	0.3942666327009214	T	T	T
0.1869209999999981	0.9156669999999991	0.0931690000000032	F	F	F
0.1869209999999981	0.7490000000000023	0.1935799999999972	F	F	F
0.1869034525795595	0.9156840545249638	0.2936947982378699	T	T	T
0.1869021390613622	0.7489981166215953	0.3942681301586091	T	T	T
0.4369209999999981	0.9156669999999991	0.0931690000000032	F	F	F
0.4369209999999981	0.7490000000000023	0.1935799999999972	F	F	F
0.4369034164599550	0.9156840715055630	0.2936947978996459	T	T	T
0.4369021041028944	0.7489981268963719	0.3942681276865407	T	T	T
0.6869209999999981	0.9156669999999991	0.0931690000000032	F	F	F
0.6869209999999981	0.7490000000000023	0.1935799999999972	F	F	F
0.6869034155797166	0.9156840157261666	0.2936947920722336	T	T	T
0.6869021330570354	0.7489981410483179	0.3942681263081791	T	T	T
0.9369209999999981	0.9156669999999991	0.0931690000000032	F	F	F
0.9369209999999981	0.7490000000000023	0.1935799999999972	F	F	F
0.9369034473126786	0.9156840294785716	0.2936947986682743	T	T	T
0.9369021357233768	0.7489981378506033	0.3942681253579262	T	T	T

Ag3Pd111opt

1.00000000000000

8.2725930000000005	-8.2725930000000005	0.0000000000000000
0.0000000000000000	12.4088899999999995	-12.4088899999999995
12.7965509999999991	12.7965509999999991	12.7965509999999991

Pd	Ag
24	72

Selective dynamics

Direct

0.3686119999999988	0.2446759999999983	0.0902349999999998	F	F	F
0.2019450000000020	0.0224540000000033	0.1979800000000012	F	F	F
0.0347397364115776	0.1333837601738283	0.3041084023893265	T	T	T
0.3683695846524033	0.2446369595554419	0.4067383589901671	T	T	T
0.8686119999999988	0.2446759999999983	0.0902349999999998	F	F	F
0.7019450000000020	0.0224540000000033	0.1979800000000012	F	F	F
0.5347397312304190	0.1333837445640250	0.3041084194867969	T	T	T
0.8683695933369298	0.2446369731434350	0.4067383570075337	T	T	T
0.3686119999999988	0.5780099999999990	0.0902349999999998	F	F	F
0.2019450000000020	0.3557869999999994	0.1979800000000012	F	F	F
0.0347396346530025	0.4667169528382971	0.3041084188333899	T	T	T
0.3683692593587756	0.5779701797892386	0.4067382698838515	T	T	T
0.8686119999999988	0.5780099999999990	0.0902349999999998	F	F	F
0.7019450000000020	0.3557869999999994	0.1979800000000012	F	F	F
0.5347396046005074	0.4667169504015590	0.3041084165297985	T	T	T
0.8683692445926289	0.5779701603403125	0.4067382703946340	T	T	T
0.3686119999999988	0.9113430000000022	0.0902349999999998	F	F	F
0.2019450000000020	0.6891210000000001	0.1979800000000012	F	F	F
0.0347388550519601	0.8000503106751350	0.3041086705715136	T	T	T
0.3683692910019044	0.9113035188194376	0.4067383460911591	T	T	T
0.8686119999999988	0.9113430000000022	0.0902349999999998	F	F	F
0.7019450000000020	0.6891210000000001	0.1979800000000012	F	F	F
0.5347388882109452	0.8000503085551042	0.3041086724555848	T	T	T
0.8683689570385426	0.9113029721513060	0.4067377280004176	T	T	T
0.3686119999999988	0.0780099999999990	0.0902349999999998	F	F	F
0.2019450000000020	0.1891210000000001	0.1979800000000012	F	F	F
0.0365465764787591	0.3006374525638990	0.3038294874574780	T	T	T
0.3693586559982420	0.0782229530721339	0.4134567937332032	T	T	T
0.1186119999999988	0.0780099999999990	0.0902349999999998	F	F	F
0.4519450000000020	0.1891210000000001	0.1979800000000012	F	F	F
0.2841189822849221	0.3005003704632955	0.3038022421475630	T	T	T
0.1180401537601298	0.0782326914136697	0.4134751810262829	T	T	T
0.1186119999999988	0.2446759999999983	0.0902349999999998	F	F	F
0.4519450000000020	0.0224540000000033	0.1979800000000012	F	F	F
0.2840857079925453	0.1323605848537717	0.3038680199459413	T	T	T
0.1179788982189630	0.2439974925637330	0.4134444290983323	T	T	T
0.8686119999999988	0.0780099999999990	0.0902349999999998	F	F	F
0.7019450000000020	0.1891210000000001	0.1979800000000012	F	F	F
0.5365465714101568	0.3006374497228865	0.3038294921394681	T	T	T
0.8693586523389188	0.0782229283969912	0.4134567848607779	T	T	T
0.6186119999999988	0.0780099999999990	0.0902349999999998	F	F	F
0.9519450000000020	0.1891210000000001	0.1979800000000012	F	F	F
0.7841189805929346	0.3005003705893193	0.3038022389271387	T	T	T

0.6180401756999322	0.0782327106725991	0.4134751883785828	T	T	T
0.6186119999999988	0.2446759999999983	0.0902349999999998	F	F	F
0.9519450000000020	0.0224540000000033	0.1979800000000012	F	F	F
0.7840856971759578	0.1323605815568167	0.3038680315754136	T	T	T
0.6179789017935110	0.2439974900544395	0.4134444408875159	T	T	T
0.3686119999999988	0.4113430000000022	0.0902349999999998	F	F	F
0.2019450000000020	0.5224540000000033	0.1979800000000012	F	F	F
0.0365462972144410	0.6339707455203631	0.3038293463412848	T	T	T
0.3693587102297917	0.4115563979852624	0.4134567818775329	T	T	T
0.1186119999999988	0.4113430000000022	0.0902349999999998	F	F	F
0.4519450000000020	0.5224540000000033	0.1979800000000012	F	F	F
0.2841187111615776	0.6338336556696258	0.3038020982277148	T	T	T
0.1180403240132504	0.4115662039527799	0.4134751830483543	T	T	T
0.1186119999999988	0.5780099999999990	0.0902349999999998	F	F	F
0.4519450000000020	0.3557869999999994	0.1979800000000012	F	F	F
0.2840856229885287	0.4656938005841593	0.3038680288854016	T	T	T
0.1179786005464254	0.5773306904726722	0.4134444316816396	T	T	T
0.8686119999999988	0.4113430000000022	0.0902349999999998	F	F	F
0.7019450000000020	0.5224540000000033	0.1979800000000012	F	F	F
0.5365448834362817	0.6339697600393979	0.3038293981818027	T	T	T
0.8693587275754621	0.4115563988607273	0.4134567902803160	T	T	T
0.6186119999999988	0.4113430000000022	0.0902349999999998	F	F	F
0.9519450000000020	0.5224540000000033	0.1979800000000012	F	F	F
0.7841189321784862	0.6338335529897001	0.3038021541868814	T	T	T
0.6180403088840792	0.4115661932426817	0.4134752026804294	T	T	T
0.6186119999999988	0.5780099999999990	0.0902349999999998	F	F	F
0.9519450000000020	0.3557869999999994	0.1979800000000012	F	F	F
0.7840856060746478	0.4656937946869008	0.3038680407584046	T	T	T
0.6179785554930172	0.5773306851551044	0.4134444221954124	T	T	T
0.3686119999999988	0.7446759999999983	0.0902349999999998	F	F	F
0.2019450000000020	0.8557869999999994	0.1979800000000012	F	F	F
0.0365451980922206	0.9673032190546927	0.3038293693042985	T	T	T
0.3693588242481227	0.7448896499683507	0.4134568524035189	T	T	T
0.1186119999999988	0.7446759999999983	0.0902349999999998	F	F	F
0.4519450000000020	0.8557869999999994	0.1979800000000012	F	F	F
0.2841186229163130	0.9671668845077364	0.3038020832157961	T	T	T
0.1180404085162446	0.7448994522730162	0.4134752358917089	T	T	T
0.1186119999999988	0.9113430000000022	0.0902349999999998	F	F	F
0.4519450000000020	0.6891210000000001	0.1979800000000012	F	F	F
0.2840857908505673	0.7990271594144566	0.3038681895263899	T	T	T
0.1179786741074361	0.9106641013692978	0.4134444168280529	T	T	T
0.8686119999999988	0.7446759999999983	0.0902349999999998	F	F	F
0.7019450000000020	0.8557869999999994	0.1979800000000012	F	F	F
0.5365462804587264	0.9673039669640049	0.3038293420598772	T	T	T

0.8693587227035608	0.7448899005787034	0.4134565325834216	T	T	T
0.6186119999999988	0.7446759999999983	0.0902349999999998	F	F	F
0.9519450000000020	0.8557869999999994	0.1979800000000012	F	F	F
0.7841188400457240	0.9671668361194005	0.3038021313791842	T	T	T
0.6180404151886298	0.7448994697709829	0.4134753836106098	T	T	T
0.6186119999999988	0.9113430000000022	0.0902349999999998	F	F	F
0.9519450000000020	0.6891210000000001	0.1979800000000012	F	F	F
0.7840774399324190	0.7990237863858151	0.3038664929283463	T	T	T
0.6179786741169337	0.9106640953474303	0.4134444268009541	T	T	T

AgPd3111opt

1.00000000000000

8.0508539999999993 -8.0508539999999993 0.0000000000000000

0.0000000000000000 12.0762809999999998 -12.0762809999999998

12.6856810000000007 12.6856810000000007 12.6856810000000007

Pd Ag

72 24

Selective dynamics

Direct

0.1186119999999988	0.2446759999999983	0.0910239999999973	F	F	F
0.4519450000000020	0.0224540000000033	0.1967969999999966	F	F	F
0.2861714401726316	0.1348206658531085	0.3004880746763189	T	T	T
0.1192245731573567	0.2453716357831540	0.4031867254910355	T	T	T
0.1186119999999988	0.0780099999999990	0.0910239999999973	F	F	F
0.4519450000000020	0.1891210000000001	0.1967969999999966	F	F	F
0.2859433785808515	0.3000722119687068	0.3004694620324567	T	T	T
0.1194953658039524	0.0777989713318661	0.4031282220149502	T	T	T
0.3686119999999988	0.0780099999999990	0.0910239999999973	F	F	F
0.2019450000000020	0.1891210000000001	0.1967969999999966	F	F	F
0.0341177221392443	0.2999370790785055	0.3004764596505549	T	T	T
0.3677011014616339	0.0778236992783802	0.4030262990358741	T	T	T
0.6186119999999988	0.2446759999999983	0.0910239999999973	F	F	F
0.9519450000000020	0.0224540000000033	0.1967969999999966	F	F	F
0.7861720474407387	0.1348201156350198	0.3004877880984748	T	T	T
0.6192275986725161	0.2453721015508362	0.4031887608551247	T	T	T
0.6186119999999988	0.0780099999999990	0.0910239999999973	F	F	F
0.9519450000000020	0.1891210000000001	0.1967969999999966	F	F	F
0.7859443833080996	0.3000729132569712	0.3004691705545977	T	T	T
0.6194995709794490	0.0778014809769557	0.4031304690265473	T	T	T
0.8686119999999988	0.0780099999999990	0.0910239999999973	F	F	F
0.7019450000000020	0.1891210000000001	0.1967969999999966	F	F	F
0.5341172827760462	0.2999372849750133	0.3004768420776431	T	T	T
0.8676997181826398	0.0778215978826356	0.4030226470435917	T	T	T
0.1186119999999988	0.5780099999999990	0.0910239999999973	F	F	F

0.4519450000000020	0.3557869999999994	0.1967969999999966	F	F	F
0.2861726449712520	0.4681551232864029	0.3004887882273614	T	T	T
0.1192233893957376	0.5787033372678939	0.4031885608812560	T	T	T
0.1186119999999988	0.4113430000000022	0.0910239999999973	F	F	F
0.4519450000000020	0.5224540000000033	0.1967969999999966	F	F	F
0.2859428425999813	0.6334043605612333	0.3004692104908491	T	T	T
0.1194959147655304	0.4111317194575713	0.4031287005815292	T	T	T
0.3686119999999988	0.4113430000000022	0.0910239999999973	F	F	F
0.2019450000000020	0.5224540000000033	0.1967969999999966	F	F	F
0.0341164362322008	0.6332692356491321	0.3004762885143297	T	T	T
0.3676995007988847	0.4111551550240897	0.4030251238446784	T	T	T
0.6186119999999988	0.5780099999999990	0.0910239999999973	F	F	F
0.9519450000000020	0.3557869999999994	0.1967969999999966	F	F	F
0.7861727612191053	0.4681544236887299	0.3004880052349733	T	T	T
0.6192265546431630	0.5787039811091703	0.4031900390922341	T	T	T
0.6186119999999988	0.4113430000000022	0.0910239999999973	F	F	F
0.9519450000000020	0.5224540000000033	0.1967969999999966	F	F	F
0.7859424169082374	0.6334049803929410	0.3004692356277751	T	T	T
0.6194999299812985	0.4111335105355384	0.4031289988530656	T	T	T
0.8686119999999988	0.4113430000000022	0.0910239999999973	F	F	F
0.7019450000000020	0.5224540000000033	0.1967969999999966	F	F	F
0.5341156739311135	0.6332690577036093	0.3004768461864149	T	T	T
0.8676982743469543	0.4111554454262231	0.4030232491400147	T	T	T
0.1186119999999988	0.9113430000000022	0.0910239999999973	F	F	F
0.4519450000000020	0.6891210000000001	0.1967969999999966	F	F	F
0.2861723512123553	0.8014875519396256	0.3004881254667839	T	T	T
0.1192227477561193	0.9120380198232935	0.4031888274044642	T	T	T
0.1186119999999988	0.7446759999999983	0.0910239999999973	F	F	F
0.4519450000000020	0.8557869999999994	0.1967969999999966	F	F	F
0.2859446651789432	0.9667391653271333	0.3004686742139869	T	T	T
0.1194956211287179	0.7444667235955276	0.4031310574632475	T	T	T
0.3686119999999988	0.7446759999999983	0.0910239999999973	F	F	F
0.2019450000000020	0.8557869999999994	0.1967969999999966	F	F	F
0.0341176543409755	0.9666050715450629	0.3004750419187667	T	T	T
0.3677028774964007	0.7444905674701109	0.4030278904956952	T	T	T
0.6186119999999988	0.9113430000000022	0.0910239999999973	F	F	F
0.9519450000000020	0.6891210000000001	0.1967969999999966	F	F	F
0.7861721258298529	0.8014867007754227	0.3004874448514566	T	T	T
0.6192293531910963	0.9120387358951746	0.4031892193260068	T	T	T
0.6186119999999988	0.7446759999999983	0.0910239999999973	F	F	F
0.9519450000000020	0.8557869999999994	0.1967969999999966	F	F	F
0.7859454652184887	0.9667405671364516	0.3004672450821213	T	T	T
0.6195007880939545	0.7444693732887511	0.4031325580574365	T	T	T
0.8686119999999988	0.7446759999999983	0.0910239999999973	F	F	F

0.7019450000000020 0.8557869999999994 0.1967969999999966 F F F
0.5341163576376093 0.9666038410698462 0.3004756916072551 T T T
0.8677008547417577 0.7444907687250114 0.4030261402017142 T T T
0.3686119999999988 0.2446759999999983 0.0910239999999973 F F F
0.2019450000000020 0.0224540000000033 0.1967969999999966 F F F
0.0352089848628973 0.1337045131560803 0.2997507825482291 T T T
0.3689663035981998 0.2449247851450515 0.4116850270689860 T T T
0.8686119999999988 0.2446759999999983 0.0910239999999973 F F F
0.7019450000000020 0.0224540000000033 0.1967969999999966 F F F
0.5352014918797365 0.1337005441569539 0.2997501275766808 T T T
0.8689644477522389 0.2449245660961662 0.4116860285821735 T T T
0.3686119999999988 0.5780099999999990 0.0910239999999973 F F F
0.2019450000000020 0.3557869999999994 0.1967969999999966 F F F
0.0352107929437255 0.4670389107578247 0.2997497535560443 T T T
0.3689666817949200 0.5782596512036503 0.4116844697304459 T T T
0.8686119999999988 0.5780099999999990 0.0910239999999973 F F F
0.7019450000000020 0.3557869999999994 0.1967969999999966 F F F
0.5352033398089380 0.4670372949405799 0.2997496977734450 T T T
0.8689661328599556 0.5782593833108065 0.4116854731379445 T T T
0.3686119999999988 0.9113430000000022 0.0910239999999973 F F F
0.2019450000000020 0.6891210000000001 0.1967969999999966 F F F
0.0352097071854229 0.8003690293482458 0.2997504379258671 T T T
0.3689652802839307 0.9115909757678373 0.4116848736213606 T T T
0.8686119999999988 0.9113430000000022 0.0910239999999973 F F F
0.7019450000000020 0.6891210000000001 0.1967969999999966 F F F
0.5351992058486926 0.8003663727568709 0.2997501960316480 T T T
0.8689642670137974 0.9115903479163276 0.4116867888217521 T T T

Au3Pd111opt
1.00000000000000
8.2808240000000009 -8.2808240000000009 0.0000000000000000
0.0000000000000000 12.4212360000000004 -12.4212360000000004
12.8006659999999997 12.8006659999999997 12.8006659999999997
Pd Au
24 72
Selective dynamics
Direct
0.3686119999999988 0.2446759999999983 0.0902060000000020 F F F
0.2019450000000020 0.0224540000000033 0.1980239999999966 F F F
0.0349455416586126 0.1333104433706852 0.3038240592143085 T T T
0.3683059265623347 0.2444131773121967 0.4094962974236351 T T T
0.8686119999999988 0.2446759999999983 0.0902060000000020 F F F
0.7019450000000020 0.0224540000000033 0.1980239999999966 F F F
0.5349455187707496 0.1333104445206552 0.3038240551837892 T T T

0.8683059504851588	0.2444131772426800	0.4094963083204742	T	T	T
0.3686119999999988	0.5780099999999990	0.0902060000000020	F	F	F
0.2019450000000020	0.3557869999999994	0.1980239999999966	F	F	F
0.0349454016567149	0.4666440453279911	0.3038245617732218	T	T	T
0.3683055343341863	0.5777463956961691	0.4094983496638214	T	T	T
0.8686119999999988	0.5780099999999990	0.0902060000000020	F	F	F
0.7019450000000020	0.3557869999999994	0.1980239999999966	F	F	F
0.5349454271054370	0.4666440593799133	0.3038245490172855	T	T	T
0.8683054781513604	0.5777464019192354	0.4094983718885078	T	T	T
0.3686119999999988	0.9113430000000022	0.0902060000000020	F	F	F
0.2019450000000020	0.6891210000000001	0.1980239999999966	F	F	F
0.0349452782767533	0.7999767636780709	0.3038246053098439	T	T	T
0.3683054412101028	0.9110794492672551	0.4094973159810579	T	T	T
0.8686119999999988	0.9113430000000022	0.0902060000000020	F	F	F
0.7019450000000020	0.6891210000000001	0.1980239999999966	F	F	F
0.5349452960514648	0.7999767612937456	0.3038246246095231	T	T	T
0.8683054962243191	0.9110794422644208	0.4094973321398194	T	T	T
0.3686119999999988	0.0780099999999990	0.0902060000000020	F	F	F
0.2019450000000020	0.1891210000000001	0.1980239999999966	F	F	F
0.0361887603736206	0.3004835747111967	0.3043067470230033	T	T	T
0.3695390645292276	0.0782810619169309	0.4145289099561070	T	T	T
0.1186119999999988	0.0780099999999990	0.0902060000000020	F	F	F
0.4519450000000020	0.1891210000000001	0.1980239999999966	F	F	F
0.2845129925525142	0.3004684382753959	0.3042711872677524	T	T	T
0.1179267427252459	0.0782332934879347	0.4146006170447048	T	T	T
0.1186119999999988	0.2446759999999983	0.0902060000000020	F	F	F
0.4519450000000020	0.0224540000000033	0.1980239999999966	F	F	F
0.2844467922294950	0.1326330777140836	0.3042957963182393	T	T	T
0.1177267266234421	0.2436233687040074	0.4144071518623289	T	T	T
0.8686119999999988	0.0780099999999990	0.0902060000000020	F	F	F
0.7019450000000020	0.1891210000000001	0.1980239999999966	F	F	F
0.5361887400277868	0.3004835855440609	0.3043067294903646	T	T	T
0.8695391462344012	0.0782810643293288	0.4145288823743480	T	T	T
0.6186119999999988	0.0780099999999990	0.0902060000000020	F	F	F
0.9519450000000020	0.1891210000000001	0.1980239999999966	F	F	F
0.7845129572480570	0.3004684327807444	0.3042712166238649	T	T	T
0.6179266757804249	0.0782332914287868	0.4146006069181933	T	T	T
0.6186119999999988	0.2446759999999983	0.0902060000000020	F	F	F
0.9519450000000020	0.0224540000000033	0.1980239999999966	F	F	F
0.7844468180813992	0.1326330794910228	0.3042958265585909	T	T	T
0.6177266377243464	0.2436233824054180	0.4144071603626591	T	T	T
0.3686119999999988	0.4113430000000022	0.0902060000000020	F	F	F
0.2019450000000020	0.5224540000000033	0.1980239999999966	F	F	F
0.0361887190854980	0.6338170222554891	0.3043064754335374	T	T	T

0.3695389054243141	0.4116134360605342	0.4145292074348786	T	T	T
0.1186119999999988	0.4113430000000022	0.0902060000000020	F	F	F
0.4519450000000020	0.5224540000000033	0.1980239999999966	F	F	F
0.2845130261979631	0.6338019081485901	0.3042709888926712	T	T	T
0.1179257549385276	0.4115656951423058	0.4146008448586745	T	T	T
0.1186119999999988	0.5780099999999990	0.0902060000000020	F	F	F
0.4519450000000020	0.3557869999999994	0.1980239999999966	F	F	F
0.2844458401645957	0.4659652687374664	0.3042956951341772	T	T	T
0.1177264507091275	0.5769567638060190	0.4144080584263855	T	T	T
0.8686119999999988	0.4113430000000022	0.0902060000000020	F	F	F
0.7019450000000020	0.5224540000000033	0.1980239999999966	F	F	F
0.5361887513380069	0.6338170325600058	0.3043064633256546	T	T	T
0.8695388959117651	0.4116134607629352	0.4145291594999572	T	T	T
0.6186119999999988	0.4113430000000022	0.0902060000000020	F	F	F
0.9519450000000020	0.5224540000000033	0.1980239999999966	F	F	F
0.7845130201542817	0.6338018948265948	0.3042709811672434	T	T	T
0.6179256591498768	0.4115656626074466	0.4146008668757802	T	T	T
0.6186119999999988	0.5780099999999990	0.0902060000000020	F	F	F
0.9519450000000020	0.3557869999999994	0.1980239999999966	F	F	F
0.7844457952670862	0.4659652764942115	0.3042957031269554	T	T	T
0.6177264195607330	0.5769567481276211	0.4144080459129814	T	T	T
0.3686119999999988	0.7446759999999983	0.0902060000000020	F	F	F
0.2019450000000020	0.8557869999999994	0.1980239999999966	F	F	F
0.0361889593513803	0.9671511094530374	0.3043065321961512	T	T	T
0.3695389397616250	0.7449478109718541	0.4145296946475732	T	T	T
0.1186119999999988	0.7446759999999983	0.0902060000000020	F	F	F
0.4519450000000020	0.8557869999999994	0.1980239999999966	F	F	F
0.2845134438121481	0.9671359310991823	0.3042710287603633	T	T	T
0.1179277049769077	0.7449000317523992	0.4146012816851692	T	T	T
0.1186119999999988	0.9113430000000022	0.0902060000000020	F	F	F
0.4519450000000020	0.6891210000000001	0.1980239999999966	F	F	F
0.2844469950242497	0.7992997907607124	0.3042959873040719	T	T	T
0.1177265513623207	0.9102902647220515	0.4144074944759111	T	T	T
0.8686119999999988	0.7446759999999983	0.0902060000000020	F	F	F
0.7019450000000020	0.8557869999999994	0.1980239999999966	F	F	F
0.5361889387040865	0.9671510923626321	0.3043065545096496	T	T	T
0.8695389363064403	0.7449478208681019	0.4145296496161073	T	T	T
0.6186119999999988	0.7446759999999983	0.0902060000000020	F	F	F
0.9519450000000020	0.8557869999999994	0.1980239999999966	F	F	F
0.7845134641308315	0.9671359055093854	0.3042710333442651	T	T	T
0.6179277216159825	0.7449000107391380	0.4146012924918921	T	T	T
0.6186119999999988	0.9113430000000022	0.0902060000000020	F	F	F
0.9519450000000020	0.6891210000000001	0.1980239999999966	F	F	F
0.7844470089042873	0.7992997883060944	0.3042959677656220	T	T	T

0.6177265589262581 0.9102902407587042 0.4144075231919481 T T T

AuPd3111opt
1.00000000000000
8.0908899999999999 -8.0908899999999999 0.0000000000000000
0.0000000000000000 12.1363350000000008 -12.1363350000000008
12.7056989999999992 12.7056989999999992 12.7056989999999992
Pd Au
72 24
Selective dynamics
Direct

0.1202090000000027	0.2436119999999988	0.0908810000000031	F	F	F
0.4535419999999988	0.0213889999999992	0.1970129999999983	F	F	F
0.2876914557804910	0.1336244528295060	0.2998862445674027	T	T	T
0.1212149397717653	0.2446995924963179	0.4031801571250000	T	T	T
0.1202090000000027	0.0769450000000020	0.0908810000000031	F	F	F
0.4535419999999988	0.1880560000000031	0.1970129999999983	F	F	F
0.2874522427536135	0.2989003109246109	0.2997795585570676	T	T	T
0.1214892753048376	0.0765492433857871	0.4032007241727706	T	T	T
0.3702090000000027	0.0769450000000020	0.0908810000000031	F	F	F
0.2035419999999988	0.1880560000000031	0.1970129999999983	F	F	F
0.0362615896621427	0.2990829750149376	0.2998049648270406	T	T	T
0.3685346369524900	0.0764626843155604	0.4029774849647654	T	T	T
0.6202090000000027	0.2436119999999988	0.0908810000000031	F	F	F
0.9535419999999988	0.0213889999999992	0.1970129999999983	F	F	F
0.7876963492176919	0.1336249131834404	0.2998879730069923	T	T	T
0.6212116555920950	0.2446956178308770	0.4031887556438761	T	T	T
0.6202090000000027	0.0769450000000020	0.0908810000000031	F	F	F
0.9535419999999988	0.1880560000000031	0.1970129999999983	F	F	F
0.7874571217401884	0.2989029941660917	0.2997829328707524	T	T	T
0.6214903489651706	0.0765526510099678	0.4032076043829316	T	T	T
0.8702090000000027	0.0769450000000020	0.0908810000000031	F	F	F
0.7035419999999988	0.1880560000000031	0.1970129999999983	F	F	F
0.5362595066948964	0.2990821596462059	0.2998079968000700	T	T	T
0.8685429194892621	0.0764640422207150	0.4029797582694527	T	T	T
0.1202090000000027	0.5769450000000020	0.0908810000000031	F	F	F
0.4535419999999988	0.3547229999999999	0.1970129999999983	F	F	F
0.2876883121551503	0.4669533263272561	0.2998879409870960	T	T	T
0.1212017701130956	0.5780241191181810	0.4031812350747567	T	T	T
0.1202090000000027	0.4102779999999981	0.0908810000000031	F	F	F
0.4535419999999988	0.5213889999999992	0.1970129999999983	F	F	F
0.2874469609202204	0.6322330994580716	0.2997860715608897	T	T	T
0.1214874926306262	0.4098748782094079	0.4031950558854091	T	T	T
0.3702090000000027	0.4102779999999981	0.0908810000000031	F	F	F

0.2035419999999988	0.5213889999999992	0.1970129999999983	F	F	F
0.0362665000630090	0.6324164708220599	0.2998114142901385	T	T	T
0.3685267186764686	0.4097873153754206	0.4029691273703003	T	T	T
0.6202090000000027	0.5769450000000020	0.0908810000000031	F	F	F
0.9535419999999988	0.3547229999999999	0.1970129999999983	F	F	F
0.7876917785544544	0.4669535852697326	0.2998909478286992	T	T	T
0.6212000168557780	0.5780223562613885	0.4031833390752726	T	T	T
0.6202090000000027	0.4102779999999981	0.0908810000000031	F	F	F
0.9535419999999988	0.5213889999999992	0.1970129999999983	F	F	F
0.7874527664322544	0.6322358706178556	0.2997862098949808	T	T	T
0.6214840170837965	0.4098783393966252	0.4032022142134460	T	T	T
0.8702090000000027	0.4102779999999981	0.0908810000000031	F	F	F
0.7035419999999988	0.5213889999999992	0.1970129999999983	F	F	F
0.5362631862375058	0.6324147123612923	0.2998116751418363	T	T	T
0.8685365926941485	0.4097910309342738	0.4029773612742772	T	T	T
0.1202090000000027	0.9102779999999981	0.0908810000000031	F	F	F
0.4535419999999988	0.6880560000000031	0.1970129999999983	F	F	F
0.2876857445707619	0.8002841435263364	0.2998918477824259	T	T	T
0.1211996924268955	0.9113595257262338	0.4031822016183129	T	T	T
0.1202090000000027	0.7436119999999988	0.0908810000000031	F	F	F
0.4535419999999988	0.8547229999999999	0.1970129999999983	F	F	F
0.2874469944351263	0.9655662048012019	0.2997828135528748	T	T	T
0.1214861235194099	0.7432110267565702	0.4032052596543068	T	T	T
0.3702090000000027	0.7436119999999988	0.0908810000000031	F	F	F
0.2035419999999988	0.8547229999999999	0.1970129999999983	F	F	F
0.0362645749054428	0.9657475444261643	0.2998062988928250	T	T	T
0.3685312731008580	0.7431232813136537	0.4029790494277738	T	T	T
0.6202090000000027	0.9102779999999981	0.0908810000000031	F	F	F
0.9535419999999988	0.6880560000000031	0.1970129999999983	F	F	F
0.7876924392056824	0.8002868350283685	0.2998909863873686	T	T	T
0.6212142615019792	0.9113604387053794	0.4031937921473494	T	T	T
0.6202090000000027	0.7436119999999988	0.0908810000000031	F	F	F
0.9535419999999988	0.8547229999999999	0.1970129999999983	F	F	F
0.7874521530246579	0.9655674101876464	0.2997827417015003	T	T	T
0.6214875407107020	0.7432132997701987	0.4032056608470297	T	T	T
0.8702090000000027	0.7436119999999988	0.0908810000000031	F	F	F
0.7035419999999988	0.8547229999999999	0.1970129999999983	F	F	F
0.5362642188243343	0.9657484345051798	0.2998094444611418	T	T	T
0.8685352767418950	0.7431254788662114	0.4029804702239852	T	T	T
0.3702090000000027	0.2436119999999988	0.0908810000000031	F	F	F
0.2035419999999988	0.0213889999999992	0.1970129999999983	F	F	F
0.0372912661200887	0.1328633283107180	0.2996976008625969	T	T	T
0.3706579456477692	0.2438671332302003	0.4084117478924295	T	T	T
0.8702090000000027	0.2436119999999988	0.0908810000000031	F	F	F

0.7035419999999988 0.0213889999999992 0.1970129999999983 F F F
0.5372926583598387 0.1328637550697368 0.2996987602844395 T T T
0.8706582223864178 0.2438671166872275 0.4084113260642327 T T T
0.3702090000000027 0.5769450000000020 0.0908810000000031 F F F
0.2035419999999988 0.3547229999999999 0.1970129999999983 F F F
0.0372915134704235 0.4661976577827873 0.2997014640129968 T T T
0.3706564596381130 0.5771982651357347 0.4084119238053535 T T T
0.8702090000000027 0.5769450000000020 0.0908810000000031 F F F
0.7035419999999988 0.3547229999999999 0.1970129999999983 F F F
0.5372936616953869 0.4661971366056710 0.2997004110365554 T T T
0.8706487496075683 0.5772073915515020 0.4084182604626895 T T T
0.3702090000000027 0.9102779999999981 0.0908810000000031 F F F
0.2035419999999988 0.6880560000000031 0.1970129999999983 F F F
0.0372922309158077 0.7995293073932571 0.2997040293774740 T T T
0.3706518590495252 0.9105436600674504 0.4084175900911265 T T T
0.8702090000000027 0.9102779999999981 0.0908810000000031 F F F
0.7035419999999988 0.6880560000000031 0.1970129999999983 F F F
0.5372898612104350 0.7995294424027670 0.2997030169377922 T T T
0.8706581010398587 0.9105340947245223 0.4084111010401658 T T T

PdH_Hopt
1.00000000000000
8.3218779999999999 -8.3218779999999999 0.0000000000000000
0.0000000000000000 12.4828170000000007 -12.4828170000000007
14.2081730000000004 14.2081730000000004 14.2081730000000004
H Pd
120 96
Selective dynamics
Direct
0.2833329999999989 0.2444440000000014 0.0812700000000035 F F F
0.1166669999999996 0.0222219999999993 0.1788889999999981 F F F
0.4500000000000028 0.1333330000000004 0.2765070000000023 F F F
0.2825952256699655 0.2443162448891312 0.3768849103491335 T T T
0.1170580411368594 0.0226283770582149 0.4528501404613397 T T T
0.2833329999999989 0.0777780000000021 0.0812700000000035 F F F
0.1166669999999996 0.1888890000000032 0.1788889999999981 F F F
0.4500000000000028 0.2999999999999972 0.2765070000000023 F F F
0.2825934994124092 0.0776479386778413 0.3768805734471418 T T T
0.1170580425592978 0.1892947869994601 0.4528506170678060 T T T
0.0333329999999989 0.0777780000000021 0.0812700000000035 F F F
0.3666669999999996 0.1888890000000032 0.1788889999999981 F F F
0.2000000000000028 0.2999999999999972 0.2765070000000023 F F F
0.0325933136000815 0.0776478197683720 0.3768810495631545 T T T
0.3670582666441348 0.1892947346286254 0.4528507522487466 T T T

0.0333329999999989	0.2444440000000014	0.0812700000000035	F	F	F
0.3666669999999996	0.0222219999999993	0.1788889999999981	F	F	F
0.2000000000000028	0.1333330000000004	0.2765070000000023	F	F	F
0.0325960907289426	0.2443163276490156	0.3768852049176447	T	T	T
0.3670580209179616	0.0226283221644876	0.4528500566262379	T	T	T
0.7833329999999989	0.2444440000000014	0.0812700000000035	F	F	F
0.6166669999999996	0.0222219999999993	0.1788889999999981	F	F	F
0.9500000000000028	0.1333330000000004	0.2765070000000023	F	F	F
0.7825952097560432	0.2443159096236746	0.3768853164834705	T	T	T
0.6170577414760029	0.0226281840362150	0.4528500462879526	T	T	T
0.7833329999999989	0.0777780000000021	0.0812700000000035	F	F	F
0.6166669999999996	0.1888890000000032	0.1788889999999981	F	F	F
0.9500000000000028	0.2999999999999972	0.2765070000000023	F	F	F
0.7825933956032876	0.0776480146996785	0.3768812861018680	T	T	T
0.6170579344708308	0.1892949992990580	0.4528507114621699	T	T	T
0.5333329999999989	0.0777780000000021	0.0812700000000035	F	F	F
0.8666669999999996	0.1888890000000032	0.1788889999999981	F	F	F
0.7000000000000028	0.2999999999999972	0.2765070000000023	F	F	F
0.5325923869098037	0.0776471456271540	0.3768814080846366	T	T	T
0.8670580219425854	0.1892948066503608	0.4528506669178705	T	T	T
0.5333329999999989	0.2444440000000014	0.0812700000000035	F	F	F
0.8666669999999996	0.0222219999999993	0.1788889999999981	F	F	F
0.7000000000000028	0.1333330000000004	0.2765070000000023	F	F	F
0.5325941459893492	0.2443158213496734	0.3768850066650307	T	T	T
0.8670583167760759	0.0226282416531733	0.4528501660975641	T	T	T
0.2833329999999989	0.5777780000000021	0.0812700000000035	F	F	F
0.1166669999999996	0.3555560000000000	0.1788889999999981	F	F	F
0.4500000000000028	0.4666670000000011	0.2765070000000023	F	F	F
0.2826025620063822	0.5776599290153485	0.3768822320148018	T	T	T
0.1170609798247683	0.3559648135994652	0.4528511451150100	T	T	T
0.2833329999999989	0.4111109999999982	0.0812700000000035	F	F	F
0.1166669999999996	0.5222219999999993	0.1788889999999981	F	F	F
0.4500000000000028	0.6333330000000004	0.2765070000000023	F	F	F
0.2825990923412444	0.4109879592725035	0.3768854223052100	T	T	T
0.1170634852926938	0.5226352298580331	0.4528508999218255	T	T	T
0.0333329999999989	0.4111109999999982	0.0812700000000035	F	F	F
0.3666669999999996	0.5222219999999993	0.1788889999999981	F	F	F
0.2000000000000028	0.6333330000000004	0.2765070000000023	F	F	F
0.0325998337744453	0.4109890152431385	0.3768856140538424	T	T	T
0.3670638159018388	0.5226353940247530	0.4528509415523105	T	T	T
0.0333329999999989	0.5777780000000021	0.0812700000000035	F	F	F
0.3666669999999996	0.3555560000000000	0.1788889999999981	F	F	F
0.2000000000000028	0.4666670000000011	0.2765070000000023	F	F	F
0.0326025384942957	0.5776595353288427	0.3768823356254797	T	T	T

0.3670605342782333	0.3559648958681379	0.4528509109581048	T	T	T
0.7833329999999989	0.5777780000000021	0.0812700000000035	F	F	F
0.6166669999999996	0.3555560000000000	0.1788889999999981	F	F	F
0.9500000000000028	0.4666670000000011	0.2765070000000023	F	F	F
0.7826014241196160	0.5776593153368662	0.3768820116093264	T	T	T
0.6170604471218613	0.3559645866873468	0.4528508843500786	T	T	T
0.7833329999999989	0.4111109999999982	0.0812700000000035	F	F	F
0.6166669999999996	0.5222219999999993	0.1788889999999981	F	F	F
0.9500000000000028	0.6333330000000004	0.2765070000000023	F	F	F
0.7825998531999230	0.4109889125030978	0.3768866096511697	T	T	T
0.6170637218069053	0.5226353234326152	0.4528509611617092	T	T	T
0.5333329999999989	0.4111109999999982	0.0812700000000035	F	F	F
0.8666669999999996	0.5222219999999993	0.1788889999999981	F	F	F
0.7000000000000028	0.6333330000000004	0.2765070000000023	F	F	F
0.5325999819599239	0.4109891895154602	0.3768856906030680	T	T	T
0.8670637547271586	0.5226355870228018	0.4528507440989131	T	T	T
0.5333329999999989	0.5777780000000021	0.0812700000000035	F	F	F
0.8666669999999996	0.3555560000000000	0.1788889999999981	F	F	F
0.7000000000000028	0.4666670000000011	0.2765070000000023	F	F	F
0.5326020747530684	0.5776601374989051	0.3768817072594526	T	T	T
0.8670606377899632	0.3559647202592460	0.4528509314244580	T	T	T
0.2833329999999989	0.9111109999999982	0.0812700000000035	F	F	F
0.1166669999999996	0.6888890000000032	0.1788889999999981	F	F	F
0.4500000000000028	0.7999999999999972	0.2765070000000023	F	F	F
0.2825965971335805	0.9109844515792999	0.3768771665652306	T	T	T
0.1170635350088293	0.6893023205958992	0.4528505224872263	T	T	T
0.2833329999999989	0.7444440000000014	0.0812700000000035	F	F	F
0.1166669999999996	0.8555560000000000	0.1788889999999981	F	F	F
0.4500000000000028	0.9666670000000011	0.2765070000000023	F	F	F
0.2825996712792912	0.7443235976317729	0.3768771966194099	T	T	T
0.1170613383004561	0.8559652295342370	0.4528499299248275	T	T	T
0.0333329999999989	0.7444440000000014	0.0812700000000035	F	F	F
0.3666669999999996	0.8555560000000000	0.1788889999999981	F	F	F
0.2000000000000028	0.9666670000000011	0.2765070000000023	F	F	F
0.0326000743387841	0.7443233252396328	0.3768774083254379	T	T	T
0.3670603267460930	0.8559652411198787	0.4528500388531511	T	T	T
0.0333329999999989	0.9111109999999982	0.0812700000000035	F	F	F
0.3666669999999996	0.6888890000000032	0.1788889999999981	F	F	F
0.2000000000000028	0.7999999999999972	0.2765070000000023	F	F	F
0.0325959141613678	0.9109844517731713	0.3768772395649552	T	T	T
0.3670633945593322	0.6893018969391560	0.4528502977016288	T	T	T
0.7833329999999989	0.9111109999999982	0.0812700000000035	F	F	F
0.6166669999999996	0.6888890000000032	0.1788889999999981	F	F	F
0.9500000000000028	0.7999999999999972	0.2765070000000023	F	F	F

0.7825949715640002	0.9109838271783195	0.3768770884889314	T	T	T
0.6170635254195600	0.6893021854379197	0.4528504044175521	T	T	T
0.7833329999999989	0.7444440000000014	0.0812700000000035	F	F	F
0.6166669999999996	0.8555560000000000	0.1788889999999981	F	F	F
0.9500000000000028	0.9666670000000011	0.2765070000000023	F	F	F
0.7825996078592321	0.7443233723337246	0.3768772802398296	T	T	T
0.6170612183876931	0.8559651948569522	0.4528500777118842	T	T	T
0.5333329999999989	0.7444440000000014	0.0812700000000035	F	F	F
0.8666669999999996	0.8555560000000000	0.1788889999999981	F	F	F
0.7000000000000028	0.9666670000000011	0.2765070000000023	F	F	F
0.5326007979241117	0.7443230861771211	0.3768774940950780	T	T	T
0.8670604204962788	0.8559653318580646	0.4528499922914132	T	T	T
0.5333329999999989	0.9111109999999982	0.0812700000000035	F	F	F
0.8666669999999996	0.6888890000000032	0.1788889999999981	F	F	F
0.7000000000000028	0.7999999999999972	0.2765070000000023	F	F	F
0.5325951720016868	0.9109840874213417	0.3768768466044354	T	T	T
0.8670635760198530	0.6893018114363162	0.4528504490044172	T	T	T
0.4500000000000028	0.2999999999999972	0.1300790000000021	F	F	F
0.2833329999999989	0.0777780000000021	0.2276979999999966	F	F	F
0.1164860982247340	0.1886774947940219	0.3231236232136103	T	T	T
0.4504297185539411	0.3002526243367836	0.4223816429255574	T	T	T
0.4500000000000028	0.1333330000000004	0.1300790000000021	F	F	F
0.2833329999999989	0.2444440000000014	0.2276979999999966	F	F	F
0.1164863768223254	0.0220108163827467	0.3231231688892275	T	T	T
0.4504295515082278	0.1335854853511874	0.4223813902158564	T	T	T
0.2000000000000028	0.1333330000000004	0.1300790000000021	F	F	F
0.0333329999999989	0.2444440000000014	0.2276979999999966	F	F	F
0.3664864445296332	0.0220110255988305	0.3231229775523401	T	T	T
0.2004293234512907	0.1335855055732494	0.4223813643531220	T	T	T
0.2000000000000028	0.2999999999999972	0.1300790000000021	F	F	F
0.0333329999999989	0.0777780000000021	0.2276979999999966	F	F	F
0.3664858275670255	0.1886774829911499	0.3231239188098659	T	T	T
0.2004294938365869	0.3002525915460066	0.4223811553997077	T	T	T
0.9500000000000028	0.2999999999999972	0.1300790000000021	F	F	F
0.7833329999999989	0.0777780000000021	0.2276979999999966	F	F	F
0.6164860180409434	0.1886775784486498	0.3231237841733014	T	T	T
0.9504297069013129	0.3002526218564476	0.4223817916165635	T	T	T
0.9500000000000028	0.1333330000000004	0.1300790000000021	F	F	F
0.7833329999999989	0.2444440000000014	0.2276979999999966	F	F	F
0.6164865981326333	0.0220109659704277	0.3231228974664831	T	T	T
0.9504295239518732	0.1335854268534450	0.4223811799594016	T	T	T
0.7000000000000028	0.1333330000000004	0.1300790000000021	F	F	F
0.5333329999999989	0.2444440000000014	0.2276979999999966	F	F	F
0.8664865372017408	0.0220109474369886	0.3231231561041141	T	T	T

0.7004293552810822	0.1335854819195880	0.4223810632269286	T	T	T
0.7000000000000028	0.2999999999999972	0.1300790000000021	F	F	F
0.5333329999999989	0.0777780000000021	0.2276979999999966	F	F	F
0.8664859694614013	0.1886774942651039	0.3231237389575594	T	T	T
0.7004296864556839	0.3002526432382497	0.4223818350488481	T	T	T
0.4500000000000028	0.6333330000000004	0.1300790000000021	F	F	F
0.2833329999999989	0.4111109999999982	0.2276979999999966	F	F	F
0.1164997822982487	0.5220289510543730	0.3231230728302458	T	T	T
0.4504338271818129	0.6335922376367130	0.4223806851677085	T	T	T
0.4500000000000028	0.4666670000000011	0.1300790000000021	F	F	F
0.2833329999999989	0.5777780000000021	0.2276979999999966	F	F	F
0.1164918437714761	0.3553531341301450	0.3231236022569551	T	T	T
0.4504322288423558	0.4669226489903313	0.4223818721694723	T	T	T
0.2000000000000028	0.4666670000000011	0.1300790000000021	F	F	F
0.0333329999999989	0.5777780000000021	0.2276979999999966	F	F	F
0.3664921392056135	0.3553530211602369	0.3231235036325392	T	T	T
0.2004321936455960	0.4669227497455790	0.4223819209257641	T	T	T
0.2000000000000028	0.6333330000000004	0.1300790000000021	F	F	F
0.0333329999999989	0.4111109999999982	0.2276979999999966	F	F	F
0.3664995951252924	0.5220289321085985	0.3231231547524115	T	T	T
0.2004340167873059	0.6335920930386940	0.4223812891159585	T	T	T
0.9500000000000028	0.6333330000000004	0.1300790000000021	F	F	F
0.7833329999999989	0.4111109999999982	0.2276979999999966	F	F	F
0.6164992840101648	0.5220289465763880	0.3231232184779227	T	T	T
0.9504337481190260	0.6335921157852065	0.4223814686717056	T	T	T
0.9500000000000028	0.4666670000000011	0.1300790000000021	F	F	F
0.7833329999999989	0.5777780000000021	0.2276979999999966	F	F	F
0.6164916903164321	0.3553529283631993	0.3231233438324362	T	T	T
0.9504321411138701	0.4669227284696011	0.4223810229672957	T	T	T
0.7000000000000028	0.4666670000000011	0.1300790000000021	F	F	F
0.5333329999999989	0.5777780000000021	0.2276979999999966	F	F	F
0.8664917518520789	0.3553528444830035	0.3231234526056611	T	T	T
0.7004324185809084	0.4669228089183337	0.4223810355046305	T	T	T
0.7000000000000028	0.6333330000000004	0.1300790000000021	F	F	F
0.5333329999999989	0.4111109999999982	0.2276979999999966	F	F	F
0.8664995316714010	0.5220289726879030	0.3231227083036228	T	T	T
0.7004340368653847	0.6335921680923079	0.4223816679890103	T	T	T
0.4500000000000028	0.9666670000000011	0.1300790000000021	F	F	F
0.2833329999999989	0.7444440000000014	0.2276979999999966	F	F	F
0.1164926263671333	0.8553530299079861	0.3231227430587112	T	T	T
0.4504312672106882	0.9669217474723324	0.4223801931810970	T	T	T
0.4500000000000028	0.7999999999999972	0.1300790000000021	F	F	F
0.2833329999999989	0.9111109999999982	0.2276979999999966	F	F	F
0.1164998907537902	0.6886956819625824	0.3231232389638466	T	T	T

```
0.4504334114181259   0.8002583596712853   0.4223812931435453   T   T   T
0.2000000000000028   0.7999999999999972   0.1300790000000021   F   F   F
0.0333329999999989   0.9111109999999982   0.2276979999999966   F   F   F
0.3664998292859183   0.6886958132645878   0.3231229852990304   T   T   T
0.2004336634880751   0.8002585580422740   0.4223808603821465   T   T   T
0.2000000000000028   0.9666670000000011   0.1300790000000021   F   F   F
0.0333329999999989   0.7444440000000014   0.2276979999999966   F   F   F
0.3664927576588190   0.8553531322566286   0.3231229511788322   T   T   T
0.2004317005859667   0.9669216076569400   0.4223806402621327   T   T   T
0.9500000000000028   0.9666670000000011   0.1300790000000021   F   F   F
0.7833329999999989   0.7444440000000014   0.2276979999999966   F   F   F
0.6164927550848935   0.8553532146488029   0.3231230034011286   T   T   T
0.9504313895093541   0.9669216205432433   0.4223807432662628   T   T   T
0.9500000000000028   0.7999999999999972   0.1300790000000021   F   F   F
0.7833329999999989   0.9111109999999982   0.2276979999999966   F   F   F
0.6165002455170714   0.6886957414843372   0.3231230654641338   T   T   T
0.9504336936261797   0.8002583782846051   0.4223808489784261   T   T   T
0.7000000000000028   0.7999999999999972   0.1300790000000021   F   F   F
0.5333329999999989   0.9111109999999982   0.2276979999999966   F   F   F
0.8664999042676518   0.6886956617169706   0.3231232868943167   T   T   T
0.7004335743892101   0.8002584619020755   0.4223805933304606   T   T   T
0.7000000000000028   0.9666670000000011   0.1300790000000021   F   F   F
0.5333329999999989   0.7444440000000014   0.2276979999999966   F   F   F
0.8664931064764676   0.8553532322575268   0.3231227483429509   T   T   T
0.7004317245688695   0.9669216445518140   0.4223808215966863   T   T   T
```

```
PdH_Pdopt
  1.00000000000000
    10.1921769999999992    -5.8844560000000001     0.0000000000000000
     0.0000000000000000    17.6533690000000014     0.0000000000000000
     0.0000000000000000     0.0000000000000000    24.6092779999999998
  H    Pd
   96   120
Selective dynamics
Direct
0.4333299999999980   0.0111100000000022   0.1300799999999995   F   F   F
0.2666699999999977   0.1222199999999987   0.2276999999999987   F   F   F
0.1044654678184499   0.2351665353146666   0.3373603867800049   T   T   T
0.4331992990089801   0.0106227501416456   0.4516071212325776   T   T   T
0.4333299999999980   0.1777799999999985   0.1300799999999995   F   F   F
0.2666699999999977   0.2888900000000021   0.2276999999999987   F   F   F
0.1045516336492141   0.0685611864765154   0.3373132205143624   T   T   T
0.4330463488994966   0.1772248096465569   0.4517172012613999   T   T   T
0.1833299999999980   0.1777799999999985   0.1300799999999995   F   F   F
```

0.0166699999999977	0.2888900000000021	0.2276999999999987	F	F	F
0.3544880364130914	0.0684586416260951	0.3373159925687032	T	T	T
0.1830785302782806	0.1772599011071300	0.4517073141177146	T	T	T
0.1833299999999980	0.0111100000000022	0.1300799999999995	F	F	F
0.0166699999999977	0.1222199999999987	0.2276999999999987	F	F	F
0.3544153628438800	0.2350852526803114	0.3373603597176382	T	T	T
0.1832075721375505	0.0106780791210855	0.4515636198064386	T	T	T
0.9333299999999980	0.0111100000000022	0.1300799999999995	F	F	F
0.7666699999999977	0.1222199999999987	0.2276999999999987	F	F	F
0.6044351256554157	0.2350994974876132	0.3373505595044928	T	T	T
0.9334346912148035	0.0107077427159919	0.4515095794366332	T	T	T
0.9333299999999980	0.1777799999999985	0.1300799999999995	F	F	F
0.7666699999999977	0.2888900000000021	0.2276999999999987	F	F	F
0.6044773306664951	0.0684757582529156	0.3373198066884157	T	T	T
0.9333138891681081	0.1773482087765820	0.4516134605000221	T	T	T
0.6833299999999980	0.1777799999999985	0.1300799999999995	F	F	F
0.5166699999999977	0.2888900000000021	0.2276999999999987	F	F	F
0.8545293343448258	0.0685325792260702	0.3372941940312446	T	T	T
0.6832146756955317	0.1772719317275719	0.4517527210752319	T	T	T
0.6833299999999980	0.0111100000000022	0.1300799999999995	F	F	F
0.5166699999999977	0.1222199999999987	0.2276999999999987	F	F	F
0.8544608167456915	0.2351438505411197	0.3373377881744536	T	T	T
0.6833922373187665	0.0106999363307422	0.4516874781863222	T	T	T
0.4333299999999980	0.3444399999999987	0.1300799999999995	F	F	F
0.2666699999999977	0.4555599999999984	0.2276999999999987	F	F	F
0.1045099561894133	0.5685073716578259	0.3373556676929056	T	T	T
0.4331160662734581	0.3439625846919774	0.4518085853513988	T	T	T
0.4333299999999980	0.5111100000000022	0.1300799999999995	F	F	F
0.2666699999999977	0.6222199999999987	0.2276999999999987	F	F	F
0.1044607247782850	0.4017760751419172	0.3373707372399523	T	T	T
0.4332498692072645	0.5106632468553979	0.4515099977186698	T	T	T
0.1833299999999980	0.5111100000000022	0.1300799999999995	F	F	F
0.0166699999999977	0.6222199999999987	0.2276999999999987	F	F	F
0.3543566346760032	0.4016908072546562	0.3373625063174403	T	T	T
0.1832385272426924	0.5106104792403401	0.4514932532915494	T	T	T
0.1833299999999980	0.3444399999999987	0.1300799999999995	F	F	F
0.0166699999999977	0.4555599999999984	0.2276999999999987	F	F	F
0.3544303772957810	0.5684099655068977	0.3373628870988943	T	T	T
0.1832375846494976	0.3439755406424059	0.4517808013884321	T	T	T
0.9333299999999980	0.3444399999999987	0.1300799999999995	F	F	F
0.7666699999999977	0.4555599999999984	0.2276999999999987	F	F	F
0.6044583094751613	0.5684619932562034	0.3373496426400577	T	T	T
0.9334170001294000	0.3440664789876759	0.4517406275486541	T	T	T
0.9333299999999980	0.5111100000000022	0.1300799999999995	F	F	F

0.7666699999999977	0.6222199999999987	0.2276999999999987	F	F	F
0.6043851827113528	0.4017191687745599	0.3373595952731488	T	T	T
0.9333842008297113	0.5107408915169277	0.4513823804716702	T	T	T
0.6833299999999980	0.5111100000000022	0.1300799999999995	F	F	F
0.5166699999999977	0.6222199999999987	0.2276999999999987	F	F	F
0.8544139173344818	0.4017608098373683	0.3373568625305389	T	T	T
0.6833725739812385	0.5106581913766248	0.4516041203679049	T	T	T
0.6833299999999980	0.3444399999999987	0.1300799999999995	F	F	F
0.5166699999999977	0.4555599999999984	0.2276999999999987	F	F	F
0.8544440727264381	0.5684859076604336	0.3373350459209848	T	T	T
0.6833389516693953	0.3439921925197925	0.4518736373096688	T	T	T
0.4333299999999980	0.6777799999999985	0.1300799999999995	F	F	F
0.2666699999999977	0.7888900000000021	0.2276999999999987	F	F	F
0.1043903141722117	0.9017969164153139	0.3373340614162519	T	T	T
0.4330695317806177	0.6771591645971925	0.4516978947670227	T	T	T
0.4333299999999980	0.8444399999999987	0.1300799999999995	F	F	F
0.2666699999999977	0.9555599999999984	0.2276999999999987	F	F	F
0.1043845559802965	0.7351051178744487	0.3373625618257042	T	T	T
0.4332207938977358	0.8439455152749569	0.4518689823622080	T	T	T
0.1833299999999980	0.8444399999999987	0.1300799999999995	F	F	F
0.0166699999999977	0.9555599999999984	0.2276999999999987	F	F	F
0.3543381374145853	0.7350619395447023	0.3373539954188589	T	T	T
0.1831370932641648	0.8439273298703847	0.4518390850263275	T	T	T
0.1833299999999980	0.6777799999999985	0.1300799999999995	F	F	F
0.0166699999999977	0.7888900000000021	0.2276999999999987	F	F	F
0.3543375600154927	0.9017383802770466	0.3373265376824063	T	T	T
0.1830932421366673	0.6771429249259525	0.4518455842822030	T	T	T
0.9333299999999980	0.6777799999999985	0.1300799999999995	F	F	F
0.7666699999999977	0.7888900000000021	0.2276999999999987	F	F	F
0.6043458914316074	0.9017382069094519	0.3373461441562965	T	T	T
0.9332145513072335	0.6772086188925726	0.4516239161143499	T	T	T
0.9333299999999980	0.8444399999999987	0.1300799999999995	F	F	F
0.7666699999999977	0.9555599999999984	0.2276999999999987	F	F	F
0.6043151112419185	0.7350585783450585	0.3373497986961442	T	T	T
0.9332843520237725	0.8439516280707281	0.4517917586448091	T	T	T
0.6833299999999980	0.8444399999999987	0.1300799999999995	F	F	F
0.5166699999999977	0.9555599999999984	0.2276999999999987	F	F	F
0.8543241971173708	0.7351106474179918	0.3373439936138691	T	T	T
0.6832725215003138	0.8439085508996617	0.4518924866559034	T	T	T
0.6833299999999980	0.6777799999999985	0.1300799999999995	F	F	F
0.5166699999999977	0.7888900000000021	0.2276999999999987	F	F	F
0.8543676035806171	0.9017870034451337	0.3373219523494010	T	T	T
0.6831065836444902	0.6771365689389107	0.4518109262123760	T	T	T
0.2666699999999977	0.2888900000000021	0.0812700000000035	F	F	F

0.1000000000000014	0.0666700000000020	0.1788900000000027	F	F	F
0.4333299999999980	0.1777799999999985	0.2765100000000018	F	F	F
0.2675716542827919	0.2876751016401758	0.3712713753355784	T	T	T
0.0996048507857641	0.0660032544291466	0.4697213712507778	T	T	T
0.2666699999999977	0.1222199999999987	0.0812700000000035	F	F	F
0.1000000000000014	0.2333300000000023	0.1788900000000027	F	F	F
0.4333299999999980	0.0111100000000022	0.2765100000000018	F	F	F
0.2676329612933542	0.1210591376343560	0.3712258440099466	T	T	T
0.0996996602930579	0.2326543671547765	0.4696885308785478	T	T	T
0.0166699999999977	0.1222199999999987	0.0812700000000035	F	F	F
0.3500000000000014	0.2333300000000023	0.1788900000000027	F	F	F
0.1833299999999980	0.0111100000000022	0.2765100000000018	F	F	F
0.0176906163910150	0.1210552653873049	0.3712429703546875	T	T	T
0.3499160279978797	0.2326427501215311	0.4696787695271915	T	T	T
0.0166699999999977	0.2888900000000021	0.0812700000000035	F	F	F
0.3500000000000014	0.0666700000000020	0.1788900000000027	F	F	F
0.1833299999999980	0.1777799999999985	0.2765100000000018	F	F	F
0.0176420777033999	0.2876866667078166	0.3712741746129323	T	T	T
0.3499084315438007	0.0660396295957168	0.4697599378343801	T	T	T
0.7666699999999977	0.2888900000000021	0.0812700000000035	F	F	F
0.6000000000000014	0.0666700000000020	0.1788900000000027	F	F	F
0.9333299999999980	0.1777799999999985	0.2765100000000018	F	F	F
0.7676079002180531	0.2876784754520165	0.3712811561380314	T	T	T
0.6000242009637718	0.0661348680626533	0.4696702822218254	T	T	T
0.7666699999999977	0.1222199999999987	0.0812700000000035	F	F	F
0.6000000000000014	0.2333300000000023	0.1788900000000027	F	F	F
0.9333299999999980	0.0111100000000022	0.2765100000000018	F	F	F
0.7676553252518410	0.1210822727401128	0.3712568740902555	T	T	T
0.5999023555808856	0.2326535375545809	0.4695983200725845	T	T	T
0.5166699999999977	0.1222199999999987	0.0812700000000035	F	F	F
0.8500000000000014	0.2333300000000023	0.1788900000000027	F	F	F
0.6833299999999980	0.0111100000000022	0.2765100000000018	F	F	F
0.5175718916067275	0.1210064612054693	0.3712461097477402	T	T	T
0.8496708646256017	0.2325764554026971	0.4696064815290153	T	T	T
0.5166699999999977	0.2888900000000021	0.0812700000000035	F	F	F
0.8500000000000014	0.0666700000000020	0.1788900000000027	F	F	F
0.6833299999999980	0.1777799999999985	0.2765100000000018	F	F	F
0.5175661478475306	0.2876796947720723	0.3712560355235798	T	T	T
0.8498517485429958	0.0660672024444481	0.4696741304223883	T	T	T
0.2666699999999977	0.6222199999999987	0.0812700000000035	F	F	F
0.1000000000000014	0.3999999999999986	0.1788900000000027	F	F	F
0.4333299999999980	0.5111100000000022	0.2765100000000018	F	F	F
0.2675733663721952	0.6209361890470859	0.3712048426779423	T	T	T
0.0995894956585356	0.3992355509897514	0.4696470338844795	T	T	T

0.2666699999999977	0.4555599999999984	0.0812700000000035	F	F	F
0.1000000000000014	0.5666700000000020	0.1788900000000027	F	F	F
0.4333299999999980	0.3444399999999987	0.2765100000000018	F	F	F
0.2675794342713141	0.4543527610834784	0.3712436451522766	T	T	T
0.0995709437499892	0.5659587263421763	0.4697071900889339	T	T	T
0.0166699999999977	0.4555599999999984	0.0812700000000035	F	F	F
0.3500000000000014	0.5666700000000020	0.1788900000000027	F	F	F
0.1833299999999980	0.3444399999999987	0.2765100000000018	F	F	F
0.0175780718144284	0.4543245407461156	0.3712582118658052	T	T	T
0.3498073280573087	0.5659296833087656	0.4697906086643022	T	T	T
0.0166699999999977	0.6222199999999987	0.0812700000000035	F	F	F
0.3500000000000014	0.3999999999999986	0.1788900000000027	F	F	F
0.1833299999999980	0.5111100000000022	0.2765100000000018	F	F	F
0.0175831157783279	0.6209490297652704	0.3712455314422446	T	T	T
0.3498229316359341	0.3992058552837174	0.4696713033579606	T	T	T
0.7666699999999977	0.6222199999999987	0.0812700000000035	F	F	F
0.6000000000000014	0.3999999999999986	0.1788900000000027	F	F	F
0.9333299999999980	0.5111100000000022	0.2765100000000018	F	F	F
0.7675871934030790	0.6209526461921352	0.3712415625916266	T	T	T
0.5999095454162313	0.3993466167173791	0.4695512633706405	T	T	T
0.7666699999999977	0.4555599999999984	0.0812700000000035	F	F	F
0.6000000000000014	0.5666700000000020	0.1788900000000027	F	F	F
0.9333299999999980	0.3444399999999987	0.2765100000000018	F	F	F
0.7675761395887848	0.4543499132197522	0.3712746435639661	T	T	T
0.5999047935497004	0.5659297765562039	0.4696744470328142	T	T	T
0.5166699999999977	0.4555599999999984	0.0812700000000035	F	F	F
0.8500000000000014	0.5666700000000020	0.1788900000000027	F	F	F
0.6833299999999980	0.3444399999999987	0.2765100000000018	F	F	F
0.5175474423633657	0.4543774801602489	0.3712470475252764	T	T	T
0.8497817663383153	0.5658392633619345	0.4697141006091261	T	T	T
0.5166699999999977	0.6222199999999987	0.0812700000000035	F	F	F
0.8500000000000014	0.3999999999999986	0.1788900000000027	F	F	F
0.6833299999999980	0.5111100000000022	0.2765100000000018	F	F	F
0.5175001429301480	0.6209452042562957	0.3712124042538857	T	T	T
0.8497585629484109	0.3992474499599150	0.4695479463801729	T	T	T
0.2666699999999977	0.9555599999999984	0.0812700000000035	F	F	F
0.1000000000000014	0.7333300000000023	0.1788900000000027	F	F	F
0.4333299999999980	0.8444399999999987	0.2765100000000018	F	F	F
0.2675666005348685	0.9543341745920031	0.3712355517922097	T	T	T
0.0995716119462562	0.7325433224674925	0.4696306978720370	T	T	T
0.2666699999999977	0.7888900000000021	0.0812700000000035	F	F	F
0.1000000000000014	0.8999999999999986	0.1788900000000027	F	F	F
0.4333299999999980	0.6777799999999985	0.2765100000000018	F	F	F
0.2675356001326215	0.7876566200482996	0.3712398626311514	T	T	T

0.0996106355618195	0.8992457085769248	0.4696030198070614	T	T	T
0.0166699999999977	0.7888900000000021	0.0812700000000035	F	F	F
0.3500000000000014	0.8999999999999986	0.1788900000000027	F	F	F
0.1833299999999980	0.6777799999999985	0.2765100000000018	F	F	F
0.0176217009614982	0.7876631704948543	0.3712701513692889	T	T	T
0.3499392018607256	0.8993338310256425	0.4696623735585204	T	T	T
0.0166699999999977	0.9555599999999984	0.0812700000000035	F	F	F
0.3500000000000014	0.7333300000000023	0.1788900000000027	F	F	F
0.1833299999999980	0.8444399999999987	0.2765100000000018	F	F	F
0.0175886195730601	0.9543817043308096	0.3712717770707751	T	T	T
0.3499397359051371	0.7325654113044804	0.4696682299899358	T	T	T
0.7666699999999977	0.9555599999999984	0.0812700000000035	F	F	F
0.6000000000000014	0.7333300000000023	0.1788900000000027	F	F	F
0.9333299999999980	0.8444399999999987	0.2765100000000018	F	F	F
0.7675902358159787	0.9543515421743886	0.3712842145324992	T	T	T
0.5998887370901260	0.7326175082610478	0.4696137298052179	T	T	T
0.7666699999999977	0.7888900000000021	0.0812700000000035	F	F	F
0.6000000000000014	0.8999999999999986	0.1788900000000027	F	F	F
0.9333299999999980	0.6777799999999985	0.2765100000000018	F	F	F
0.7676132475657290	0.7876628477249273	0.3712802113958902	T	T	T
0.6000504562340842	0.8994138577006275	0.4695695862468869	T	T	T
0.5166699999999977	0.7888900000000021	0.0812700000000035	F	F	F
0.8500000000000014	0.8999999999999986	0.1788900000000027	F	F	F
0.6833299999999980	0.6777799999999985	0.2765100000000018	F	F	F
0.5175928129952548	0.7877064641403030	0.3712554724370525	T	T	T
0.8497923928721046	0.8993240268987497	0.4695864226819810	T	T	T
0.5166699999999977	0.9555599999999984	0.0812700000000035	F	F	F
0.8500000000000014	0.7333300000000023	0.1788900000000027	F	F	F
0.6833299999999980	0.8444399999999987	0.2765100000000018	F	F	F
0.5175440482225201	0.9543603972211568	0.3712530134140472	T	T	T
0.8498492781658186	0.7326397018185300	0.4696111949723835	T	T	T
